# Taming boroloborinines: toward photostable polycyclic antiaromatic hydrocarbons

**DOI:** 10.1039/d5sc05880e

**Published:** 2026-01-20

**Authors:** Muhammad Yasir Mehboob, Minu Sheeja, Mahdi Sasar, Cina Foroutan-Nejad

**Affiliations:** a Institute of Organic Chemistry, Polish Academy of Sciences Kasprzaka 44/52 01-224 Warsaw Poland cina.foroutan-nejad@icho.edu.pl

## Abstract

Boron-containing heterocycles are attracting growing attention due to their unique electronic structures and effectiveness as electron acceptors in functional organic materials. Among them, boroloborinines, a fused borole–borinine scaffold, constitute a rare class of 8π-electron antiaromatic systems with potential applications in organic electronics. However, their inherent antiaromatic instability has limited synthetic exploration and practical deployment. In this work, we employ density functional theory (DFT) along with wavefunction-based methods to systematically investigate strategies for stabilizing boroloborinine derivatives *via* nitrogen incorporation and selective benzannulation. Photochemical stability is evaluated using HOMO–LUMO and singlet–triplet (S–T) energy gaps, while aromaticity is assessed through three classes of indices: the multicenter index (MCI), the Harmonic Oscillator Model of Aromaticity (HOMA), and magnetically induced current density (MICD). Our findings show that both the position and nature of substitution critically influence the electronic structure, with the formation of Clar's sextets correlating strongly with increased stability. Molecules that exhibit reduced antiaromaticity in the singlet state and minimal aromaticity in the triplet state tend to possess the largest S–T gaps. Besides photostability, we examined proton affinity of our model systems to verify which molecules can remain stable in acidic environments. Our modeling suggests that nitrogen atoms in the vicinity of boron have higher proton affinity; therefore, they are potentially more prone to acid catalyzed reactions. These insights provide guiding principles for designing antiaromatic chromophores with enhanced stability and tunable optoelectronic properties, potentially enabling the development of novel organic emitters with distinct emission wavelengths and improved quantum yields.

## Abstract 2

We computationally explore strategies to stabilize boroloborinines, a rare 8π-electron antiaromatic borole–borinine scaffold, revealing design principles for photostable antiaromatic chromophores in organic electronics.

## Graphical abstract

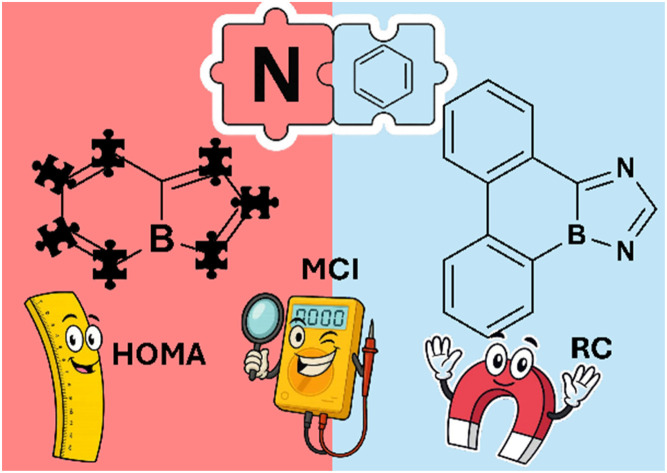

## Introduction

**Fig. 1:**
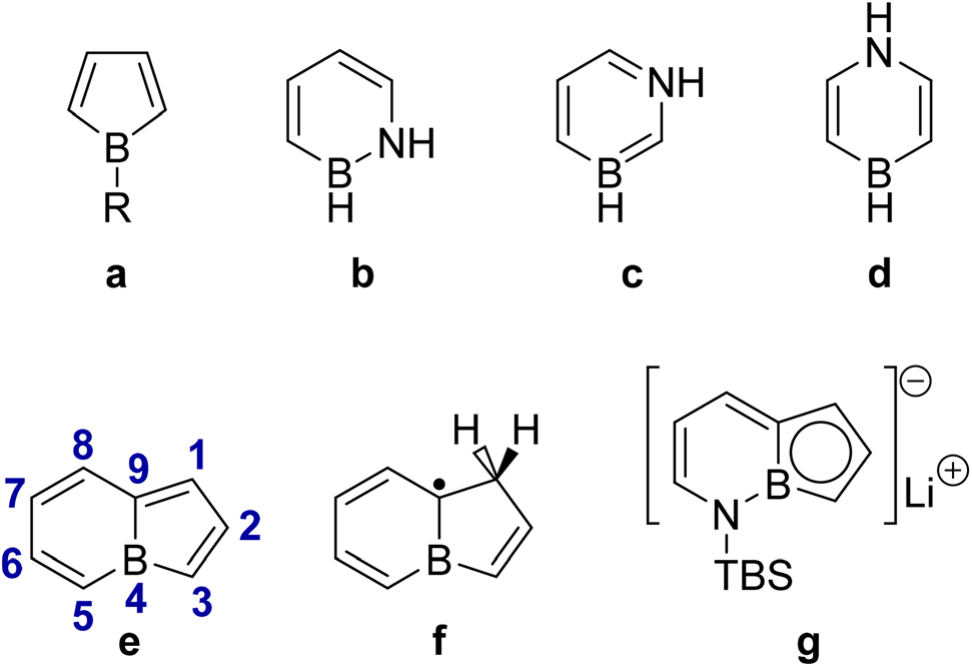
The structures of (a) borole, (b) 1,2-dihydro 1,2-azaborine, (c) 1,3-dihydro 1,3-azaborine, (d) 1,4-dihydro 1,4-azaborine, (e) boroloborinines and its numbering, (f) 7H-borolo[1,2-a]borinine radical, and (g) lithium salt of the borolo[1,2-b][1,2]azaborininyl anion.

Boron-containing heterocycles play a key role in various synthetic transformations and are frequently utilized as electron-acceptor units in donor–acceptor dyes based on polyaromatic hydrocarbons (PAHs) for applications in organic electronics.^1–15^ The inherent p-electron deficiency of boron makes it a valuable element in the synthetic chemist's toolbox for modulating the aromatic and antiaromatic character of its heterocyclic hydrocarbons. Among the diverse boron-containing heterocycles, boroles constitute a family of five-membered hydrocarbons that were first isolated in the late 1960s.^16^ They are classified as antiaromatic organic molecules,^17–22^ obeying the 4π Hückel rule and exhibiting a relatively low singlet–triplet energy gap as a result of Baird aromaticity^23^ of their excited states (Fig. 1a).^24^ On the other hand, the six-membered counterparts of boroles, borinines, can easily become isoelectronic with benzene if a heteroatom such as oxygen or nitrogen compensates for the electron deficiency of the boron atoms in their rings (Fig. 1b–d).^25–30^ The fusion of borole and borinine rings results in a lesser-known 8π-electron antiaromatic bicyclic system known as borolo[1,2-a]borinines, hereafter referred to as boroloborinines (Fig. 1e).

In recent years, antiaromatic dyes have emerged as a promising concept in organic electronics, offering a novel approach for the design of near-infrared (NIR) emissive materials.^31,32^ Consequently, developing new approaches for accessing potential antiaromatic chromophores is an appealing objective in contemporary chemistry. Despite the diversity and success of synthetic pathways to boroles and borinines, boroloborinines and their derivatives are rarely discussed in the literature, likely due to their inherent instability arising from their 8π-electron antiaromatic nature. One of the closest reported structures to the free boroloborinine scaffold is the 7H-borolo[1,2-a]borinine radical (Fig. 1f) obtained from photoinduced boron atom insertion in the benzocyclobutene moiety.^33^ In a separate study, the structure of the 10π-electron aromatic borolo[1,2-b][1,2]azaborininyl anion (Fig. 1g), an azaaromatic counterpart of boroloborinines, was synthesized and isolated *via* cross-coupling as a lithium salt.^28^ The boroloborinine scaffold has been stabilized within the structure of large polycyclic aromatic hydrocarbons and is found to retain its antiaromatic properties and represent photo-switchable Lewis acidity.^34^ Achieving the core structure of boroloborinine requires enhancing its stability through stereoelectronic engineering.

In the present work, we have systematically investigated strategies for stabilizing the boroloborinine scaffold through nitrogen incorporation and selective benzannulation to identify synthetically accessible derivatives with enhanced stability within this class of compounds. In a recent study, we demonstrated that selective benzannulation can increase the energy gap between the highest occupied and lowest unoccupied molecular orbitals (HOMO–LUMO gap) by introducing resonance structures that feature Clar sextets.^35^ We assess the stability of boroloborinine derivatives using two key parameters: (1) the HOMO–LUMO and the singlet–triplet (S–T) energy gaps that define photostability. These factors are indicative of photochemical stability and the propensity of the molecules to undergo radical processes *via* photochemically accessible triplet states; (2) proton affinity that defines stability of the molecules in acidic environments.

**Fig. 2:**
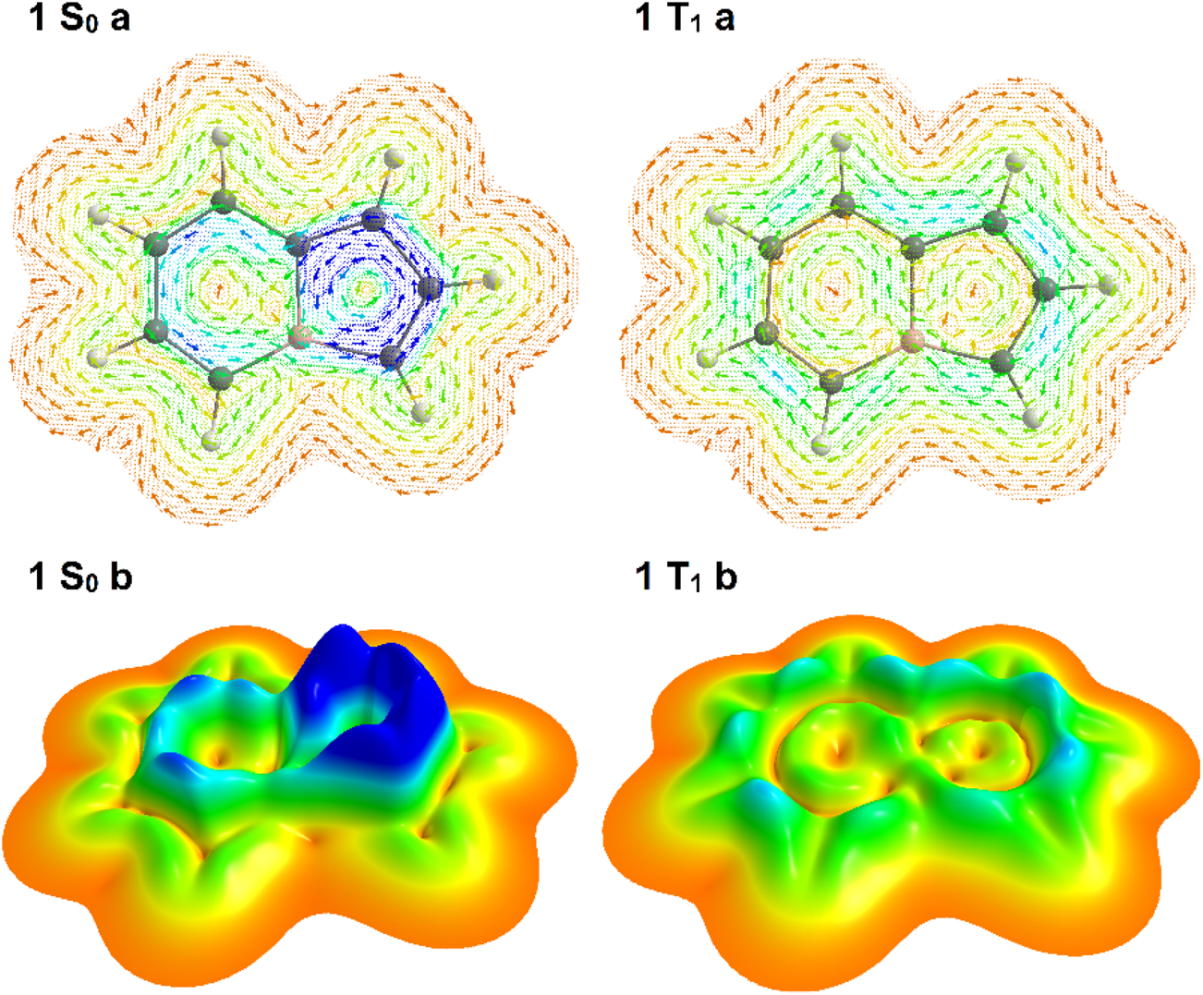
Ring current maps of the singlet ground state (*S*_0_) and the lowest triplet state (*T*_1_) of boroloborinine (molecule 1) 1 au above the ring plane, where the π-current is the strongest. (a) Vector plots illustrating magnetically induced ring currents: a strong counterclockwise current is observed in the interior region of the *S*_0_ state, indicative of magnetic antiaromaticity, while a clockwise current on the periphery of the *T*_1_ state suggests magnetic aromaticity. (b) Relief maps corresponding to the ring current distribution provide spatial visualization of current intensity. In these maps, dark blue denotes the strongest currents (>0.0008 a.u), while dark orange indicates minimal current approaching zero. The height of each peak corresponds to the local current intensity across the molecular framework.

Monocyclic (anti)aromatic compounds follow Hückel's rule in their ground states and Baird's rule in their lowest singlet and triplet excited states.^23,36,37^ Accordingly, a monocyclic aromatic species becomes antiaromatic in its first excited singlet and triplet states, whereas an antiaromatic molecule often gains aromatic character upon excitation. While Hückel's and Baird's rules can sometimes be extended to polycyclic systems, such generalizations are not always straightforward. The 8π-electron framework of boroloborinines renders them antiaromatic in their singlet ground states, consistent with Hückel's rule. However, to the best of our knowledge, the possible Baird aromaticity of these species in their lowest triplet states has not yet been investigated. Ring current analyses (Fig. 2), along with other aromaticity indices (*vide infra*), indicate that the parent boroloborinine also adheres to Baird's rule in its triplet state.

A possible strategy to improve the stability of these molecules is to identify systems that exhibit reduced antiaromaticity in the singlet ground state and, simultaneously, either entirely or partially disobey Baird's rule and represent a diminished aromatic stabilization in the triplet state. This would raise the relative energy of the triplet state and reduce the likelihood of undesired radical reactions. This scenario is reminiscent of the mechanism underlying the exceptional photostability of the H_3_^+^ cation.^38^ Throughout our screening we employ density functional theory as a tool that balances accuracy and speed for identification of potential synthetic targets for future studies. In addition, we performed coupled-cluster calculations to benchmark the DFT results. Comparison of singlet–triplet energy gaps obtained at two DFT levels (M06-2X/def2-TZVPP and CAM-B3LYP/def2-TZVPP) against CCSD(T)/def2-SVP//CCSD/def2-SVP reference data indicates that M06-2X/def2-TZVPP yields smaller deviations. Therefore, this level of theory was selected for all subsequent molecular property calculations. The details of the computational methods are listed in the SI.

In this work, we also examine to what extent commonly used aromaticity indices can help identify stable species by correlating various aromaticity measures with stability indicators. To assess aromaticity, we employed indices from three distinct classes: magnetically induced current intensity (MICD),^39,40^ the Harmonic Oscillator Model of Aromaticity (HOMA),^41^ and the multicenter index (MCI).^42^ It is worth noting that among the employed aromaticity probes, HOMA might not be ideal for assessing the aromatic character of molecules in their excited states.^43^ However, structural indices parameterized for excited states such as HOMER^43^ are not parametrized for C–B bonds. Nevertheless, as we will show in the following sections, HOMA does provide some insight into the trends of the aromaticity variation excitation to the triplet state. Energetic indices were not used in this study, as the structural diversity of our model systems precludes a uniform, energy-based assessment of aromaticity. In the absence of such a consistent framework, the energetic data would not allow for reliable comparisons across different systems. Our results offer guidelines for designing stable antiaromatic molecules incorporating the boroloborinine core and open new avenues toward novel photochemically active materials.

## Results and discussion

### Aromaticity and photostability among nitrogen doped bicyclic molecules

To establish a reference for evaluating the (anti)aromatic character of boroloborinines, we examined the aromaticity of borole, pyrrole, and pyridine, three monocyclic systems structurally related to boroloborinines and their aza-derivatives. Table 1 summarizes the aromaticity characteristics of these reference molecules. Positive HOMA values approaching unity, positive magnetically induced current densities (MICD), and large MCI values close to one are known to be indicative of aromaticity, whereas negative HOMA or MICD values and small MCI values near zero signify antiaromaticity. In all cases, the aromatic character changes between the ground and triplet excited states. For borole, all indices increase in magnitude and become positive, indicating the onset of aromaticity in the triplet state. For pyrrole, all indices clearly denote antiaromatic behavior in the excited triplet state. Pyridine, however, behaves differently from the other two systems: while the ring-current-based index suggests antiaromatic character, both MCI and HOMA indicate that in the first triplet excited state, aromaticity is only partially reduced rather than completely lost.

**Table 1:** Indices reflecting the aromaticity of borole, pyrrole, and pyridine in their ground and excited states of these molecules

Molecules	Singlet			Triplet		
	MCI	MICD	HOMA	MCI	MICD	HOMA
Borole	0.36	−10.9	−1.14	0.49	8.1	0.16
Pyrrole	0.56	12.4	0.87	0.31	−5.4	−0.13
Pyridine	0.63	12.1	1.0	0.51	−5.2	0.75

**Fig. 3:**
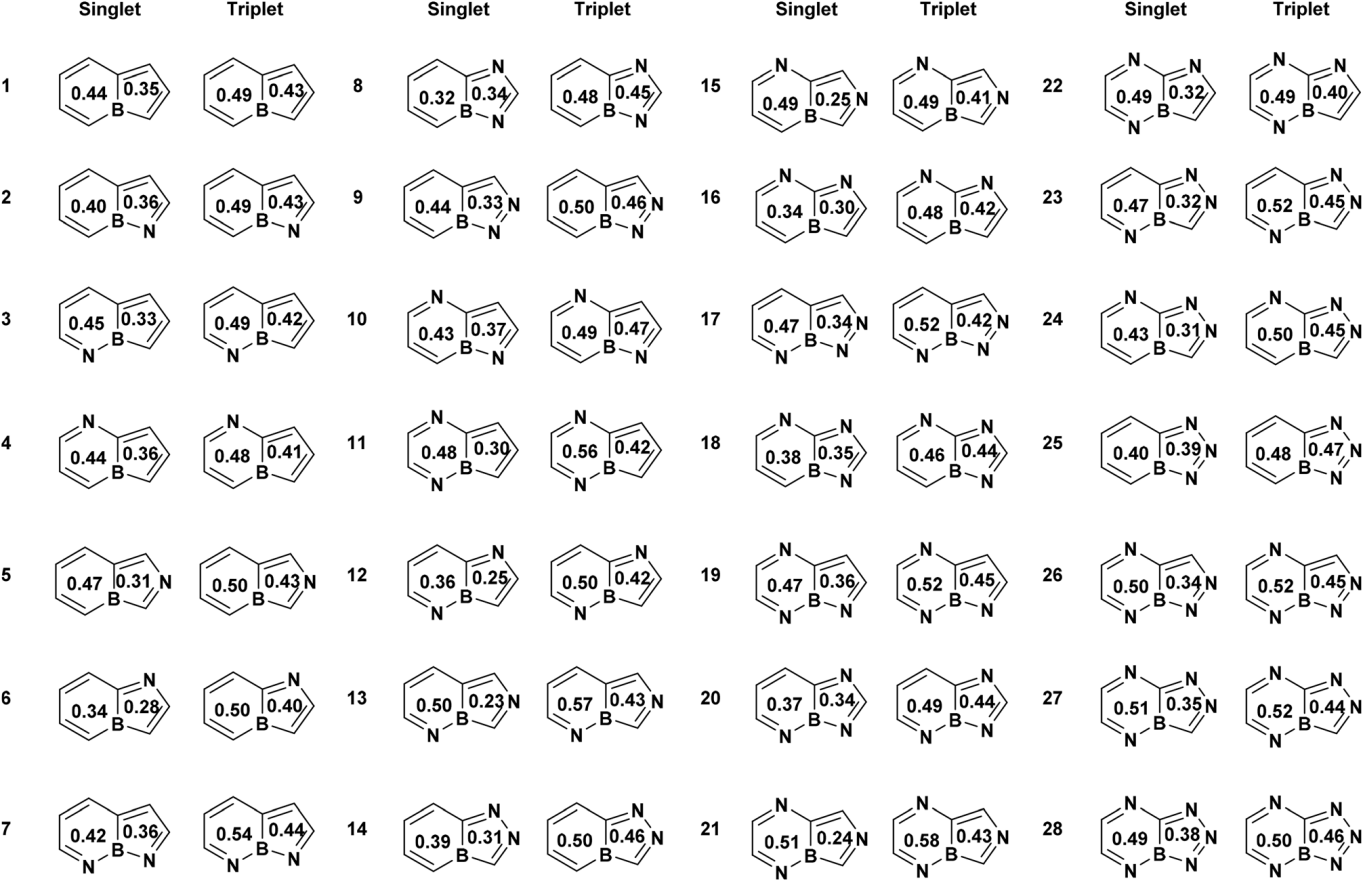
Structures of nitrogen-containing derivatives of boroloborinine. Each ring is annotated with its normalized multicenter index (MCI) value.

We began our investigation by examining 30 nitrogen-containing derivatives of boroloborinine in their ground-state singlet and lowest-energy triplet configurations. During geometry optimization, two of the singlet structures underwent ring opening. Therefore, our analysis focused on the remaining 28 molecules, as depicted in Fig. 3. Examination of these structures suggests that in most cases, significant geometric distortion leads to σ–π mixing, which limits the applicability of Hückel's and Baird's rules in determining the (anti)aromatic character of these species by reducing aromatic or antiaromatic character. This phenomenon is more commonly observed in species with more than 2 nitrogen atoms, in particular, when a nitrogen atom is positioned adjacent to boron within the borinine moiety at position 5. Indeed only 14 molecules in the sets (1 to 9, 12, 14, 16, 20 and 25) are planar, thereby sustaining unperturbed (anti)aromaticity. Among the remaining molecules, the borole ring exhibits significant deviations from planarity. An analysis of nonplanarity, quantified by the dihedral angles on both sides of the bond shared between the borole and borinine rings (dihedrals between atoms 1, 9, 4, and 3, and 8, 9, 4, and 5 as shown in Fig. 1e), indicates that the borinine rings are generally more planar than their borole counterparts (Table S2). Across all 28 molecules, the borinine rings show an average dihedral angle of 2.7°, increasing to 5.4° among the nonplanar cases. In contrast, the borole rings exhibit a higher degree of distortion, with an average dihedral angle of 8.9° and 14.7° among the nonplanar subset. The only exception to this trend is molecule 10, in which the borinine ring is more distorted than the borole ring.

Fig. 3 summarizes the multicenter index (MCI) and Table 2 lists the energy difference between the lowest-energy triplet state and the singlet ground state. Analysis of the MCI data reveals two general trends. First, the five-membered borole moiety retains its antiaromatic character within the boroloborinine framework in the singlet ground state, consistent with the findings of Ando *et al.*^34^ In many cases the MCI values of the 5-membered rings are even smaller than that of the free borole (0.36), indicating more pronounced antiaromaticity. In most cases, the six-membered borinine ring is slightly less antiaromatic than the adjacent borole ring according to the MCI values. The only notable exception is compound 8, which exhibits the highest singlet–triplet energy gap among the bicyclic boroloborinine derivatives at both M06-2X/def2-TZVPP and CCSD(T)/def2-SVP//CCSD/def2-SVP levels. Second, MCI values indicate that, in the triplet state, the borole part of the molecules generally loses its antiaromaticity and, in some cases, even becomes weakly aromatic. This is consistent with the analysis of the spin density in the triplet state (Fig. S4), which shows that the majority of the unpaired electrons are localized within the five-membered rings of the molecules. This localization is consistent with the pronounced change in the aromatic character observed for this ring. For nine molecules (7, 10, 12, 16, 18, 19, 20, 22, and 27), spin-density analysis indicates that the majority of the unpaired electrons in the triplet state are localized on the 6-membered ring. Consistently, the MCI values for these species show a more pronounced transition from antiaromaticity in the singlet state to aromaticity in the triplet state within the 6-membered rings, in agreement with Baird's rule. It is important to note, however, that because the unpaired electrons are not fully localized on a single ring, the resulting triplet-state aromaticity remains moderate and does not reach the level observed for monocyclic borole.

**Table 2:** Singlet-triplet energy gaps in kcal mol^−1^ for bicyclic molecules at four different computational levels (M06-2X/def2-TZVPP, CAM-B3LYP/def2-TZVPP, CCSD/def2-SVP, and CCSD(T)/def2-SVP)

Molecule	M06-2X	CAM-B3LYP	CCSD	CCSD(T)	Molecule	M06-2X	CAM-B3LYP	CCSD	CCSD(T)
1	10.62	7.3	12.4	14.4	15	11.64	8.6	13.9	16.5
2	17.34	13.4	19.6	21.4	16	17.22	6.2	11.8	13.8
3	14.70	11.0	14.9	16.8	17	4.91	2.8	8.2	8.4
4	9.91	6.4	12.2	14.1	18	15.67	14.2	26.0	22.8
5	12.66	9.6	13.8	16.3	19	14.32	11.5	22.5	21.4
6	9.32	6.1	11.9	13.8	20	15.64	13.8	24.3	23.4
7	22.01	17.9	24.9	25.3	21	10.81	7.5	17.1	16.3
8	24.30	17.0	23.7	25.2	22	10.37	8.8	17.9	17.1
9	6.25	4.3	9.8	10.4	23	14.99	12.1	22.1	20.5
10	21.69	13.9	20.9	23.0	24	14.84	10.4	- a	- a
11	11.67	7.9	14.3	16.7	25	14.19	12.5	19.4	22.3
12	14.85	11.4	19.7	19.5	26	4.06	1.8	7.5	7.7
13	18.12	14.3	20.5	21.4	27	11.78	9.1	20.8	19.9
14	19.73	11.0	17.4	11.1	28	11.06	8.7	15.7	17.2

a Optimization of the triplet structure failed.

Examination of the MCI values and S–T gaps suggests that MCI values alone are seemingly insufficient for quantitatively identifying compounds with large singlet–triplet energy gaps. For instance, compounds 7, 8, and 10 exhibit the highest S–T gaps, yet their MCI values are not significantly different from those of the other derivatives. Similarly, we did not find any meaningful correlation between the HOMO–LUMO gaps (Table S1) and the MCI values, Fig. S3. This behavior can be attributed to the inherent sensitivity of the MCI to molecular geometry rather than the HOMO–LUMO gap or the nature of the excited states. Indeed, nonplanarity, which is common among the studied molecules, strongly affects the MCI values and complicates the identification of their aromatic or antiaromatic character. For example, in the singlet electronic states, the MCI values of planar systems are smaller than those of nonplanar analogues, indicating a higher degree of antiaromaticity. This trend is especially pronounced for the MCI values of the borinine rings. It is also noteworthy that restricting the analysis to planar structures does not significantly improve the correlation coefficients, presumably because of the inherent lack of correlation between the MCI and the HOMO–LUMO or S–T gaps (Fig. S3). MCI analysis also reveals that, in some polyaza-derivatives of boroloborinine, the aromaticity of the borinine moiety remains largely unchanged upon singlet-to-triplet transition. This behavior is typically observed in molecules where the spin density on the 6-membered ring in the triplet state is relatively low.

**Fig. 4:**
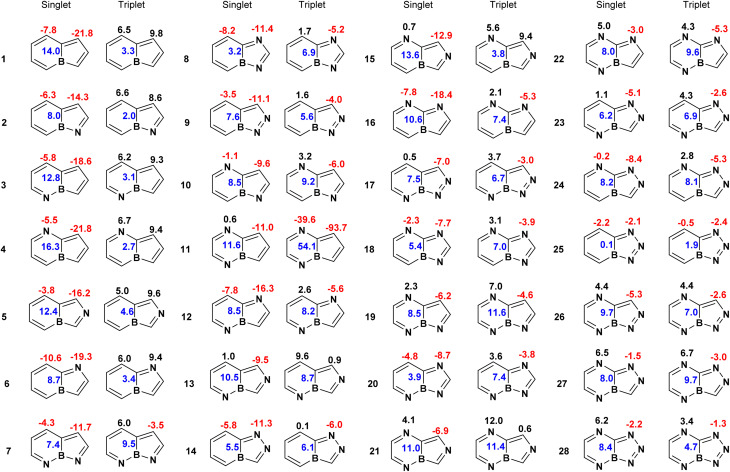
Magnetically induced current densities (in nA T^−1^) for boroloborinines. The magnitudes of diatropic and paratropic ring currents are indicated by black (positive) and red (negative) numbers, respectively, placed above each ring. The blue number at the center of each bicyclic system represents the current passing through the bond shared between the two rings. In molecules where both rings sustain the same type of ring current, *i.e.*, both diatropic or both paratropic, the blue value corresponds to the difference between the two ring currents. In contrast, when the two rings exhibit different types of current, *i.e.*, one diatropic and one paratropic, the blue value represents the sum of the two currents.

The extent and variation of aromaticity in the bicyclic boroloborinines were also evaluated using HOMA values and integrated magnetically induced current densities (MICD) (Fig. 4 and S1). Consistent with MCI analysis, the ring current data suggest that, with the exception of compounds 11 and 25, the borinine rings lose their antiaromatic character entirely in the triplet state. Among the planar systems, the borole rings in compounds 1 to 6 become aromatic in the triplet state, sustaining a relatively strong diatropic ring current. However, in the remaining planar molecules, the borole ring does not lose its paratropic character. The parent boroloborinine exhibits stronger antiaromaticity in its singlet ground state than its aza-substituted derivatives and becomes aromatic in the lowest-energy triplet state. In nonplanar molecules, both singlet-state antiaromaticity and triplet-state aromaticity are significantly reduced compared to their planar counterparts. This attenuation likely arises from diminished overlap between the occupied and virtual orbitals, responsible for defining the strength of the ring current, as a consequence of molecular nonplanarity.^44,45^ Similar to the MCI results, we did not observe a general correlation between the strength of the ring current and the singlet–triplet energy gap. The correlation between the ring current and the HOMO–LUMO gaps is not notable (*R*^2^ = 0.45), Fig. S3. A comparable lack of correlation was previously noted in our earlier study on various isomers of C_6_H_6_.^46^

However, focusing on the planar systems reveals an interesting trend. The singlet–triplet gap is larger in molecules where the borole ring exhibits weaker paratropicity in the singlet state and only slightly paratropic character in the triplet state. These features qualitatively indicate a reduction in antiaromaticity in the singlet state and a suppression of aromatic character in the triplet state. Indeed, a moderate correlation is observed between the paratropic current intensity of the borole rings in the singlet state and the corresponding S–T gaps (*R*^2^ = 0.75), as well as the HOMO–LUMO gaps (*R*^2^ = 0.82), among planar derivatives which also have a S–T gap larger than one-third of an electron volts (∼9 kcal mol^−1^), Fig. S3. The current intensities in the borole rings of molecules 7, 8, and 14, which have the largest S–T gaps among the planar systems, are −11.7, −11.4, and −11.3 nA T^−1^, respectively. The absolute values of these ring currents are notably smaller than those computed in molecules 1 to 6, which range from −21.8 to −14.3 nA T^−1^. Nevertheless, if structural parameters are rather similar, a correlation between the MICD and the HOMO–LUMO gap and to some extent the S–T gaps is expected from the fact that the strengths of currents depend on the energy gap between the frontier molecular orbitals, Fig. S3.^44,45,47^

HOMA data (Fig. S1) suggest that, in the singlet ground states, the borinine rings in boroloborinine derivatives range from nonaromatic to antiaromatic, whereas the borole rings consistently exhibit antiaromatic character. Similar to the trends observed in ring current and MCI analyses, the HOMA values indicate a transition from antiaromaticity in the singlet state to aromaticity in the triplet state for the parent boroloborinine, its planar monoaza derivatives, and molecules 7 and 25. However, HOMA results suggest that the remaining planar molecules retain their antiaromatic character even in the triplet state. Notably, in several cases, HOMA values indicate increased antiaromaticity in the triplet state compared to the singlet state. This unexpected trend may arise from inadequate parametrization, likely because of the lack of an appropriate reference framework for assessing aromaticity in triplet states. Overall, among the three classes of aromaticity indices examined, HOMA appears to be the least reliable for evaluating aromaticity in this context.

### Do nitrogen heteroatoms improve photochemical stability?

To conclude the observed trends, nitrogen heteroatoms depending on their relative positions and numbers can both decrease or increase the HOMO–LUMO and S–T energy gaps. Aromaticity indices can offer valuable insights when comparing structurally similar molecules; however, as structural diversity increases, identifying consistent trends becomes increasingly challenging. This is due to the multitude of factors influencing both the singlet–triplet (S–T) energy gaps and the (anti)aromatic character of these states. Ottosson and colleagues elucidated several key parameters that govern S–T gaps and aromaticity in heterocyclic species in their vertical excited states.^48,49^ However, to the best of our knowledge, analogous rules have not yet been established for relaxed triplet states in polyheterocyclic systems.

Nonetheless, despite the absence of generalized rules, our computational results indicate that the largest singlet–triplet (S–T) energy gaps among the studied molecules occur in the diaza derivatives of boroloborinine. As previously discussed, molecules 7, 8, 10, and 14 exhibit the largest S–T gaps, along with HOMO–LUMO gaps exceeding 5 eV, at the M06-2X computational level. Calculations using the CAM-B3LYP functional likewise identify these molecules as those with the largest S–T gaps. Single-point CCSD(T) computations also predict large S–T separations for these systems, although they indicate that a few other species may exhibit slightly higher values. Nevertheless, because the CCSD(T) S–T gaps are obtained from single-point energy calculations, we rely primarily on the DFT results for the subsequent stages of our analysis. Relative to the parent boroloborinine molecule, the substitution of CH groups with their electronic analog, nitrogen, increases the HOMO–LUMO gap by approximately 1 eV and doubles the S–T gap. These four molecules, along with boroloborinine, were selected for the next phase of our investigation.

### How does benzannulation improve photostability?

In a recent study, we demonstrated that benzannulation can increase the HOMO–LUMO gap of PAHs containing indoloindolizine units when it introduces Clar's sextets into the molecular framework. PAHs featuring Clar's sextets exhibit larger HOMO–LUMO gaps compared to analogs of similar or even smaller size that lack such aromatic stabilization.^35^

In the current work, benzannulation of boroloborinines yields a diverse set of molecules with a wide range of HOMO–LUMO and singlet–triplet energy gaps (Table 3 and S1). Among the 30 benzannulated structures that were optimized without ring opening, some exhibit triplet ground states, *i.e.*, negative S–T gaps (9 at M06-2X/def2-TZVPP, 12 at CAM-B3LYP/def2-TZVPP, and 5 at CCSD(T)/def2-SVP//M06-2X/def2-TZVPP levels of theory), whereas others show small S–T gaps (<10 kcal mol^−1^). Notably, all of the less stable species arise from benzannulation at positions 1, 2 or 6, 7, *i.e.*, sites that do not introduce Clar's sextets into the framework. For example, molecule 50, a tricyclic system benzannulated at positions 1 ,2, exhibits the lowest-energy triplet state among the studied species, lying approximately 15 kcal mol^−1^ below its singlet state according to DFT calculations. In contrast, benzannulation at positions 2, 3; 5, 6; or 7, 8 leads to the formation of Clar's sextets. These structures consistently display larger HOMO–LUMO and S–T gaps, reflecting the stabilizing influence of aromatic benzene rings incorporated into the molecular framework. The highest S–T gaps, confirmed by both DFT and CC computations, are found in molecules 38, 41, 42, 44, 47, and 51, which feature benzannulation at the aforementioned positions.

**Table 3:** Singlet–triplet energy gaps in kcal mol^−1^ for polycyclic molecules at three different computational levels (M06-2X/def2-TZVPP, CAM-B3LYP/def2-TZVPP, and CCSD/def2-SVP)

Molecule	M06-2X	CAM-B3LYP	CCSD	CCSD(T)	Molecule	M06-2X	CAM-B3LYP	CCSD	CCSD(T)
29	−7.6	−10.6	−5.8	−2.6	44	35.8	29.0	42.8	42.0
30	22.7	19.5	25.4	27.5	45	−7.3	−9.9	−8.9	−0.2
31	16.1	12.7	18.6	20.3	46	4.3	0.1	7.2	10.9
32	−0.3	−3.6	0.6	4.5	47	28.3	19.0	39.1	33.9
33	12.8	9.2	15.8	17.4	48	3.2	−6.5	−21.0	16.7
34	−4.2	−7.9	0.6	3.1	49	3.7	−0.8	6.7	9.0
35	2.9	−0.1	2.8	12.5	50	−13.9	−16.4	−13.4	−2.8
36	−7.2	−10.7	−2.8	−0.3	51	31.7	29.2	41.2	40.0
37	−8.9	−11.6	−7.0	0.3	52	10.5	6.0	11.7	15.5
38	29.2	25.8	32.6	34.6	53	7.4	2.2	9.0	11.0
39	8.1	4.8	10.9	15.1	54	−9.1	−12.8	−10.6	3.7
40	25.2	21.8	29.1	31.1	55	24.1	17.4	29.4	27.9
41	30.2	26.5	32.5	34.4	56	−6.4	−9.6	−5.2	4.7
42	32.4	26.6	42.3	41.1	57	16.5	13.2	20.7	22.5
43	27.3	22.7	29.6	31.1	58	21.3	18.0	23.8	25.7

Among the two pentacyclic systems derived from the parent boroloborinine, the site of benzannulation exerts a pronounced effect on the electronic structure. Molecule 37, benzannulated at positions 1, 2, possesses a triplet ground state approximately 10 kcal mol^−1^ more stable than its singlet (7 kcal mol^−1^ at the CCSD level but the singlet and triplet states seem to be degenerate at the CCSD(T) level). In contrast, molecule 41, benzannulated at positions 2, 3; 5, 6; and 7, 8, where Clar's sextets are formed, exhibits a stabilized singlet ground state. Similarly, the tetracyclic molecule 44, benzannulated at positions 5, 6 and 7, 8, shows the largest S–T gap among all tetracyclic systems, whereas its analogue 54, benzannulated at positions 1, 2 and 6, 7, possesses the most negative S–T gap, corresponding to a triplet ground state. Furthermore, molecule 50, a tricyclic species, exhibits the lowest-energy triplet state across all computational levels examined.

In summary, as observed previously in indoloindolizines, the position of benzannulation in boroloborinines has a more profound impact on both the HOMO–LUMO and S–T gaps than the overall size of the molecule, *i.e.*, the number of fused rings. The formation of Clar's sextets correlates directly with increased electronic stability, as reflected by larger HOMO–LUMO and S–T gaps.

**Fig. 5:**
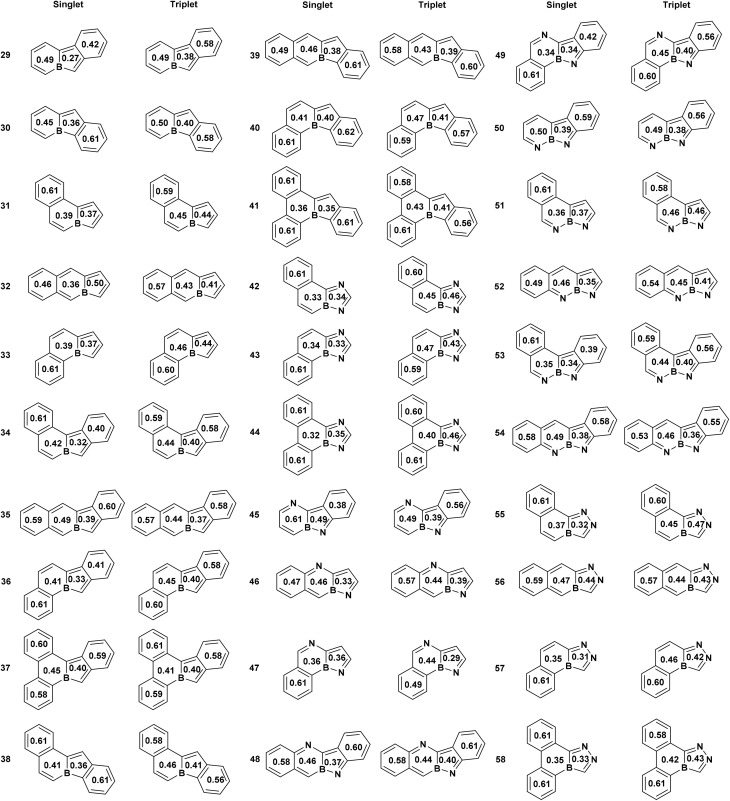
Structures of polybenzannulated boroloborinine. Each ring is annotated with its normalized multicenter index (MCI) value.

Multicenter index (MCI) analysis reveals consistent trends (Fig. 5). The core boroloborinine units in the benzannulated derivatives generally follow the behavior of their simpler bicyclic counterparts, with low MCI values in the singlet and higher values in the triplet states. Exceptions to this trend, where singlet MCI values are comparable to or greater than those in the triplet, are found in molecules with triplet ground states denoting potential triplet aromaticity. Clar's sextet rings consistently show the highest MCI values in the singlet state and typically retain those values in the triplet as well. This trend is consistent with the spin-density distributions: the boroloborinine moiety carries the highest spin density in the triplet state, whereas the benzene rings typically exhibit only minor spin localization. Among singlet structures, the terminal benzene rings at positions 1, 2 and 6, 7, which are not expected to host Clar's sextets, sometimes show comparably high MCI values, suggesting localized aromatic character despite the absence of a formal sextet. It should be noted that this phenomenon occurs only among molecules that have a ground triplet state and for which the singlet wavefunction at the DFT levels shows instability. Therefore, the high MCI values could be an artifact.

**Fig. 6:**
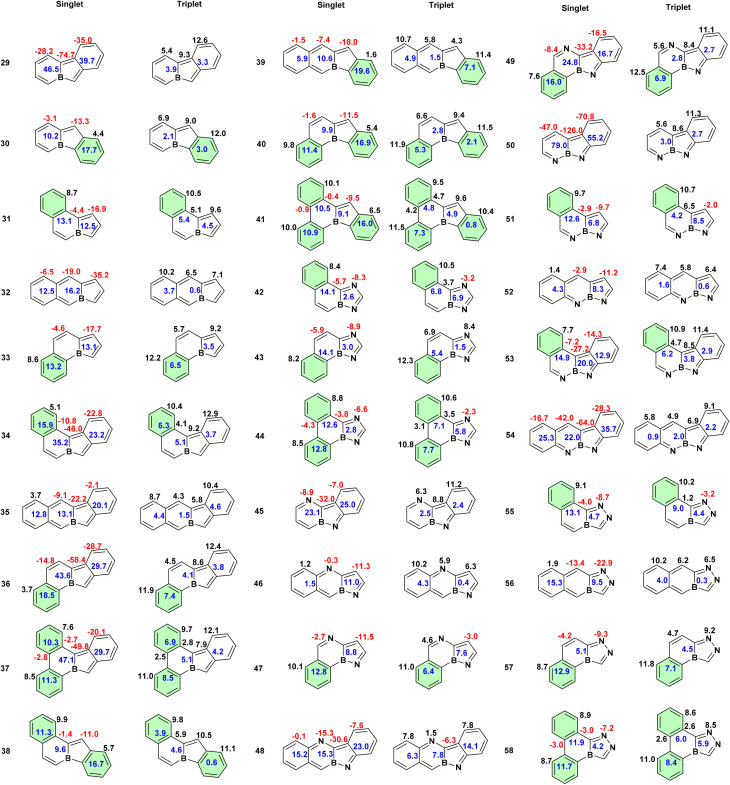
Magnetically induced current densities (nA/T). Diatropic (aromatic) and paratropic (antiaromatic) ring currents are denoted by black (positive) and red (negative) values, respectively. Blue numbers between rings indicate current through the shared bond that sums up the effect of neighboring rings. Rings shaded light green indicate local aromatic currents (Clar's sextets).

Ring current analysis (Fig. 6) broadly agrees with the MCI data but offers greater detail. All molecules with reversed S–T gaps exhibit strong paratropic ring currents in their singlet states, consistent with persistent antiaromaticity. However, the low-energy triplet species sustain diatropic ring currents consistent with triplet aromaticity. Rings featuring Clar's sextets show pronounced localized diatropic currents, regardless of the nature of the electronic (singlet or triplet) state. Terminal rings lacking Clar's sextets typically lack strong diatropic currents, though few terminal rings sustain weak local diatropic currents. The antiaromatic-to-aromatic transition upon excitation is clearly seen across the benzannulated series of the parent compound, boroloborinine. Among aza derivatives, certain systems exhibit weak paratropic currents in their triplet states, particularly those with large S–T gaps. This finding supports the central hypothesis of this work: high S–T gaps correlate with reduced singlet antiaromaticity and limited triplet aromaticity. Despite the fact that ring current is not a good measure of aromatic stabilization energy,^46,47,50–54^ it reflects the underlying electronic structure because the magnitude and the tropicity of the ring currents are defined by the molecular orbital symmetries and energy levels of the frontier orbitals.^44,45^

HOMA data also suggest a transition from antiaromaticity in the singlet to aromaticity, or at least reduced antiaromaticity, in the triplet states (Fig. S2). Among the polycyclic systems, HOMA shows better agreement with ring current and, in particular, MCI data compared to its performance in simpler bicyclic analogues (Fig. S1). This improved consistency likely stems from the fact that the polycyclic structures are generally more planar and less geometrically distorted than their bicyclic counterparts. Like MCI, HOMA highlights strong aromatic character in rings with Clar's sextets and, interestingly, also in some terminal rings without formal Clar structures. While not always aligned with ring current results, HOMA confirms the trend that high S–T gaps are most often observed in molecules with low antiaromaticity in the singlet and minimal aromatic stabilization in the triplet.

Before concluding this section, we must clarify that molecules with small singlet–triplet gaps may exhibit significant multireference character. This is reflected in some unstable DFT wavefunctions and elevated *T*_1_ diagnostics in the CC calculations (Tables S6–S8). However, because the primary objective of this work is to identify synthetically viable boroloborinine derivatives, a detailed discussion of these electronic-structure complications is deferred to the ESI, where additional data and commentary are provided.

### Proton affinity and stability in acidic environments

All nitrogen atoms in the studied systems possess a lone pair of electrons and can thus act as Lewis bases. Protonation of these nitrogen centers under acidic conditions may trigger undesired transformations, including spontaneous ring opening or activation of double bonds toward addition reactions. To evaluate the stability of these molecules in acidic environments, we computed their proton affinities (PAs) for the singlet ground-state structures identified by both DFT methods and CCSD. This dataset comprises 58 distinct protonation sites, summarized in Table 4. For comparison, the experimental and calculated (M06-2X/def2-TZVPP) gas-phase proton affinities of pyridine are 220.8 and 220.1 kcal mol^−1^.^55^

**Table 4:** Proton affinities (PA, kcal mol^−1^) of the examined molecules at various protonation sites computed at the M06-2X/def2-TZVPP level. Protonation site numbers follow the atom numbering of the boroloborinine framework shown in Fig. 1e

Molecule	Site	PA	Molecule	Site	PA	Molecule	Site	PA
7	3	228.7	17	5	221.0	24	1	218.2
	5	229.9	18	1	216.4		2	214.8
8	1	219.3		3	220.9		8	210.0
	3	225.2		8	210.3	27	1	213.6
9	2	210.5	19	3	224.6		2	213.9
	3	218.5		5	214.6		5	199.8
10	3	226.5		8	204.0		8	196.0
	8	216.8	20	1	213.5	42	1	224.7
11	5	224.6		3	221.8		3	234.2
	8	212.6		5	217.4	43	1	229.4
12	1	219.9	21	2	214.4		3	238.1
	5	223.3		5	217.1	44	1	230.1
13	2	217.5		8	206.2		3	238.1
	5	230.5	22	1	219.7	47	3	234.2
14	1	220.2		5	206.4		8	225.5
	2	219.0		8	202.6	51	3	236.4
16	1	224.2	23	1	214.2		5	232.9
	8	217.2		2	216.2	55	1	227.4
17	2	208.1		5	214.7		2	225.9
	3	216.1						

The proton affinities of the bicyclic species generally fall within ±10 kcal mol^−1^ of the pyridine reference value. In these systems, nitrogen atoms located closer to the electropositive boron center exhibit higher basicity due to charge transfer from boron to nitrogen. Consequently, nitrogen substitution at positions 3 and 5 of the boroloborinine scaffold is unfavorable in terms of proton affinity. Extension of the π-conjugated framework by ring fusion renders the molecules more electron-rich, increasing their proton affinities by approximately 10 kcal mol^−1^ relative to analogous nitrogen sites in the bicyclic species. To mitigate potential protonation and thereby improve stability in acidic media, it is therefore advisable to introduce bulky substituents on the benzene rings adjacent to the nitrogen atoms to sterically hinder proton access.

## Conclusions

In this study, we have explored the stabilization strategies for boroloborinines, a class of almost forgotten antiaromatic bicyclic boron heterocycles, using nitrogen substitution and benzannulation. Our DFT and CCSD-based screening demonstrates that the electronic properties, particularly the HOMO–LUMO and singlet–triplet (S–T) energy gaps, can be significantly modulated through structural modifications. Substitution with one or two nitrogen atoms at positions 1, 2, or 8 can increase both the HOMO–LUMO and S–T gaps. However, introducing a greater number of nitrogen heteroatoms has an adverse effect, often leading to structural distortion, σ–π mixing, and loss of aromatic or antiaromatic character.

Benzannulation at positions that promote the formation of Clar's sextets consistently enhances both HOMO–LUMO and S–T gaps, stabilizing the singlet ground state and suppressing unwanted triplet aromaticity. In contrast, expansion of the molecular framework without the formation of Clar's sextets lowers the energy of the triplet state, often below that of the singlet. Among all molecules studied, the most stable and photochemically robust structures are those exhibiting reduced antiaromaticity in the singlet state and diminished aromaticity in the triplet state. This can be achieved through a combination of targeted benzannulation and nitrogen incorporation in the borole moiety of the molecule. Aromaticity indices (MCI, ring currents, and HOMA) generally support these observations, with MCI and ring current analyses providing more reliable insights than HOMA, particularly in non-planar systems. Ring current analysis, in particular, reflects changes in the HOMO–LUMO and S–T gaps, as the strength and direction (tropicity) of the current are directly related to molecular orbital symmetries and the energies of the frontier orbitals, as shown by Fowler and Steiner.^44,45^ These findings not only elucidate the electronic structure–stability relationships in boroloborinines but also provide guiding principles for the design of antiaromatic materials with tunable photophysical properties. The conceptual parallels between these systems and recently studied indoloindolizines suggest broader implications for the rational design of antiaromatic dyes in near-infrared emissive and photoresponsive materials.

To complement photostability analysis, we evaluated the proton affinity (PA) of all singlet-stable species to estimate their resistance to acid-induced degradation. The computed PAs (typically within ±10 kcal mol^−1^ of pyridine) indicate that most bicyclic boroloborinines possess moderate Lewis basicity, with higher values observed for nitrogen atoms adjacent to electropositive boron centers. In particular, nitrogen substitution at positions 3 and 5, adjacent to the boron atom, was found to be unfavorable from the perspective of proton affinity. Similarly, π-extension *via* benzannulation increases the PA by approximately 10 kcal mol^−1^, reflecting greater electron richness. These results suggest that the most photochemically and chemically robust derivatives balance modest proton affinity, sufficient to resist protonation-induced decomposition, with large S–T gaps that suppress photoinduced reactivity. To further enhance acid stability, steric protection near basic nitrogen sites through bulky substituents on benzene rings is recommended.

## Author contributions

Conceptualization: CFN; methodology: CFN, MYM; investigation: MYM, MSh, MS; visualization: MYM, MSh; funding acquisition: CFN; project administration: CFN; supervision: CFN; analysis: MYM, MSh, MS; writing – initial reports: MYM, MSh; writing – review & editing: MYM, MSh, MS, CFN.

## Conflicts of interest

The authors declare no competing financial interest.

## Supplementary Material

SC-017-D5SC05880E-s001

## Data Availability

All data supporting the findings of this study are presented within the article and the supplementary information (SI). Supplementary information is available. See DOI: https://doi.org/10.1039/d5sc05880e.
