# Supplementary material for: Taming boroloborinines: toward photostable polycyclic antiaromatic hydrocarbons
Source: Chem Sci. 2026 Jan 20;17(14):6983–94. doi: 10.1039/d5sc05880e (PMC12908706; doi:10.1039/d5sc05880e)
Supplement: SC-017-D5SC05880E-s001 [file SC-017-D5SC05880E-s001.pdf]

Supplementary Information (SI) for Chemical Science.
This journal is © The Royal Society of Chemistry 2026

**Supporting Information**

This version of the Supplementary Information, published 10/02/26, replaces the version published previously on 20/01/26.

# Taming Boroloborinines: Toward Photostable Polycyclic Antiaromatic Hydrocarbons

Muhammad Yasir Mehboob[a†], Minu Sheeja[a†], Mahdi Sasar[a], Cina Foroutan-Nejad[a*]

a. Institute of Organic Chemistry, Polish Academy of Sciences, Kasprzaka 44/52, 01-224, Warsaw, Poland.

Corresponding Author's Email: cina.foroutan-nejad@icho.edu.pl

[†] These authors contributed equally to this work.

# Supporting Information

**Table of Contents**

**Computational Methods**……………………………………………………………………page S3

**Table S1**………………………………………………………………………………page S5

**Table S2**………………………………………………………………………………..page S6

**Table S3-S5**…………………………………………………………………..……………page S7

**Table S7**………………………………………………………………………………..page S8

**Figure S1**……………………………………..…………………………………………page S8

**Figure S2**……………………………………..…………………………………………page S9

**Figure S3**……………………………………..…………………………………………pages S10-S40

**Figure S4**……………………………………..…………………………………………pages S41

**Figure S5**……………………………………..…………………………………………pages S42

**Optimized molecular coordinates**……………………………………………….............pages S43-S63

## Supporting Information

### Computational detail

All the studied geometries at ground singlet and lowest-energy triplet electronic states have been optimized using the Gaussian 16 program[1]. Optimization was performed with the functional M06-2X[2] and the def2-TZVPP[3,4] basis set. M06-2X is a reliable and balanced functional that provides a realistic description of π-systems and avoids artificial stabilization. All optimized geometries are true local minima based on the eigenvalues of the Hessian matrix. To verify the reliability of the M06-2X functional, all structures were also optimized at the CAM-B3LYP[5]/def2-TZVPP level, and single-point CCSD/def2-SVP and CCSD(T)/def2-SVP calculations were performed to assess the singlet-triplet gaps. Protonation energies were computed using the M06-2X/def2-TZVPP method. GIAO NMR calculations were performed at the same level of theory, and WFX files were generated and saved directly from Gaussian. The wavefunctions were analyzed with the AIMAll[6] program. The ring current of all studied molecules was analyzed using AIMAll, where the flux of current density was passed through the interatomic surface after applying a magnetic field perpendicular to the ring plane. To further evaluate the aromaticity of the reported molecules, HOMA and MCI analyses were conducted using the FCHK file (directly saved from Gaussian) via the multiWFN program.[7,8] Time-dependent DFT calculations with the Tamm-Dancoff approximation [9] (TDA), TD-DFT were used to compute the excited state absorption values and oscillating strengths, as listed in Table S1. The dihedral angles of the molecules are reported in Table S2, explaining the reason for the high singlet-triplet gaps.

### References


1 M. J. Frisch, G. W. Trucks, H. B. Schlegel, G. E. Scuseria, M. A. Robb, J. R. Cheeseman, G. Scalmani, V. Barone, G. A. Petersson, H. Nakatsuji, X. Li, M. Caricato, A. V. Marenich, J. Bloino, B. G. Janesko, R. Gomperts, B. Mennucci, H. P. Hratchian, J. V. Ortiz, A. F. Izmaylov, J. L. Sonnenberg, D. Williams-Young, F. Ding, F. Lipparini, F. Egidi, J. Goings, B. Peng, A. Petrone, T. Henderson, D. Ranasinghe, V. G. Zakrzewski, J. Gao, N. Rega, G. Zheng, W. Liang, M. Hada, M. Ehara, K. Toyota, R. Fukuda, J. Hasegawa, M. Ishida, T. Nakajima, Y. Honda, O. Kitao, H. Nakai, T. Vreven, K. Throssell, J. A. Montgomery, Jr., J. E. Peralta, F. Ogliaro, M. J. Bearpark, J. J. Heyd, E. N. Brothers, K. N. Kudin, V. N. Staroverov, T. A. Keith, R. Kobayashi, j. Normand, K. Raghavachari, A. P. Rendell, J. C. Burant, S. S. Iyengar, J. Tomasi, M. Cossi, J. M. Millam, M. Klene, C. Adamo, R. Cammi, J. W. Ochterski, R. L. Martin, K. Morokuma, O. Farkas, J. B. Foresman and D. J. Fox, Gaussian 16 (versions ES64L-G16RevB) Gaussian, Inc., Wallingford CT 2016.
2 Y. Zhao and D. G. Truhlar, *Theor. Chem. Acc.*, 2008, **120**, 215–241.
3 F. Weigend, *Phys. Chem. Chem. Phys.*, 2006, **8**, 1057–1065.
4 F. Weigend and R. Ahlrichs, *Phys. Chem. Chem. Phys.*, 2005, **7**, 3297–3305.
5 T. Yanai, D. P. Tew and N. C. Handy, *Chem. Phys. Lett.*, 2004, **393**, 51–57.

6 T. A. Keith Gristmill Software, Overland Park KS, USA, 2019 (aim.tkgristmill.com), AIMAll 19.10.12 Gristmill Software, Overland Park KS, USA, 2019 (aim.tkgristmill.com) (version 19.10.12) Gristmill Software, Overland Park KS, USA 2019.
7 T. Lu and F. Chen, *J. Comput. Chem.*, 2012, **33**, 580–592.
8 T. Lu, *J. Chem. Phys.*, 2024, **161**, 082503.
9 S. Hirata and M. Head-Gordon, *Chem. Phys. Lett.*, 1999, **314**, 291–299.

## Supporting Information

**Table S1**: Computed HOMO-LUMO gaps (H-L) wavelength ($\lambda_{max}$) and oscillating strength (*f*) of all the studied molecules at M06-2X/def2-TZVPP level of theory.

| Molecules | HL(eV) | $\lambda_{max}$ (nm) | *f* | Molecules | HL(eV) | $\lambda_{max}$ (nm) | *f* |
|---|---|---|---|---|---|---|---|
| 1 | 4.65 | 207.75 | 0.31 | 30 | 4.76 | 234.65 | 0.56 |
| 2 | 5.03 | 202.18 | 0.23 | 31 | 4.75 | 201.47 | 0.29 |
| 3 | 4.8 | 201.9 | 0.31 | 32 | 3.63 | 250.02 | 0.21 |
| 4 | 4.76 | 207.85 | 0.29 | 33 | 4.6 | 242.36 | 0.48 |
| 5 | 4.86 | 200.83 | 0.20 | 34 | 3.62 | 256.56 | 0.31 |
| 6 | 4.84 | 202.18 | 0.23 | 35 | 3.45 | 245.14 | 1.67 |
| 7 | 5.35 | 256.43 | 0.04 | 36 | 3.43 | 268.71 | 0.62 |
| 8 | 5.26 | 208.04 | 0.12 | 37 | 3.18 | 266.71 | 0.79 |
| 9 | 4.84 | 196.84 | 0.15 | 38 | 4.86 | 236.44 | 1.13 |
| 10 | 5.58 | 259.96 | 0.07 | 39 | 3.78 | 240.28 | 1.16 |
| 11 | 5.11 | 204.89 | 0.18 | 40 | 4.72 | 252.19 | 1.06 |
| 12 | 5.06 | 279.06 | 0.05 | 41 | 4.84 | 244.9 | 1.13 |
| 13 | 5.41 | 223.93 | 0.14 | 42 | 5.29 | 223.4 | 0.46 |
| 14 | 5.17 | 193.92 | 0.27 | 43 | 5.13 | 217.36 | 0.51 |
| 15 | 5.26 | 221.04 | 0.13 | 44 | 5.16 | 237.31 | 0.19 |
| 16 | 5.02 | 200.47 | 0.23 | 45 | 3.67 | 266.39 | 0.25 |
| 17 | 4.99 | 211.07 | 0.07 | 46 | 4.51 | 261.99 | 0.17 |
| 18 | 5.59 | 258.02 | 0.06 | 47 | 5.27 | 226.43 | 0.47 |
| 19 | 5.36 | 197.49 | 0.21 | 48 | 3.19 | 300.36 | 1.00 |
| 20 | 5.24 | 253.43 | 0.03 | 49 | 3.91 | 251.7 | 0.52 |
| 21 | 5.38 | 198.23 | 0.15 | 50 | 3.05 | 271.07 | 0.50 |
| 22 | 5.26 | 200.61 | 0.16 | 51 | 5.3 | 242.8 | 0.22 |
| 23 | 5.56 | 199.58 | 0.09 | 52 | 4.47 | 245.89 | 0.44 |
| 24 | 5.58 | 216.42 | 0.07 | 53 | 4.01 | 239.35 | 0.13 |
| 25 | 4.83 | 248.65 | 0.14 | 54 | 2.76 | 298.03 | 0.69 |
| 26 | 5.32 | 214.68 | 0.69 | 55 | 5.19 | 228.51 | 0.24 |
| 27 | 5.5 | 207.05 | 0.08 | 56 | 3.79 | 432.44 | 0.24 |
| 28 | 5.57 | 227.82 | 0.05 | 57 | 4.98 | 218.41 | 0.46 |
| 29 | 3.43 | 220.8 | 0.87 | 58 | 5.03 | 235.41 | 0.32 |

**Table S2**: Dihedral angles of all the studied molecules by considering both parent rings

| Molecules | φ along borinine | φ along borole | Molecules | φ along borinine | φ along borole |
|---|---|---|---|---|---|
| 1 | -0.018 | 0.018 | 30 | -0.014 | 0.018 |
| 2 | -0.010 | -0.001 | 31 | -0.005 | 0.006 |
| 3 | 0.004 | 0.002 | 32 | -0.033 | 0.047 |
| 4 | -0.001 | -0.004 | 33 | 0.037 | -0.040 |
| 5 | -0.002 | -0.012 | 34 | -8.262 | -10.710 |
| 6 | -0.002 | -0.005 | 35 | 0.053 | 0.018 |
| 7 | 0.032 | -3.643 | 36 | 0.009 | -0.008 |
| 8 | 0.013 | -0.012 | 37 | -11.767 | -11.821 |
| 9 | -0.006 | 0.007 | 38 | 0.003 | 0.050 |
| 10 | 19.647 | 9.846 | 39 | 0.015 | -0.009 |
| 11 | 2.988 | -15.461 | 40 | 0.013 | -0.005 |
| 12 | -0.001 | -0.053 | 41 | -0.014 | -0.030 |
| 13 | -3.367 | 14.216 | 42 | 0.001 | -0.005 |
| 14 | 0.003 | 0.000 | 43 | -0.028 | 0.044 |
| 15 | -2.486 | -15.898 | 44 | -0.014 | 0.017 |
| 16 | 0.005 | -0.002 | 45 | -2.396 | -11.193 |
| 17 | -0.296 | 14.793 | 46 | 10.983 | 23.986 |
| 18 | -12.266 | -16.803 | 47 | 0.053 | 0.023 |
| 19 | -2.916 | -19.939 | 48 | -0.020 | -0.065 |
| 20 | -0.912 | 6.093 | 49 | -0.025 | -0.039 |
| 21 | 4.051 | -19.092 | 50 | -0.022 | -0.029 |
| 22 | 0.666 | 15.886 | 51 | -0.014 | 0.022 |
| 23 | -4.166 | 16.373 | 52 | 2.345 | 16.060 |
| 24 | -6.354 | -17.741 | 53 | 1.192 | 2.181 |
| 25 | 0.002 | 0.017 | 54 | -0.022 | -0.010 |
| 26 | 0.906 | 22.003 | 55 | 0.008 | -0.014 |
| 27 | 0.729 | -20.260 | 56 | 11.191 | 12.746 |
| 28 | -4.685 | -21.790 | 57 | 0.013 | -0.024 |
| 29 | 0.028 | 0.003 | 58 | -0.005 | -0.001 |

**Table S3**. Singlet-triplet energy gaps in kcal/mol for bicyclic molecules at CCSD/def2-SVP, and CCSD(T)/def2SVP by using optimized geometries of CAM-B3LYP.

| Molecule | CCSD | CCSD(T) | Molecule | CCSD | CCSD(T) |
|---|---|---|---|---|---|
| **1** | 12.1 | 13.8 | **15** | 13.5 | 16.0 |
| **2** | 19.4 | 20.7 | **16** | 11.6 | 13.0 |
| **3** | 14.6 | 16.2 | **17** | 8.3 | 8.0 |
| **4** | 11.8 | 13.4 | **18** | 25.8 | 22.7 |
| **5** | 13.5 | 15.8 | **19** | 23.5 | 21.0 |
| **6** | 11.6 | 13.0 | **20** | 26.2 | 20.3 |
| **7** | 25.0 | 24.8 | **21** | 17.2 | 15.8 |
| **8** | 23.5 | 24.5 | **22** | 18.4 | 15.2 |
| **9** | 10.1 | 9.8 | **23** | 21.7 | 21.4 |
| **10** | 20.6 | 22.5 | **24** | 17.8 | 20.0 |
| **11** | 15.2 | 15.2 | **25** | 19.0 | 21.3 |
| **12** | 20.2 | 19.2 | **26** | 7.1 | 7.5 |
| **13** | 20.3 | 20.9 | **27** | 21.7 | 19.6 |
| **14** | - [a] | - [a] | **28** | 15.2 | 17.0 |

a- Triplet geometry contains spin contamination

**Table S4**. Singlet-triplet energy gaps in kcal/mol for polycyclic molecules at CCSD/def2-SVP, and CCSD(T)/def2-SVP by using optimized geometries of CAM-B3LYP.

| Molecule | CCSD | CCSD(T) | Molecule | CCSD | CCSD(T) |
|---|---|---|---|---|---|
| **29** | -6.2 | -2.9 | **44** | 34.9 | 35.9 |
| **30** | 25.3 | 27.3 | **45** | -8.7 | -0.3 |
| **31** | 18.5 | 20.1 | **46** | 7.3 | 10.8 |
| **32** | 0.2 | 4.2 | **47** | 25.8 | 27.3 |
| **33** | 15.6 | 17.2 | **48** | -3.8 | 6.2 |
| **34** | 0.3 | 2.8 | **49** | 6.5 | 8.8 |
| **35** | 2.6 | 12.1 | **50** | -13.5 | -3.3 |
| **36** | -3.1 | -0.6 | **51** | 40.1 | 39.2 |
| **37** | -7.1 | 0.2 | **52** | 11.6 | 15.3 |
| **38** | 32.5 | 34.4 | **53** | 8.8 | 10.9 |
| **39** | 10.6 | 14.7 | **54** | -10.8 | 3.1 |
| **40** | 28.9 | 30.8 | **55** | 24.0 | 25.8 |
| **41** | 32.6 | 34.4 | **56** | -5.3 | 4.6 |
| **42** | 33.9 | 34.7 | **57** | 20.6 | 22.3 |
| **43** | 29.4 | 31.0 | **58** | 23.8 | 25.6 |

**Table S5.** T1 diagnostic values at CCSD/def2-SVP for bicyclic molecules

| Molecule | T1 diagnostic (Singlet) | T1 diagnostic (Triplet) | Molecule | T1 diagnostic (Singlet) | T1 diagnostic (Triplet) |
|---|---|---|---|---|---|
| **1** | 0.013 | 0.038 | **15** | 0.016 | 0.039 |
| **2** | 0.014 | 0.039 | **16** | 0.018 | 0.039 |
| **3** | 0.015 | 0.036 | **17** | 0.019 | 0.032 |
| **4** | 0.013 | 0.038 | **18** | 0.022 | 0.033 |
| **5** | 0.017 | 0.038 | **19** | 0.022 | 0.058 |
| **6** | 0.015 | 0.039 | **20** | 0.019 | 0.052 |
| **7** | 0.015 | 0.046 | **21** | 0.024 | 0.059 |
| **8** | 0.017 | 0.042 | **22** | 0.028 | 0.059 |
| **9** | 0.014 | 0.039 | **23** | 0.024 | 0.047 |
| **10** | 0.017 | 0.039 | **24** | 0.018 | - [a] |
| **11** | 0.019 | 0.036 | **25** | 0.017 | 0.051 |
| **12** | 0.015 | 0.049 | **26** | 0.025 | 0.035 |
| **13** | 0.021 | 0.047 | **27** | 0.031 | 0.065 |
| **14** | 0.014 | 0.042 | **28** | 0.028 | 0.049 |

a- Triplet geometry contains spin contamination

**Table S6.** T1 diagnostic values at SP-CCSD/def2-SVP for polycyclic molecules

| Molecule | T1 diagnostic (Singlet) | T1 diagnostic (Triplet) | Molecule | T1 diagnostic (Singlet) | T1 diagnostic (Triplet) |
|---|---|---|---|---|---|
| **29** | 0.018 | 0.039 | **44** | 0.015 | 0.034 |
| **30** | 0.012 | 0.040 | **45** | 0.043 | 0.039 |
| **31** | 0.012 | 0.041 | **46** | 0.017 | 0.040 |
| **32** | 0.017 | 0.038 | **47** | 0.015 | 0.039 |
| **33** | 0.012 | 0.039 | **48** | 0.023 | 0.050 |
| **34** | 0.013 | 0.042 | **49** | 0.017 | 0.042 |
| **35** | 0.016 | 0.038 | **50** | 0.045 | 0.038 |
| **36** | 0.012 | 0.041 | **51** | 0.013 | 0.054 |
| **37** | 0.016 | 0.035 | **52** | 0.019 | 0.039 |
| **38** | 0.012 | 0.044 | **53** | 0.014 | 0.040 |
| **39** | 0.014 | 0.041 | **54** | 0.030 | 0.040 |
| **40** | 0.012 | 0.042 | **55** | 0.013 | 0.036 |
| **41** | 0.012 | 0.037 | **56** | 0.019 | 0.042 |
| **42** | 0.015 | 0.045 | **57** | 0.013 | 0.042 |
| **43** | 0.016 | 0.042 | **58** | 0.013 | 0.035 |

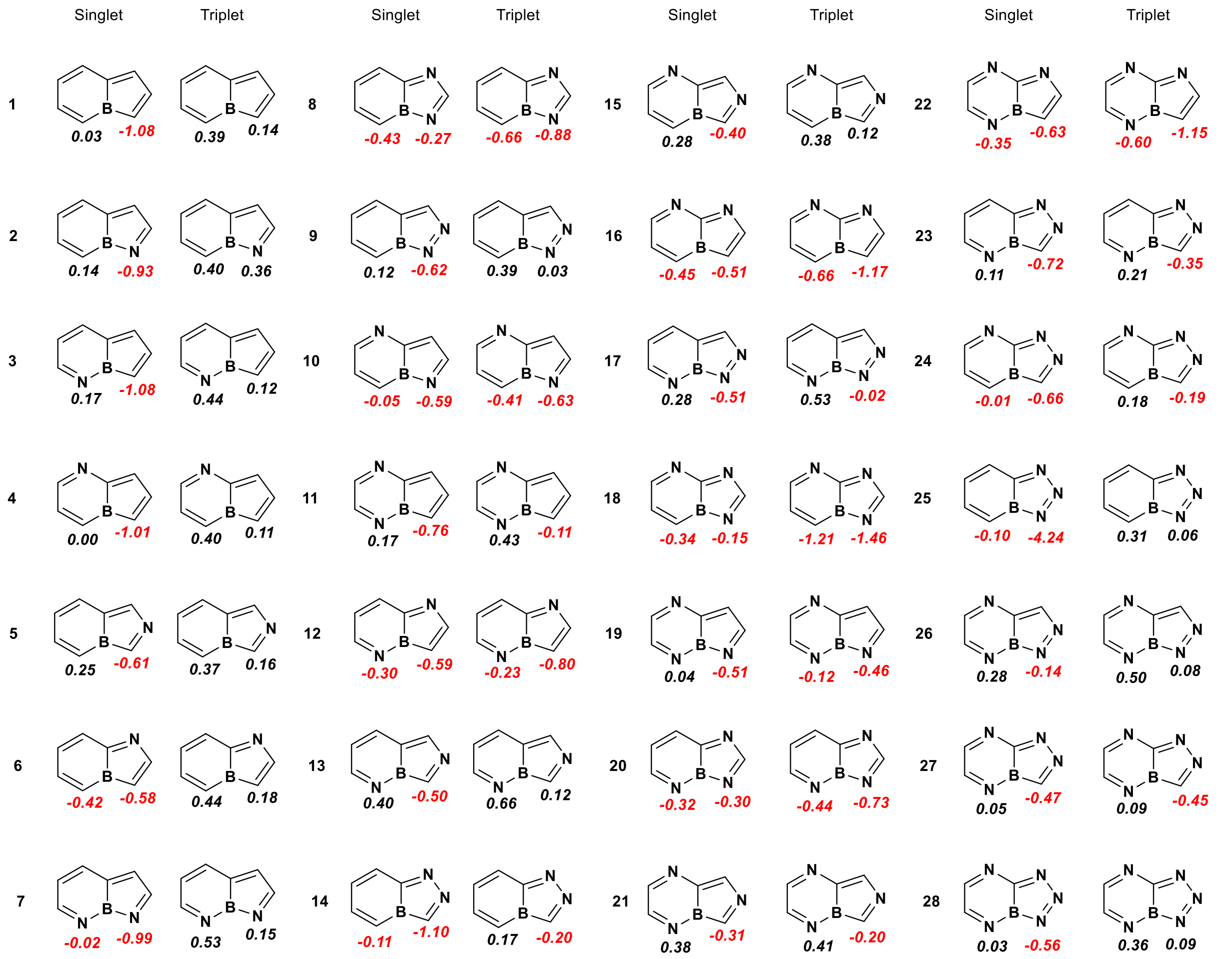


**Figure S1**. HOMA values of bicyclic molecules. Positive and negative values, indicating aromaticity and antiaromaticity, respectively, are listed in black and red fonts.

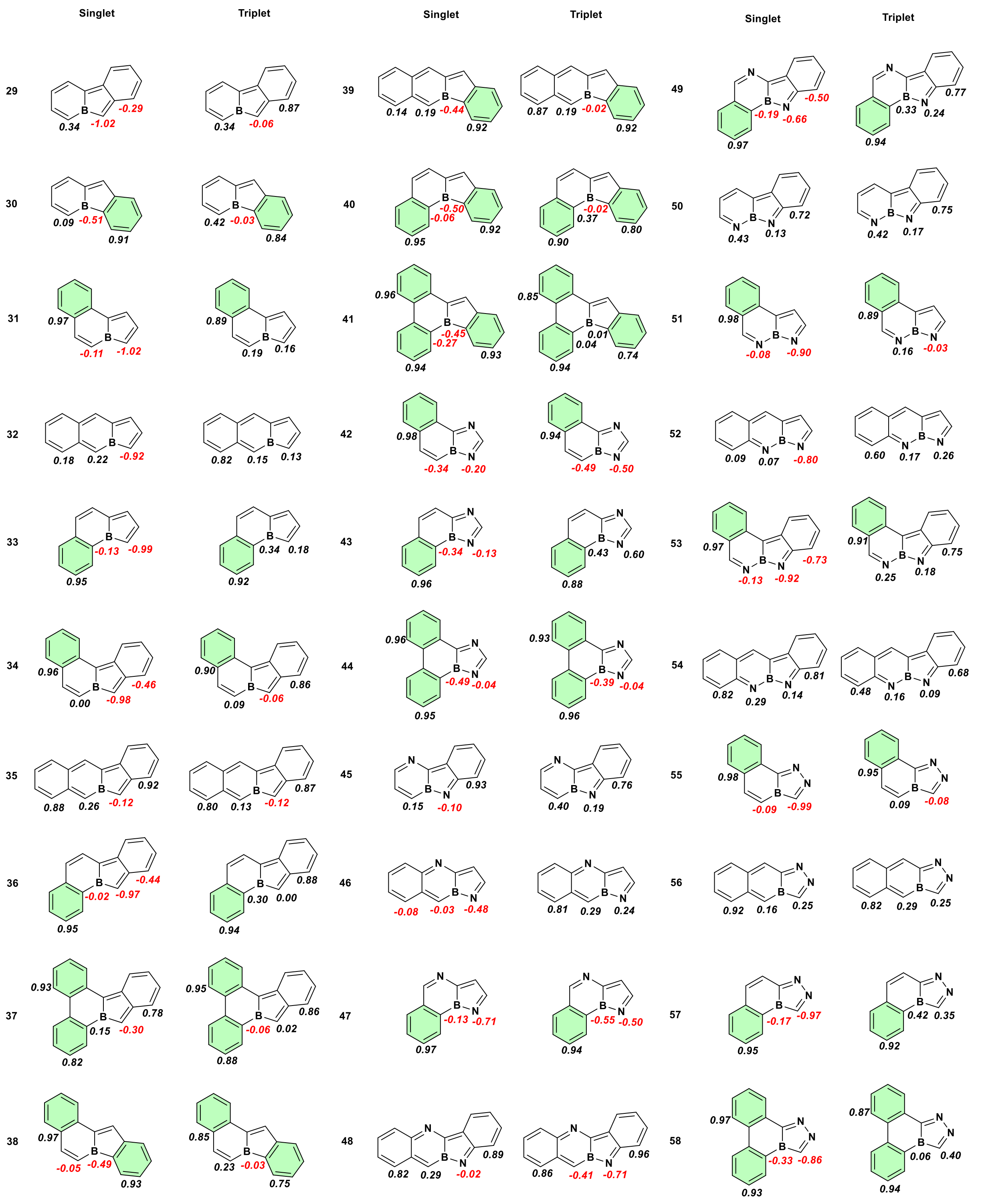


**Figure S2**. HOMA values of polycyclic molecules. Positive and negative values, indicating aromaticity and antiaromaticity, respectively, are listed in black and red fonts. Rings with a green filling are those that possess a Clar's sextet based on the ring current analysis.

**Figure S3**. Correlation coefficients between various aromaticity indicators and the HOMO-LUMO and singlet-triplet gaps:

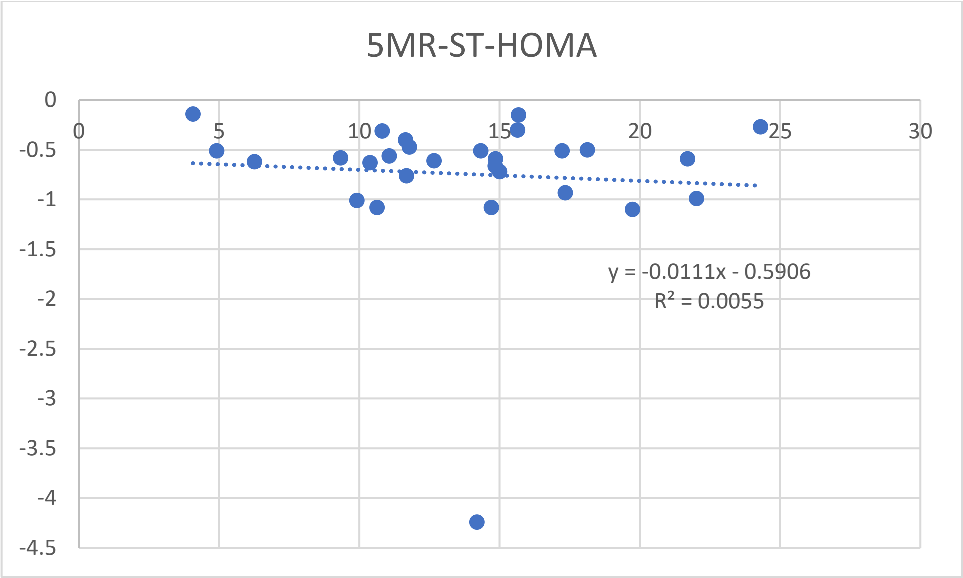


(a) Singlet-triplet gap versus HOMA values of the 5-membered rings among bicyclic species.

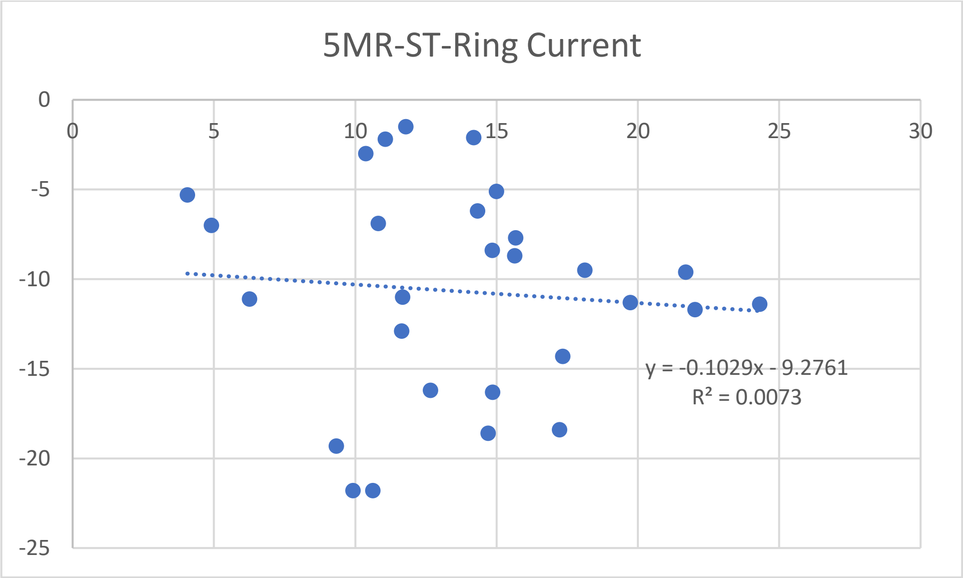


(b) Singlet-triplet gap versus ring current of the 5-membered rings among bicyclic species.

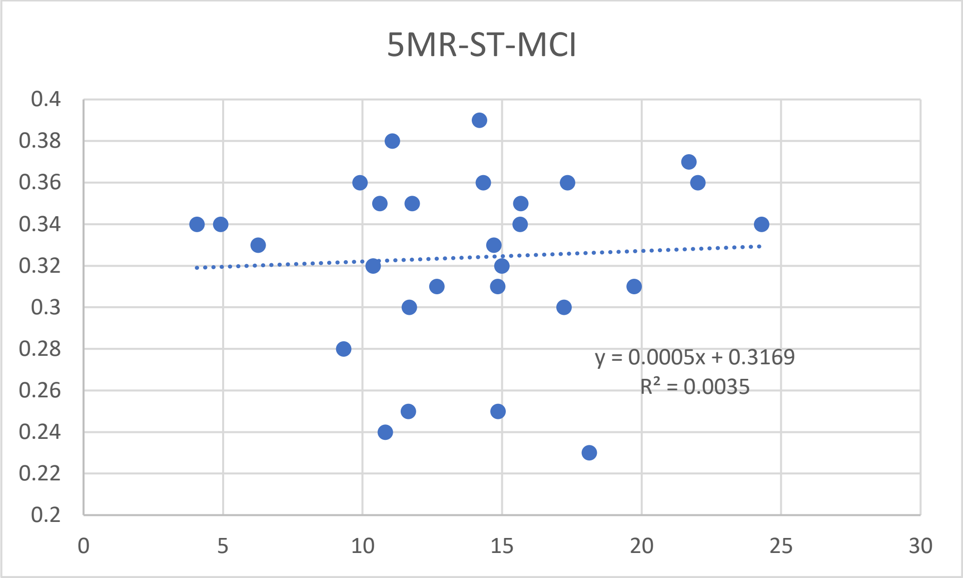


(c) Singlet-triplet gap versus MCI of the 5-membered rings among bicyclic species.

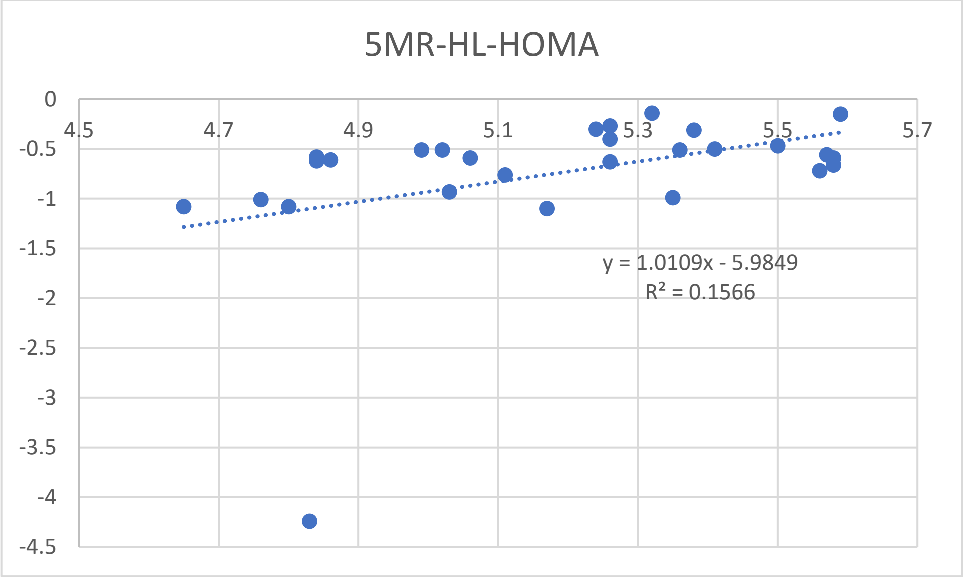


(d) HOMO-LUMO gap versus HOMA of the 5-membered rings among bicyclic species.

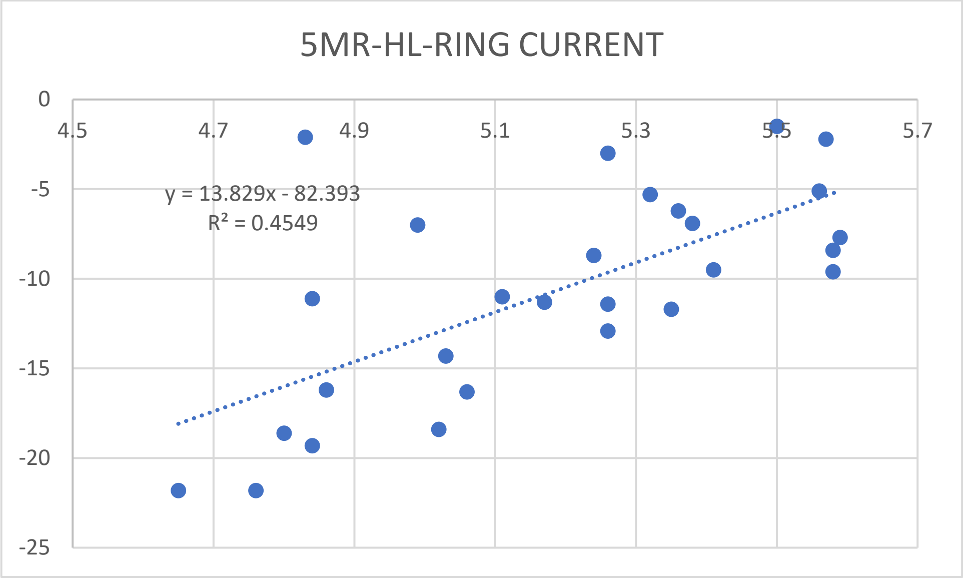


(e) HOMO-LUMO gap versus ring current of the 5-membered rings among bicyclic species.

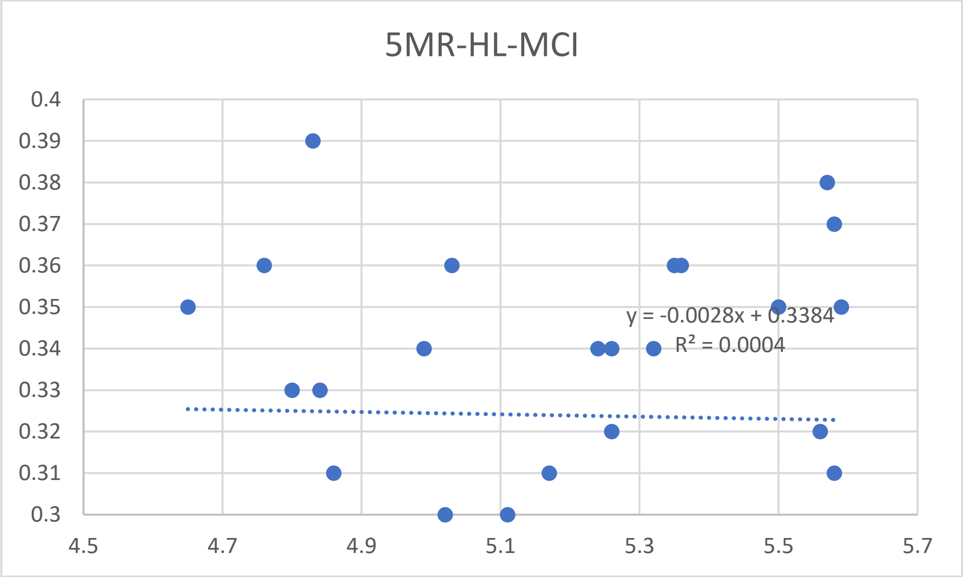


(f) HOMO-LUMO gap versus MCI of the 5-membered rings among bicyclic species.

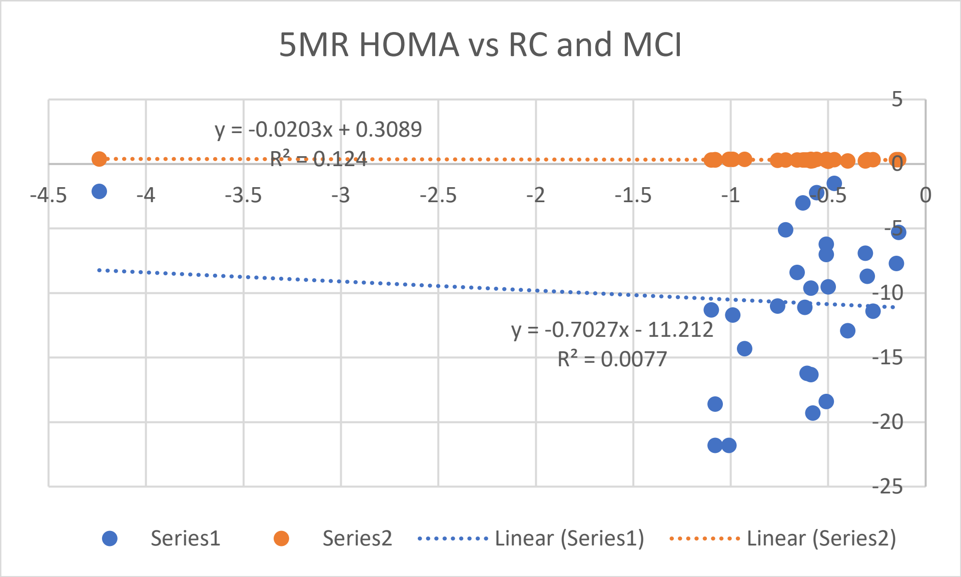


(g) HOMA vs ring current (Series 1) and MCI (Series 2) for the 5-membered rings among bicyclic species.

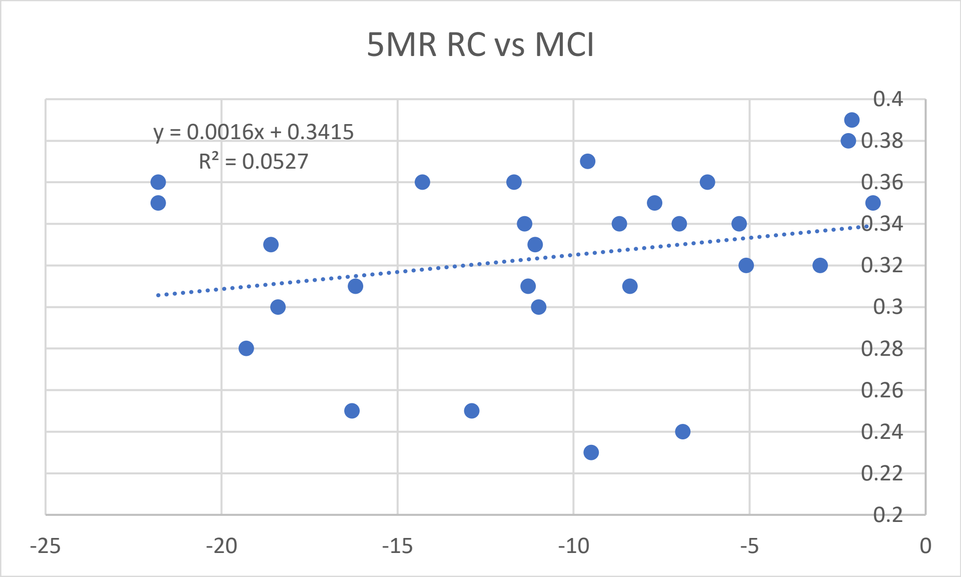


(h) Ring current versus MCI for the 5-membered rings among bicyclic species.

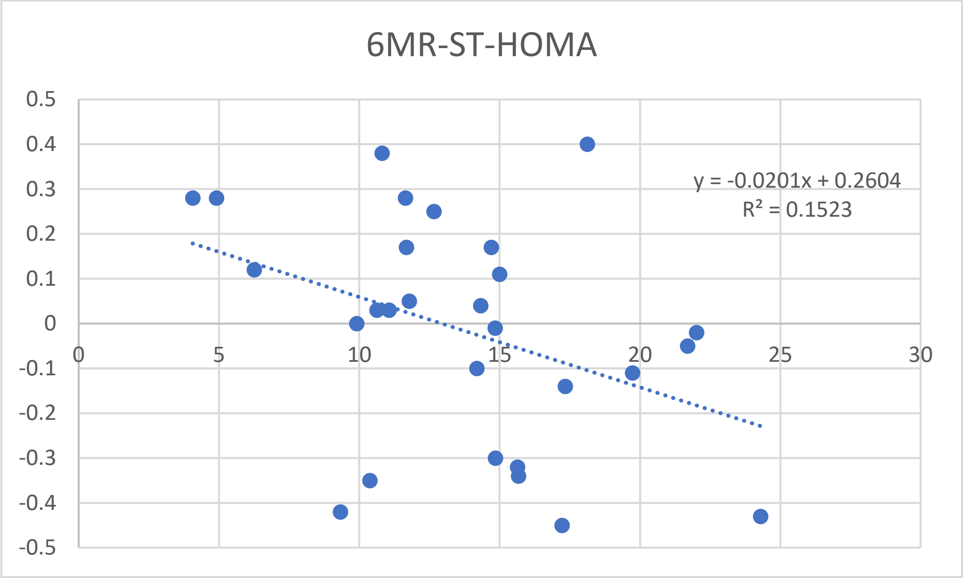


(i) Singlet-triplet gap versus HOMA values of the 6-membered rings among bicyclic species.

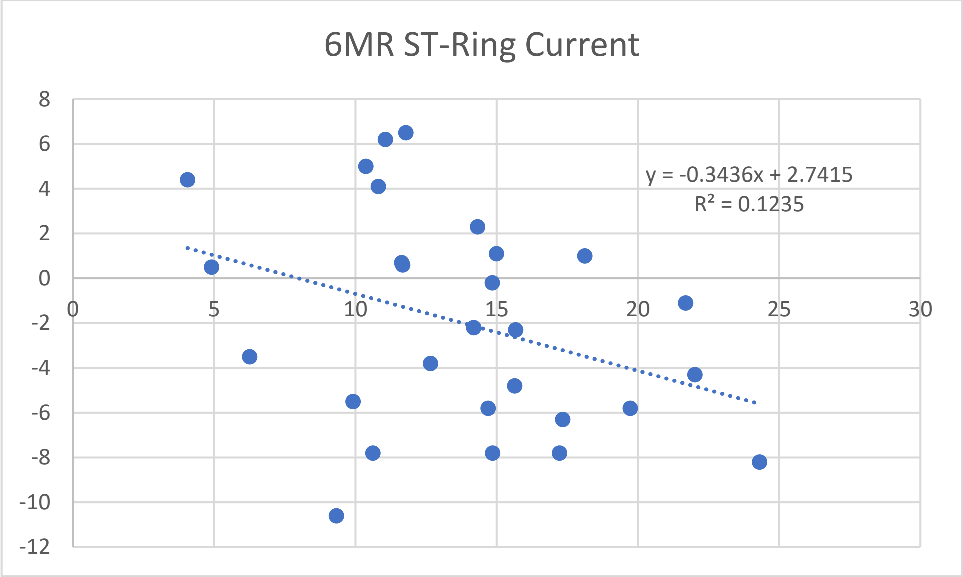


(j) Singlet-triplet gap versus ring current of the 6-membered rings among bicyclic species.

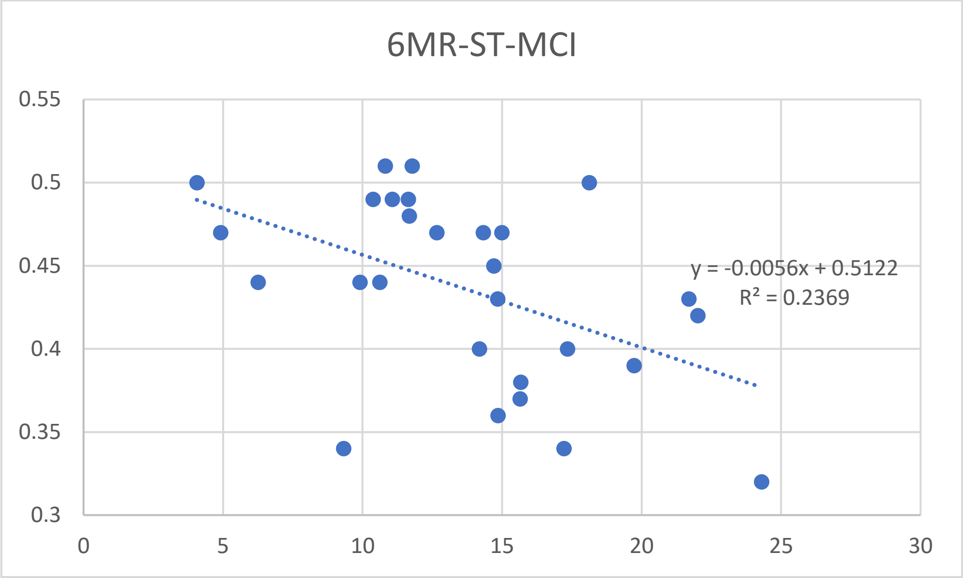


(k) Singlet-triplet gap versus MCI values of the 6-membered rings among bicyclic species.

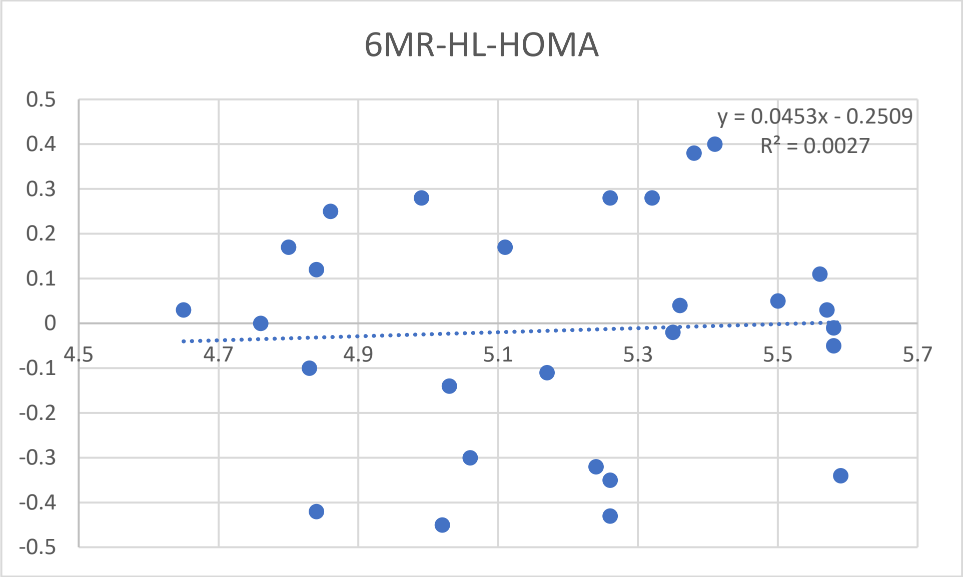


(l) HOMO-LUMO gap versus HOMA values of the 6-membered rings among bicyclic species.

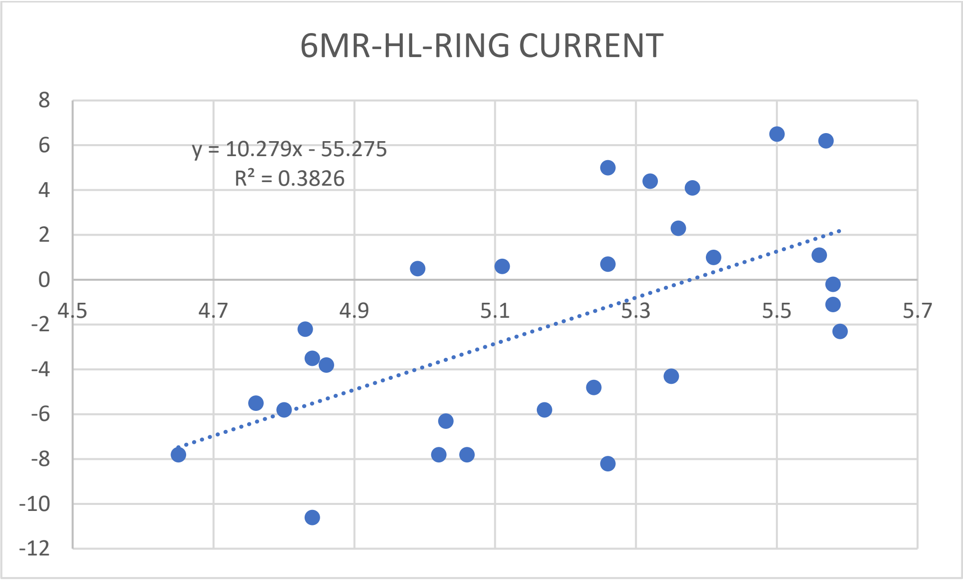


(m) HOMO-LUMO gap versus ring current values of the 6-membered rings among bicyclic species.

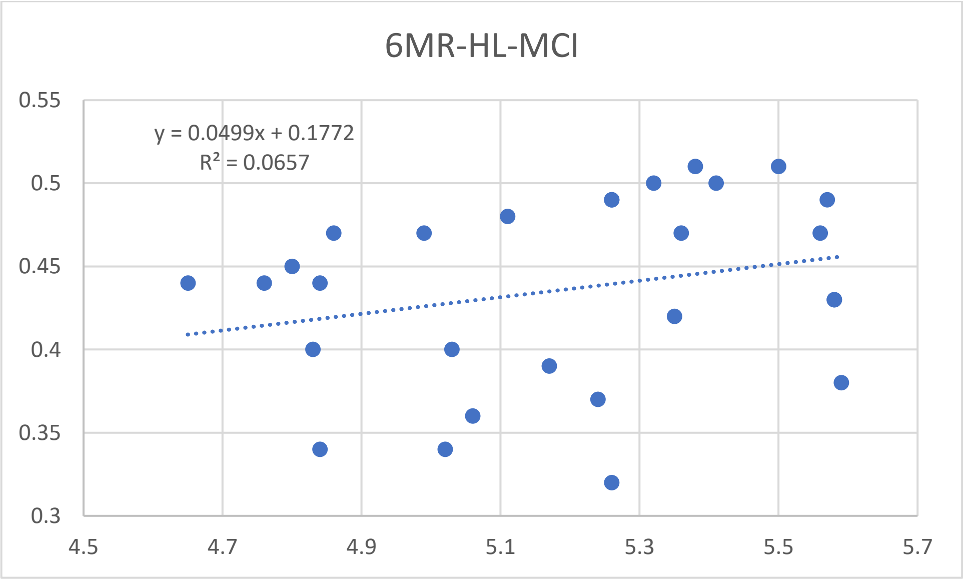


(n) HOMO-LUMO gap versus MCI values of the 6-membered rings among bicyclic species.

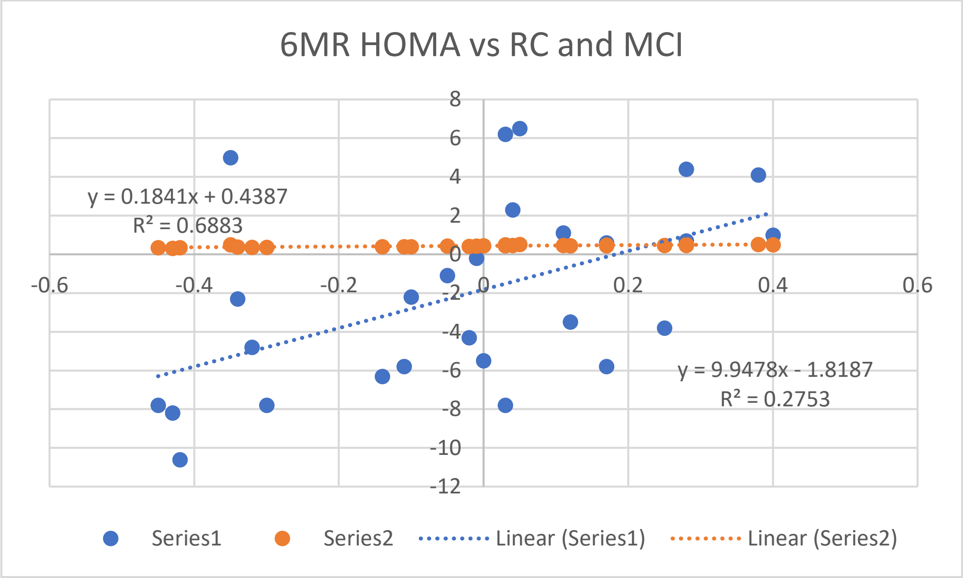


(o) HOMA vs ring current (Series 1) and MCI (Series 2) for the 6-membered rings among bicyclic species.

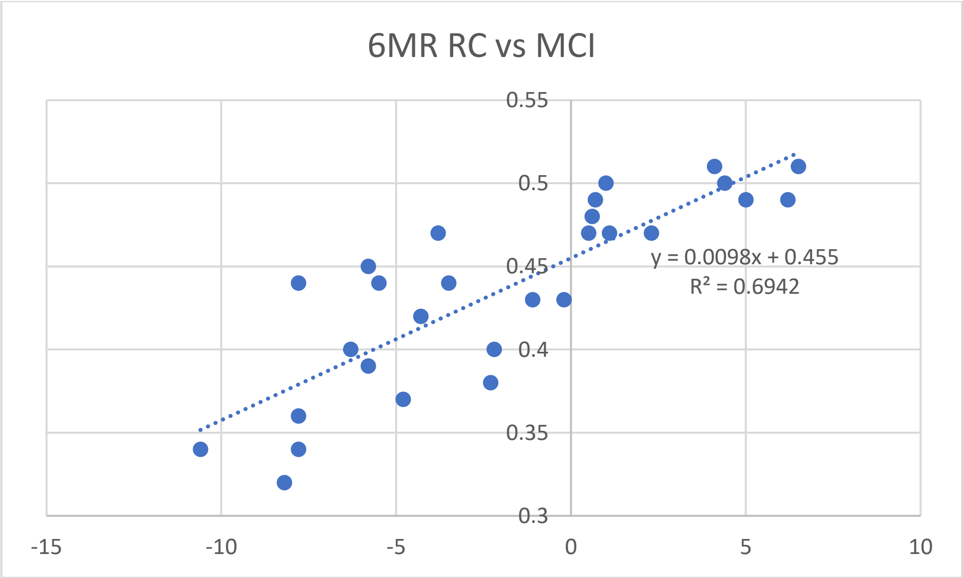


(p) Ring current versus MCI for the 6-membered rings among bicyclic species.

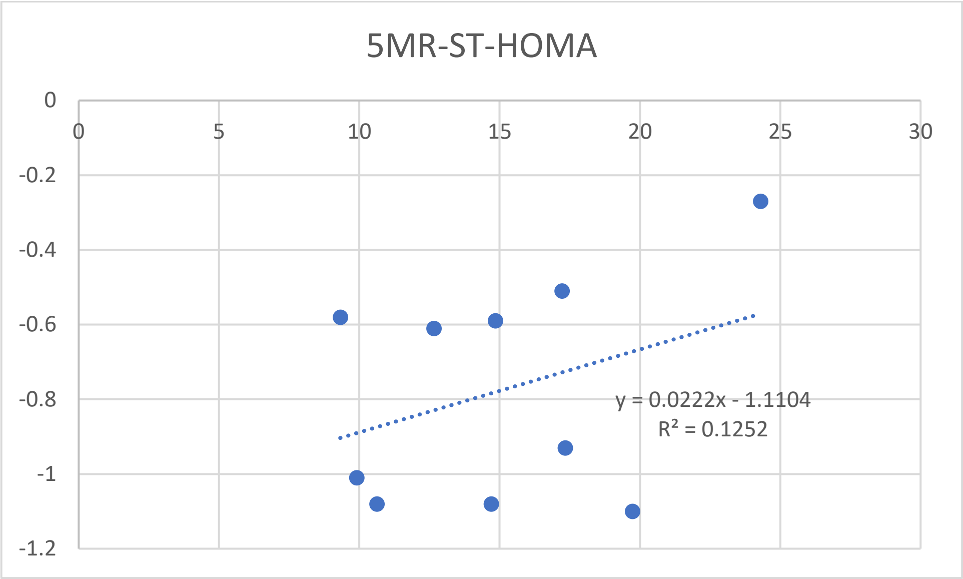


(q) Singlet-triplet gap versus HOMA values of the 5-membered rings among planar bicyclic species.

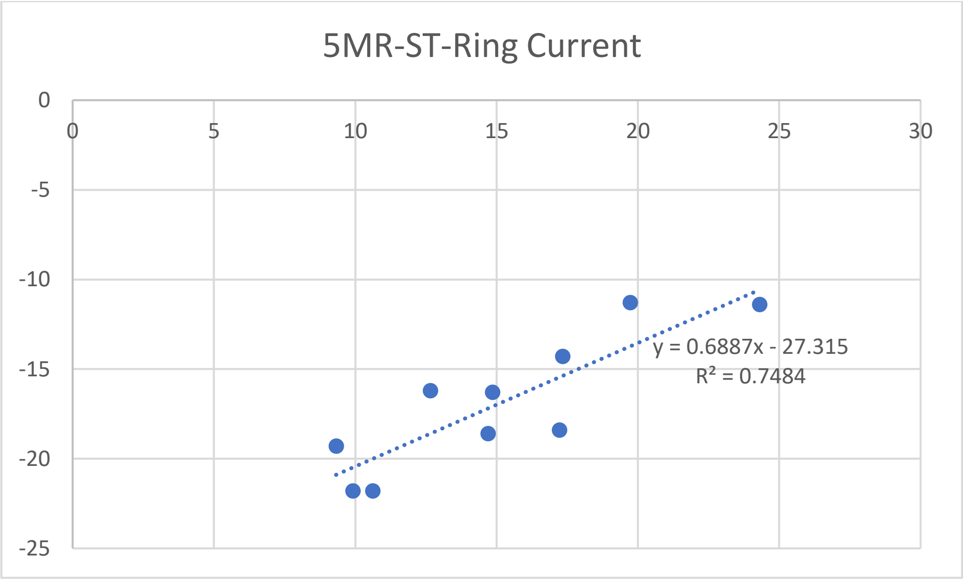


(r) Singlet-triplet gap versus ring current values of the 5-membered rings among planar bicyclic species.

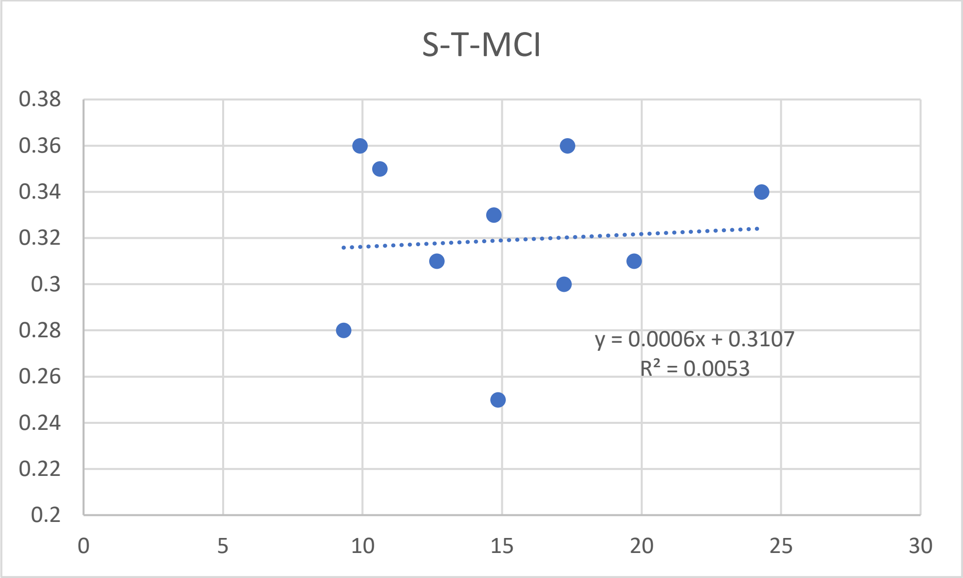


(s) Singlet-triplet gap versus MCI values of the 5-membered rings among planar bicyclic species.

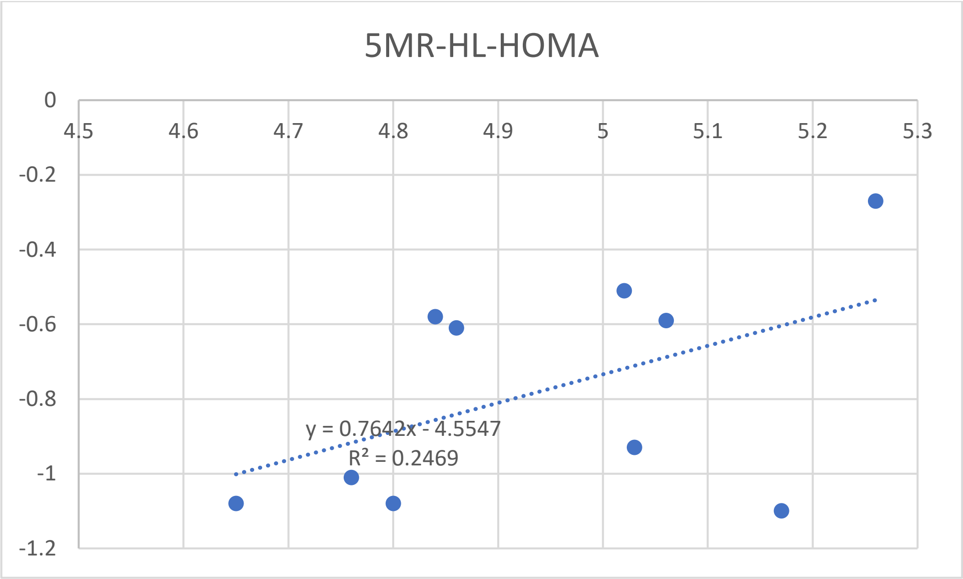


(t) HOMO-LUMO gap versus HOMA values of the 5-membered rings among planar bicyclic species.

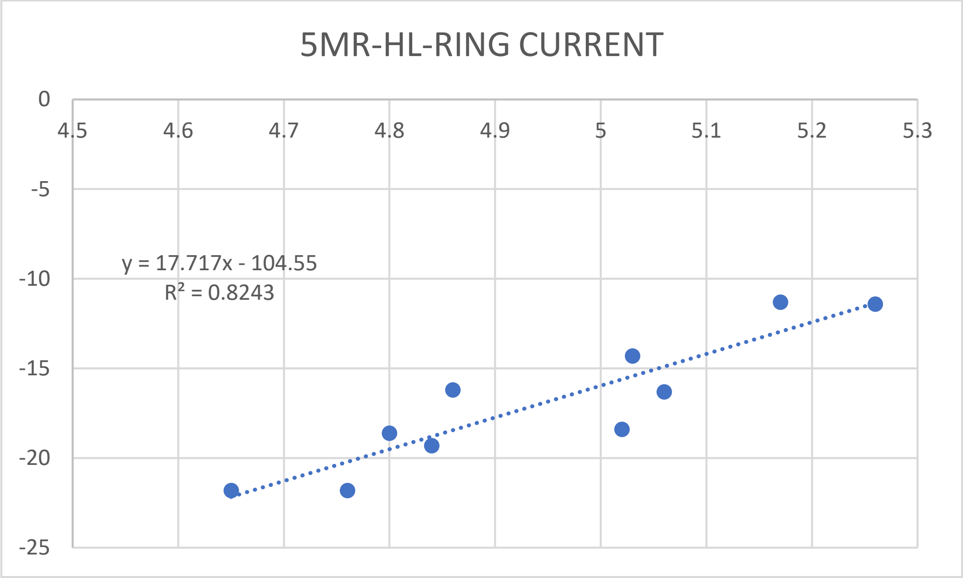


(u) HOMO-LUMO gap versus ring current values of the 5-membered rings among planar bicyclic species.

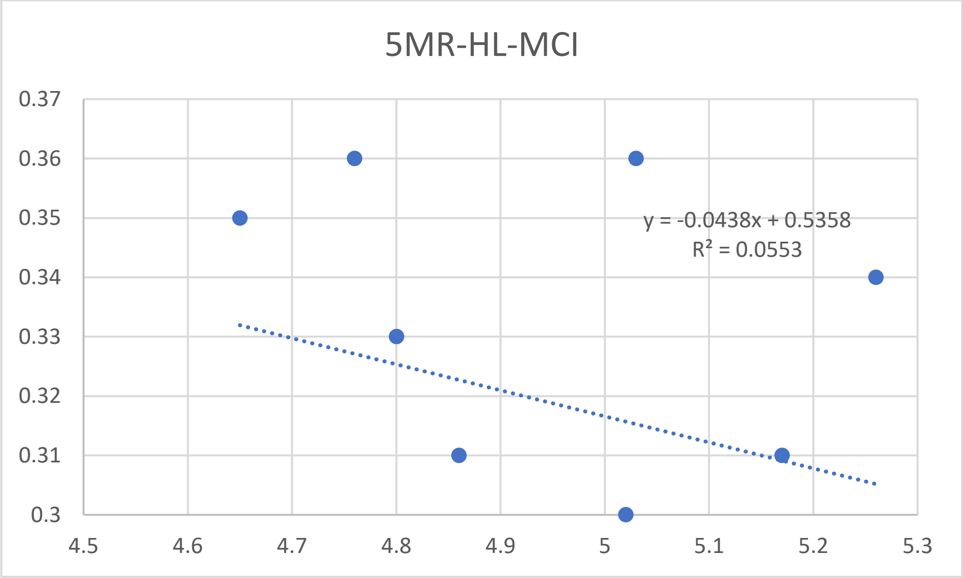


(v) HOMO-LUMO gap versus MCI values of the 5-membered rings among planar bicyclic species.

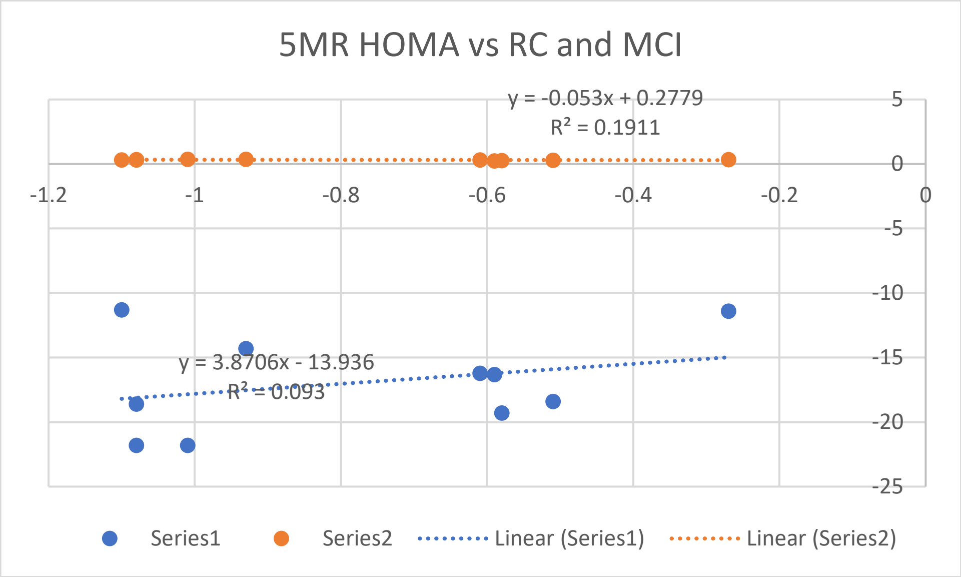


(w) HOMA vs ring current (Series 1) and MCI (Series 2) for the 5-membered rings among planar bicyclic species.

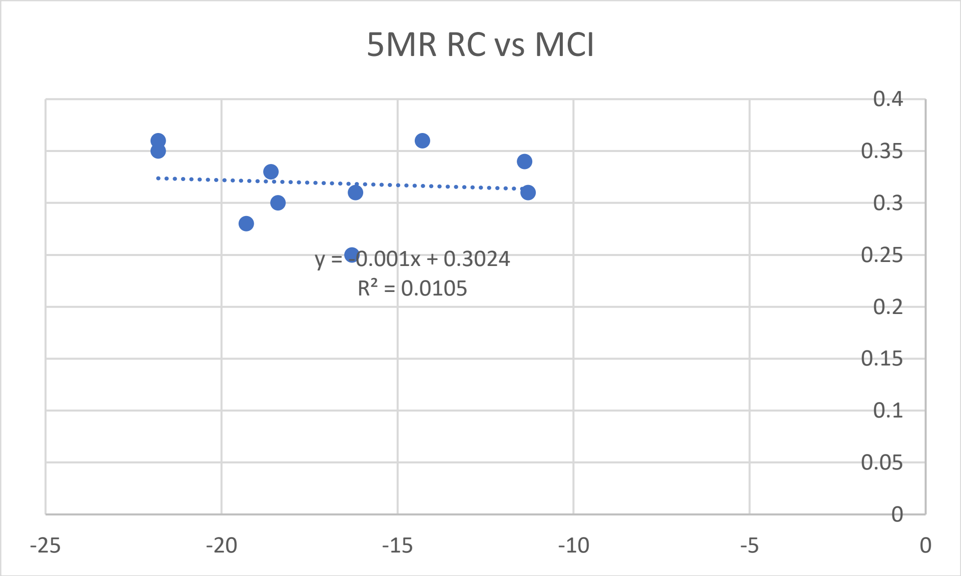


(x) Ring current versus MCI for the 5-membered rings among planar bicyclic species.

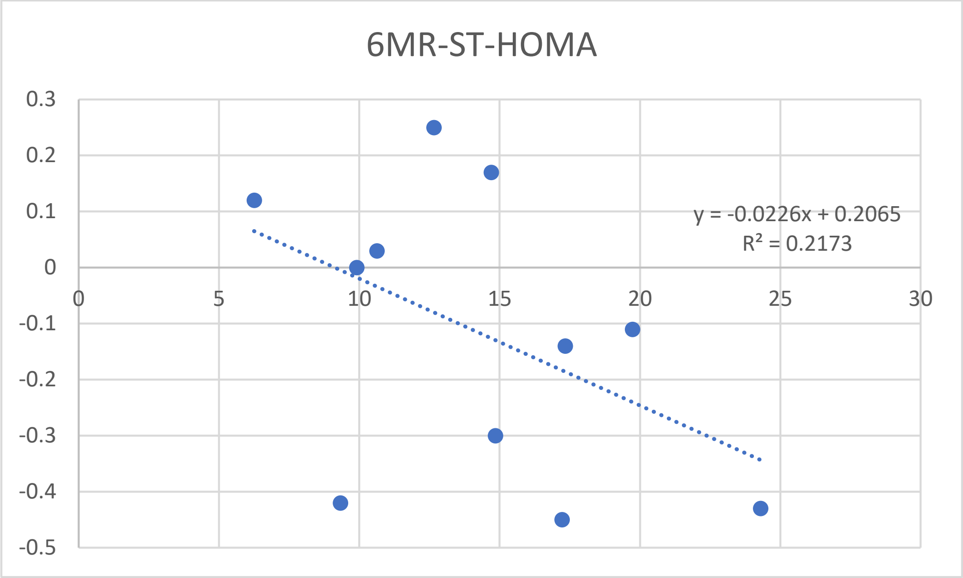


(y) Singlet-triplet gap versus HOMA values of the 6-membered rings among planar bicyclic species.

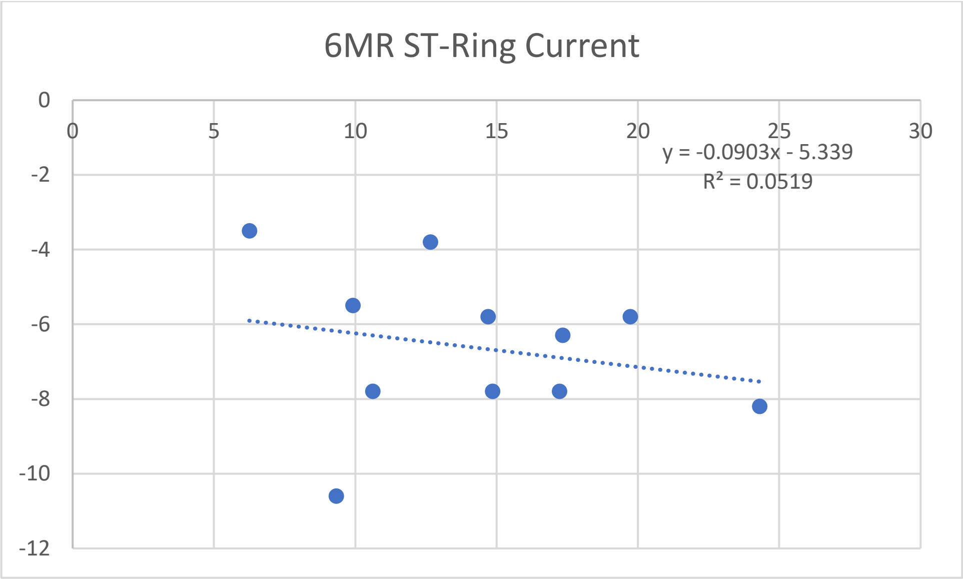


(z) Singlet-triplet gap versus ring current values of the 6-membered rings among planar bicyclic species.

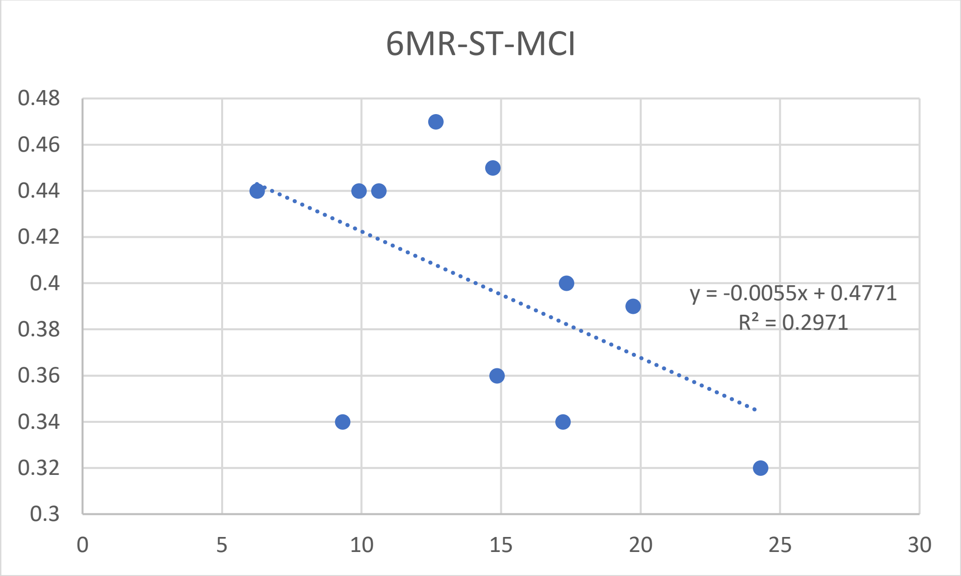


(aa) Singlet-triplet gap versus MCI values of the 6-membered rings among planar bicyclic species.

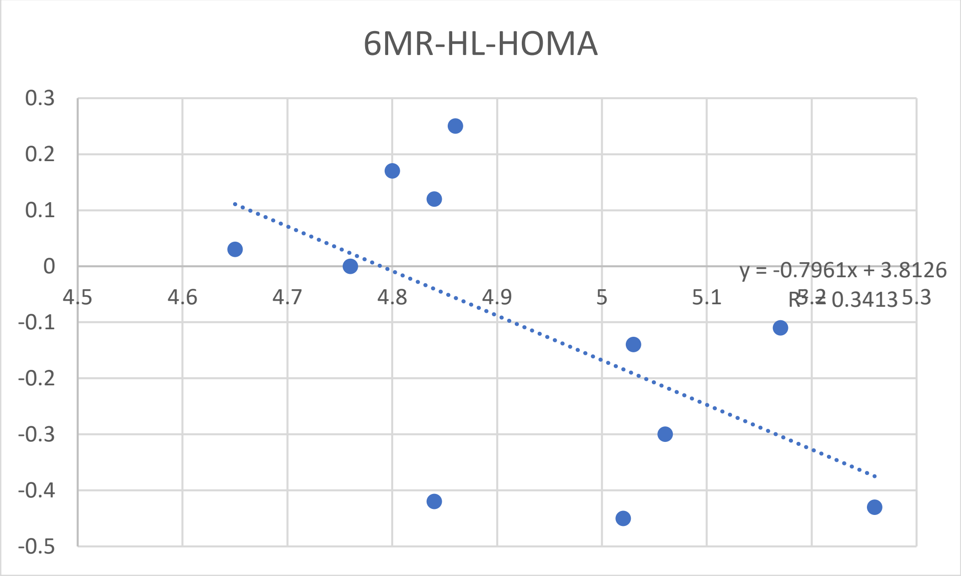


(ba) HOMO-LUMO gap versus HOMA values of the 6-membered rings among planar bicyclic species.

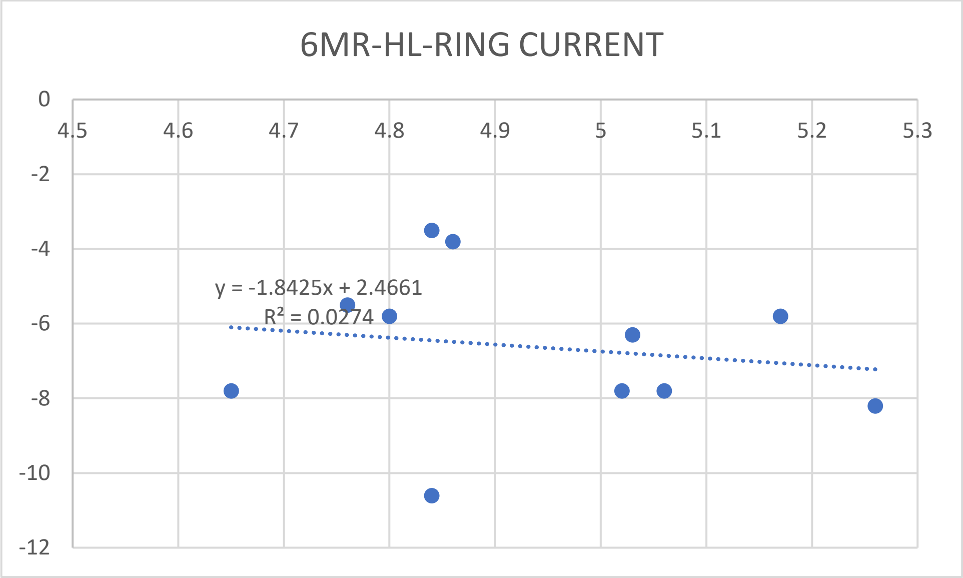


(ca) HOMO-LUMO gap versus ring current values of the 6-membered rings among planar bicyclic species.

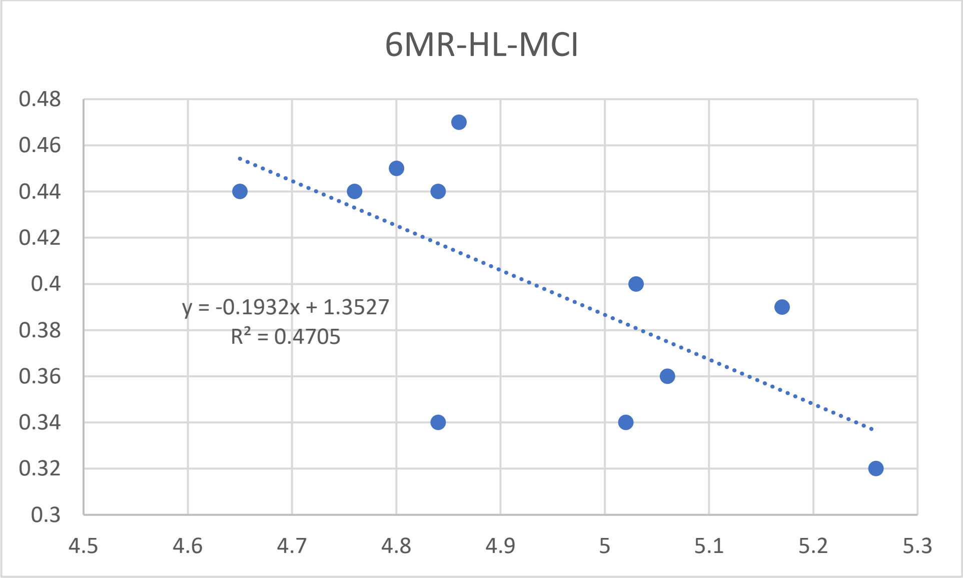


(da) HOMO-LUMO gap versus MCI values of the 6-membered rings among planar bicyclic species.

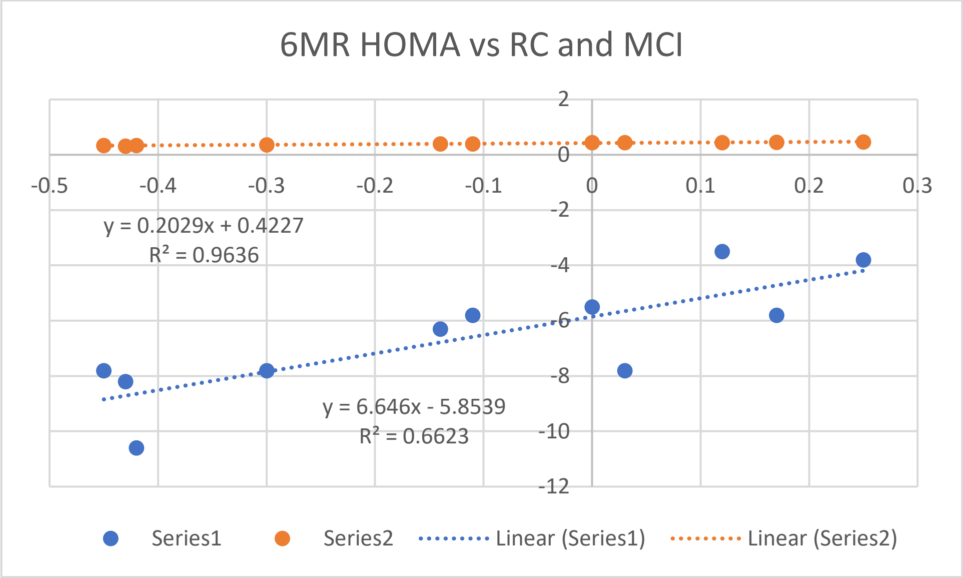


(ea) HOMA vs ring current (Series 1) and MCI (Series 2) for the 6-membered rings among planar bicyclic species.

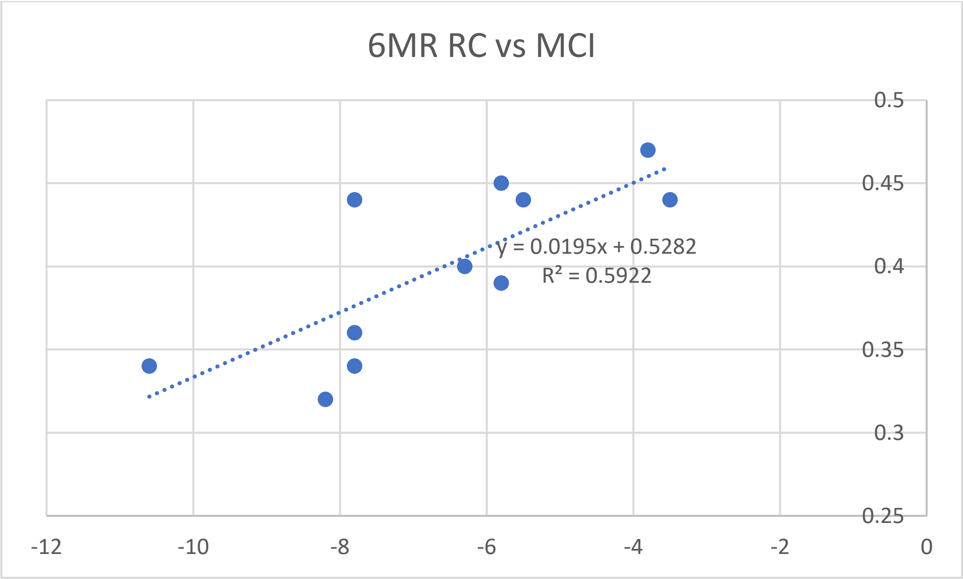


(fa) Ring current versus MCI for the 6-membered rings among planar bicyclic species.

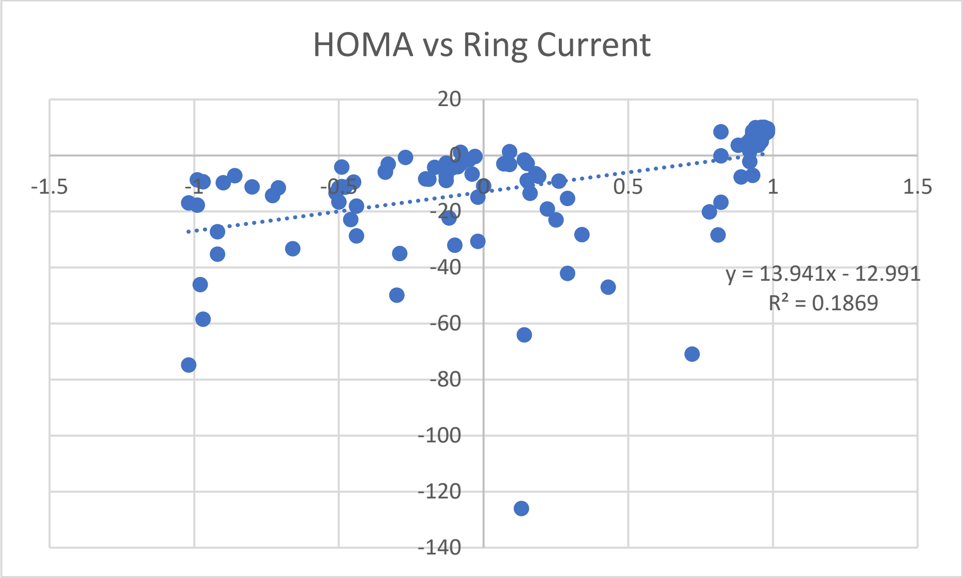


(ga) HOMA versus ring current for all rings among polycyclic species.

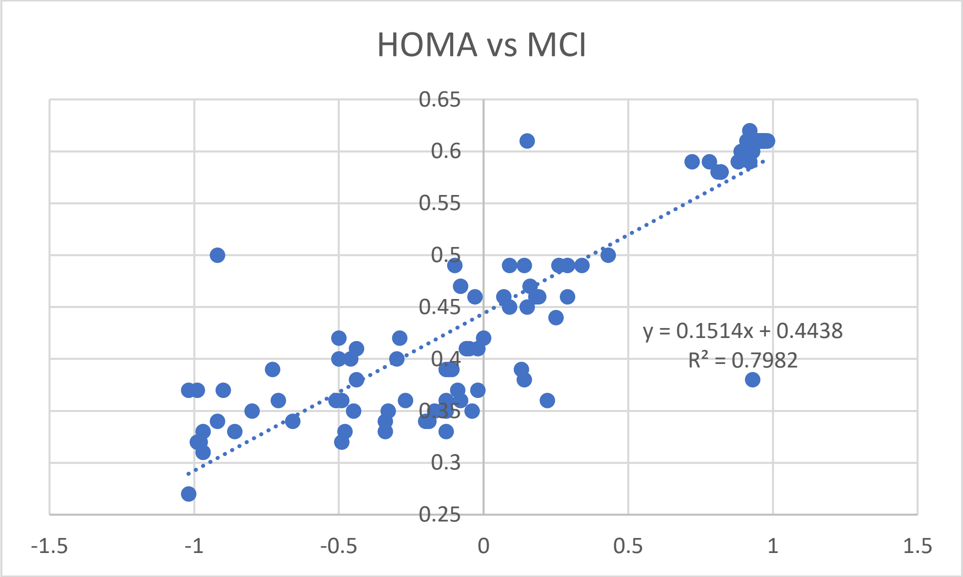


(ha) HOMA versus MCI for all rings among polycyclic species.

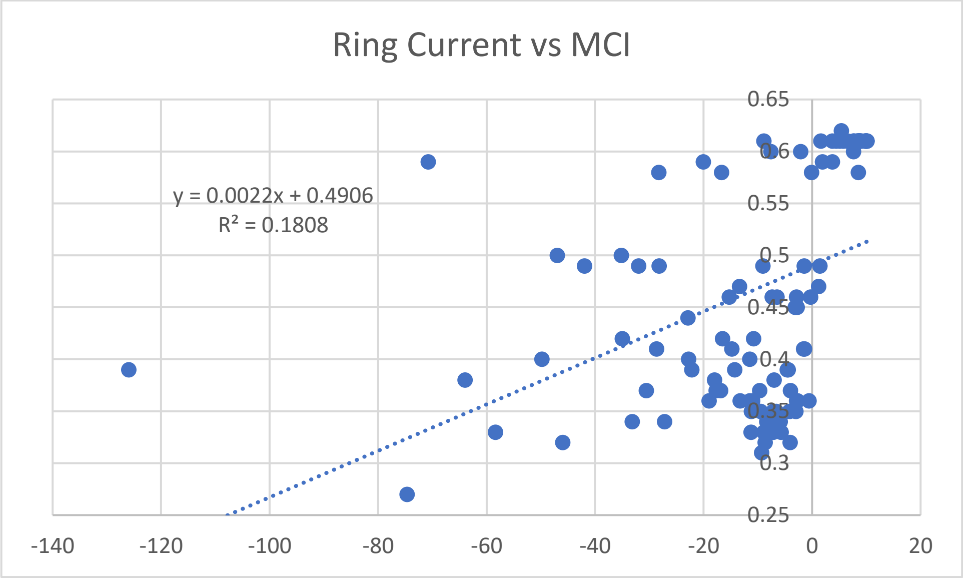


(ia) Ring current versus MCI for all rings among polycyclic species.

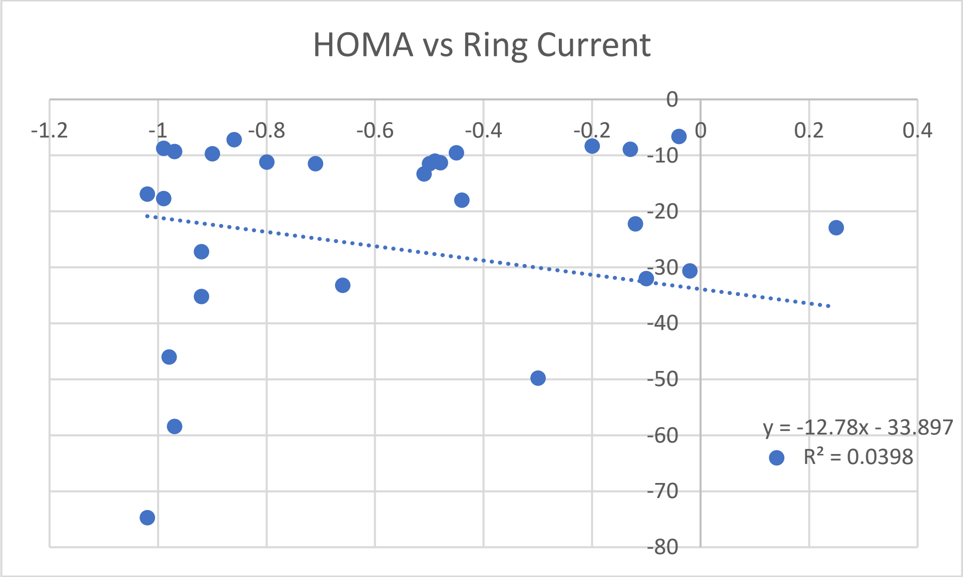


(ja) HOMA versus ring current for 5-membered rings among polycyclic species.

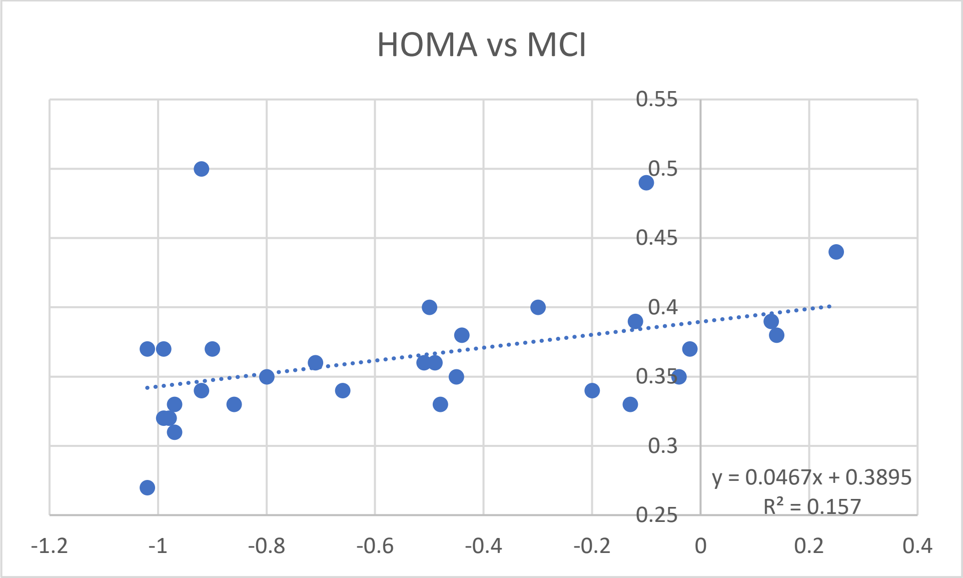


(ka) HOMA versus MCI for 5-membered rings among polycyclic species.

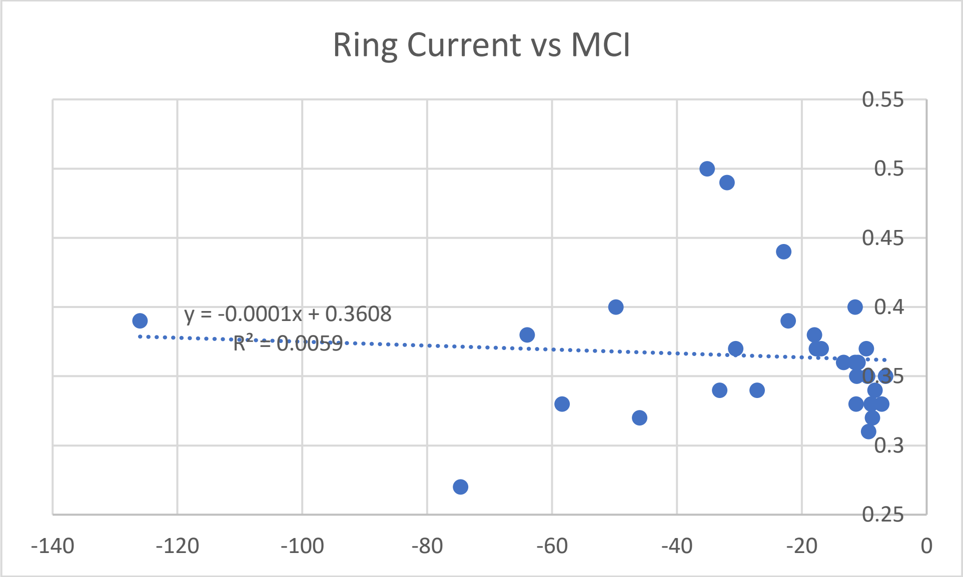


(la) Ring current versus MCI for 5-membered rings among polycyclic species.

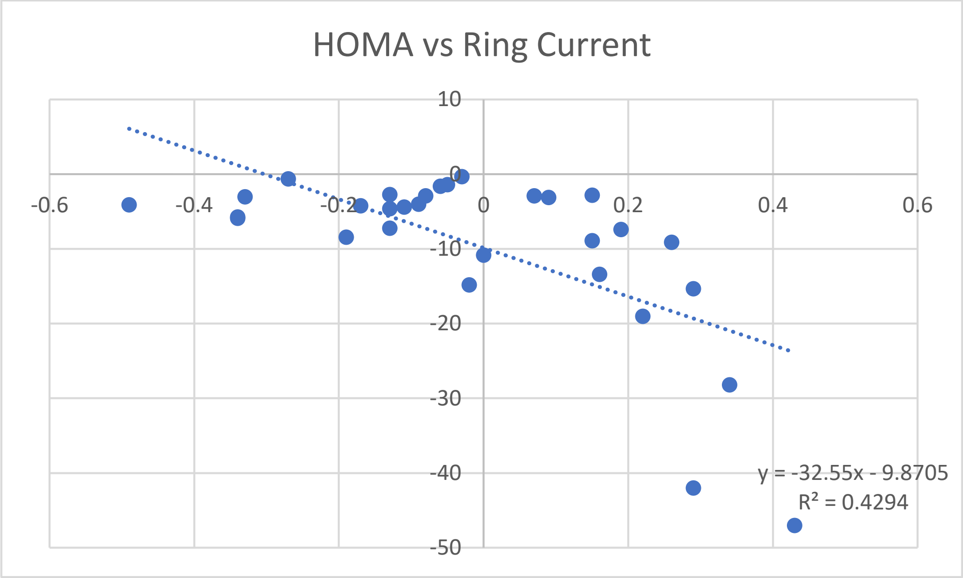


(ma) HOMA versus ring current for 6-membered rings among polycyclic species.

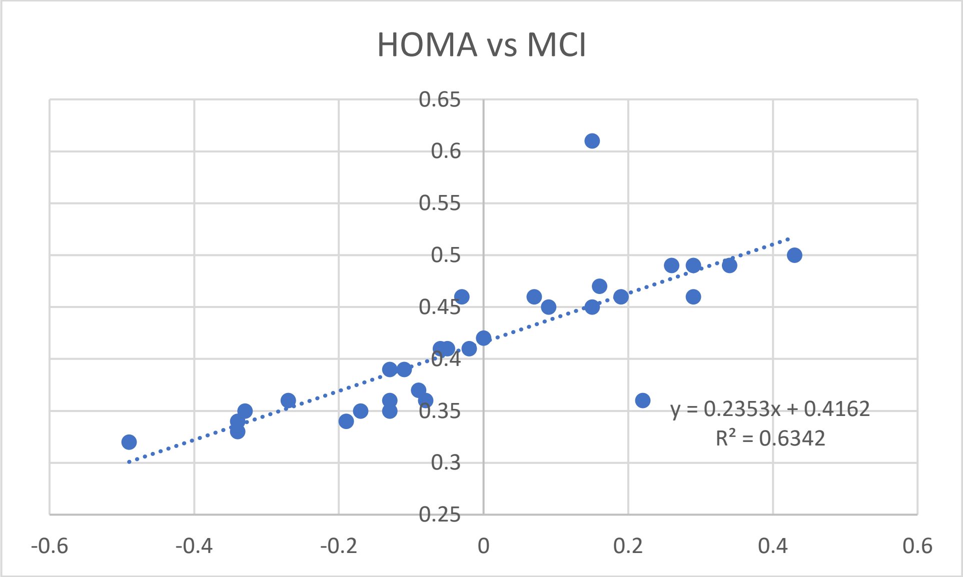


(na) HOMA versus MCI for 6-membered rings among polycyclic species.

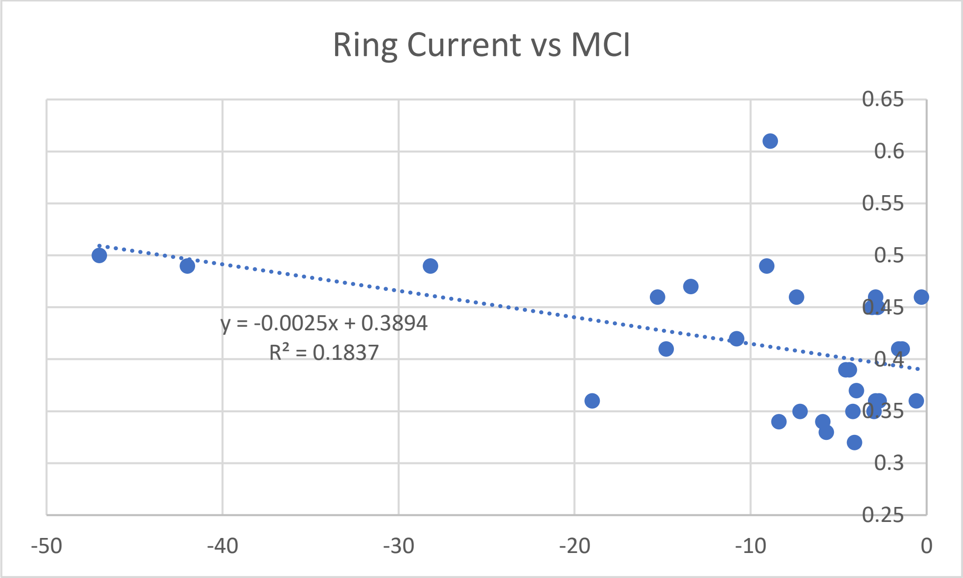


(oa) Ring current versus MCI for 6-membered rings among polycyclic species.

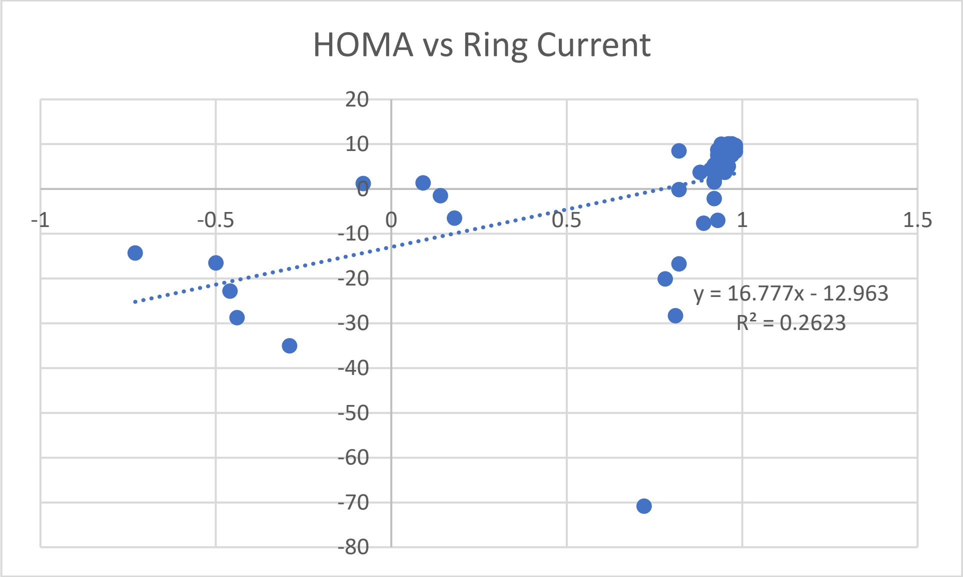


(pa) HOMA versus ring current for benzene rings among polycyclic species.

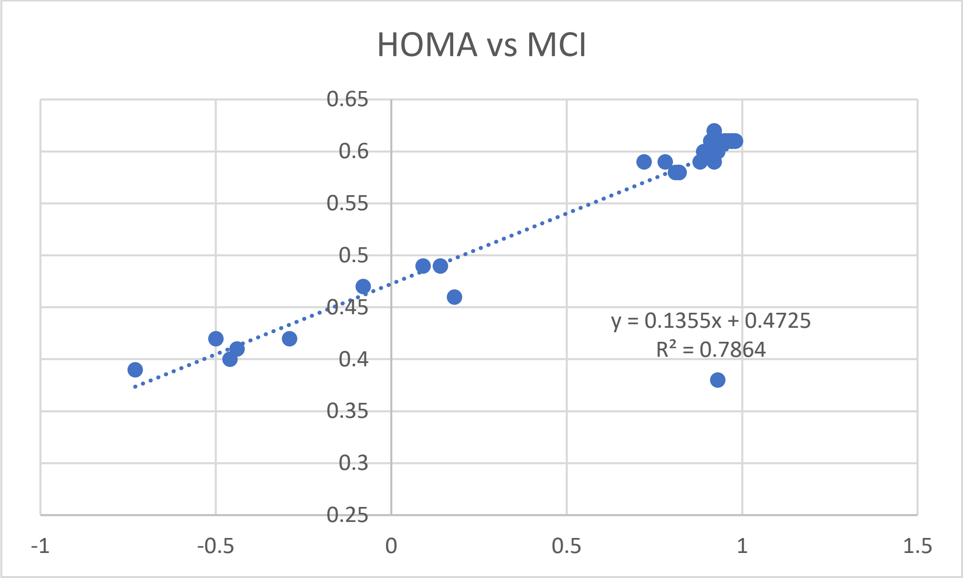


(qa) HOMA versus MCI for benzene rings among polycyclic species.

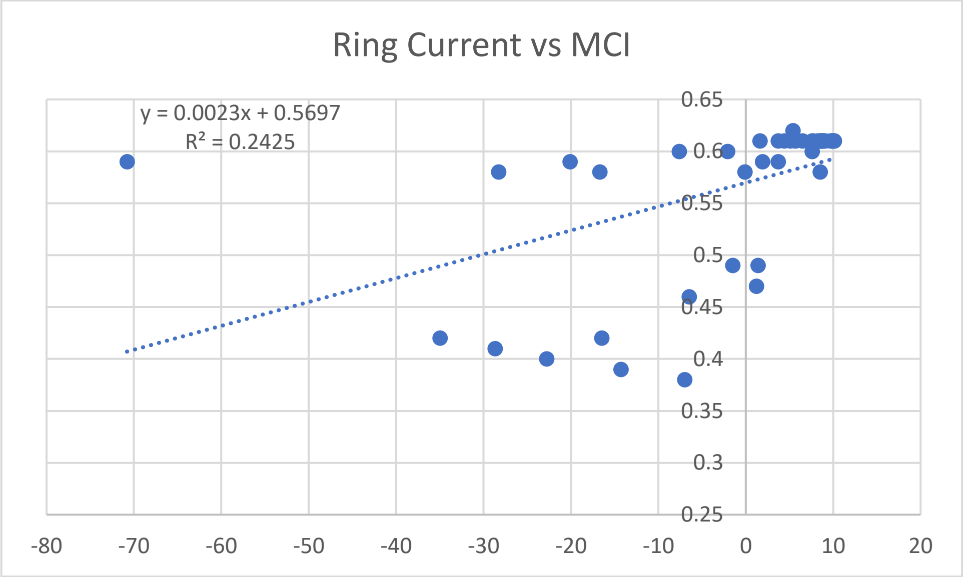


(ra) Ring current versus MCI for benzene rings among polycyclic species.

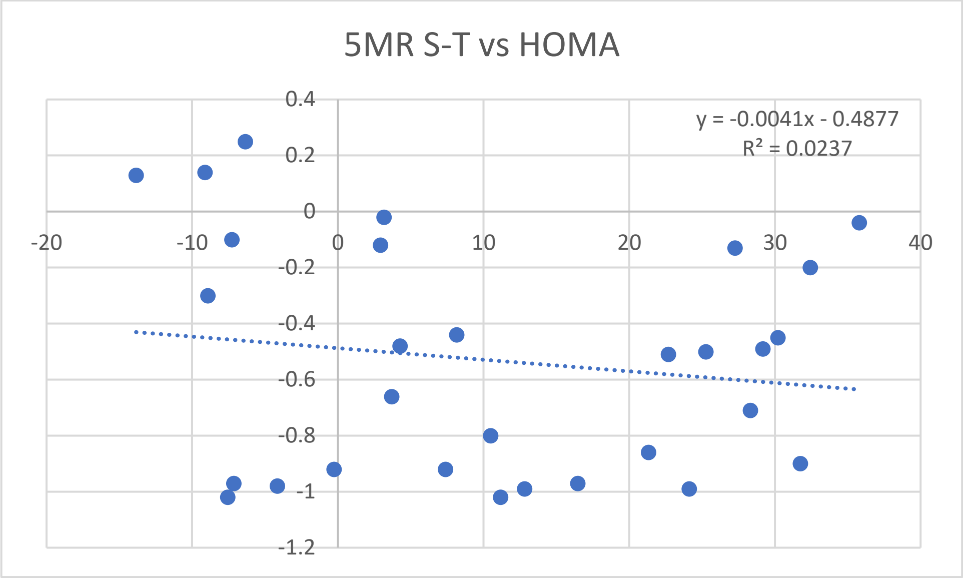


(sa) Singlet-triplet gap versus HOMA for 5-membered rings among polycyclic species.

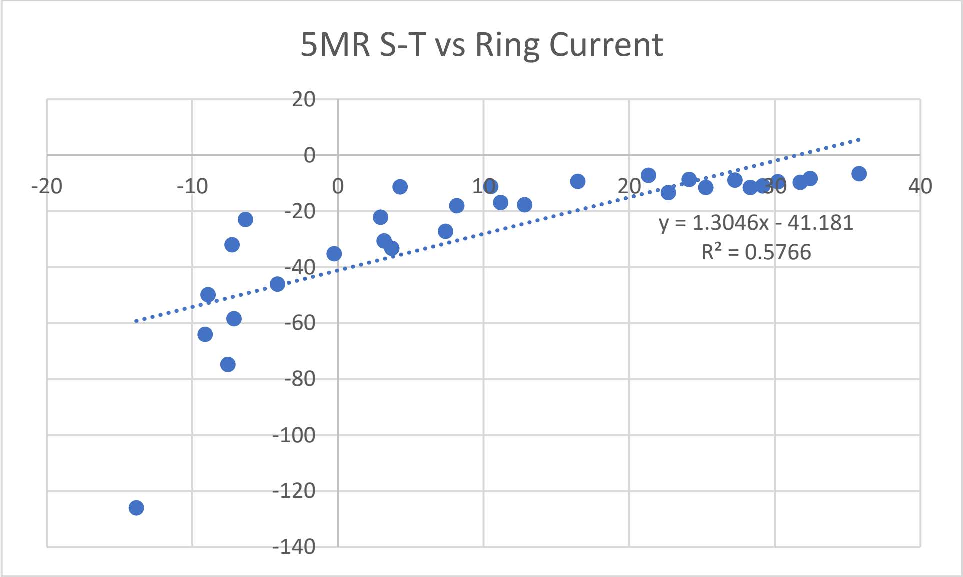


(ta) Singlet-triplet gap versus ring current for 5-membered rings among polycyclic species.

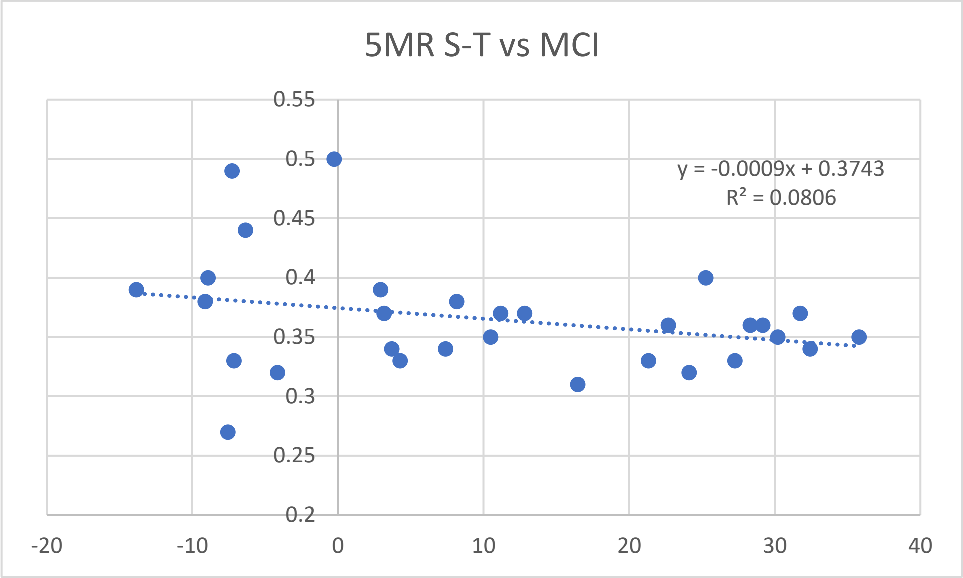


(ua) Singlet-triplet gap versus MCI for 5-membered rings among polycyclic species.

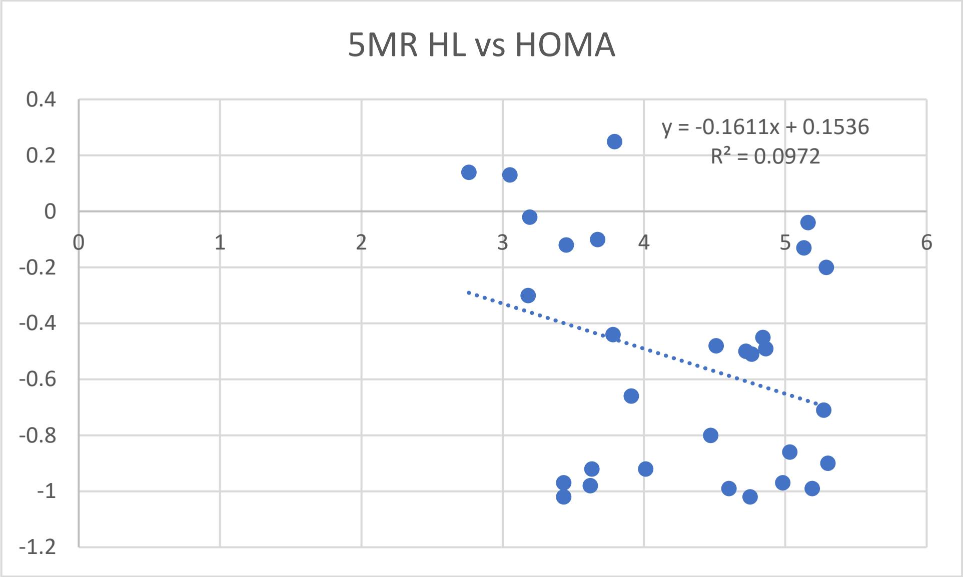


(va) HOMO-LUMO gap versus HOMA for 5-membered rings among polycyclic species.

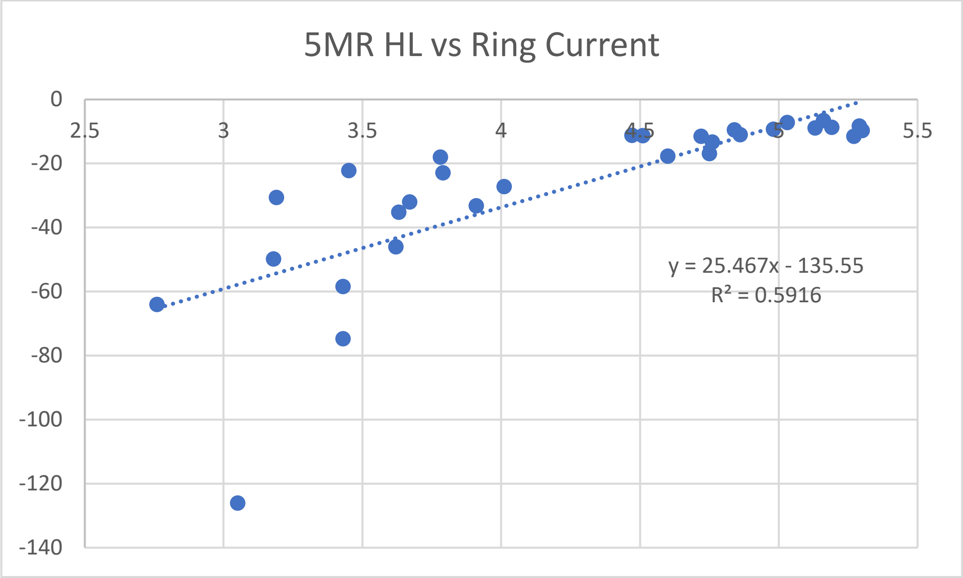


(wa) HOMO-LUMO gap versus ring current for 5-membered rings among polycyclic species.

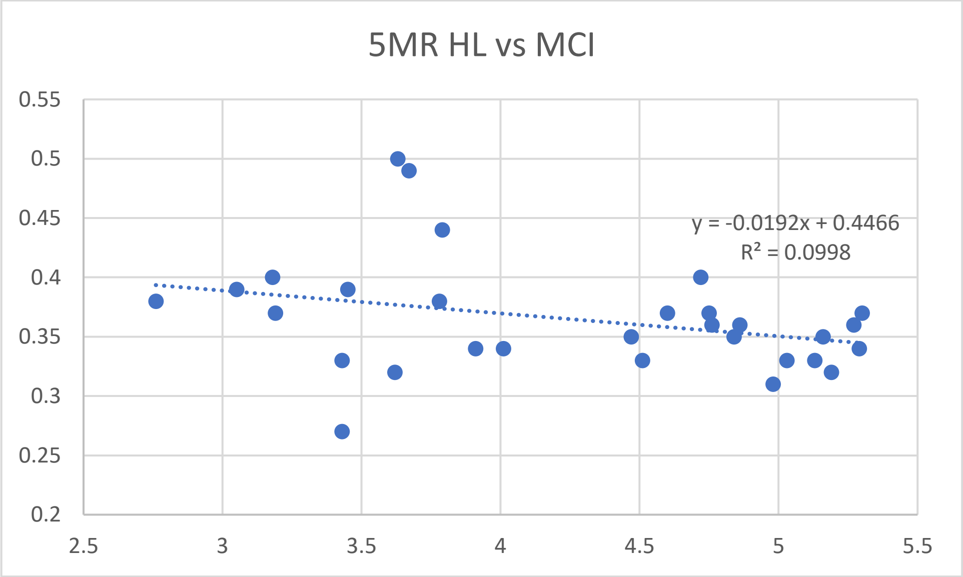


(xa) HOMO-LUMO gap versus MCI for 5-membered rings among polycyclic species.

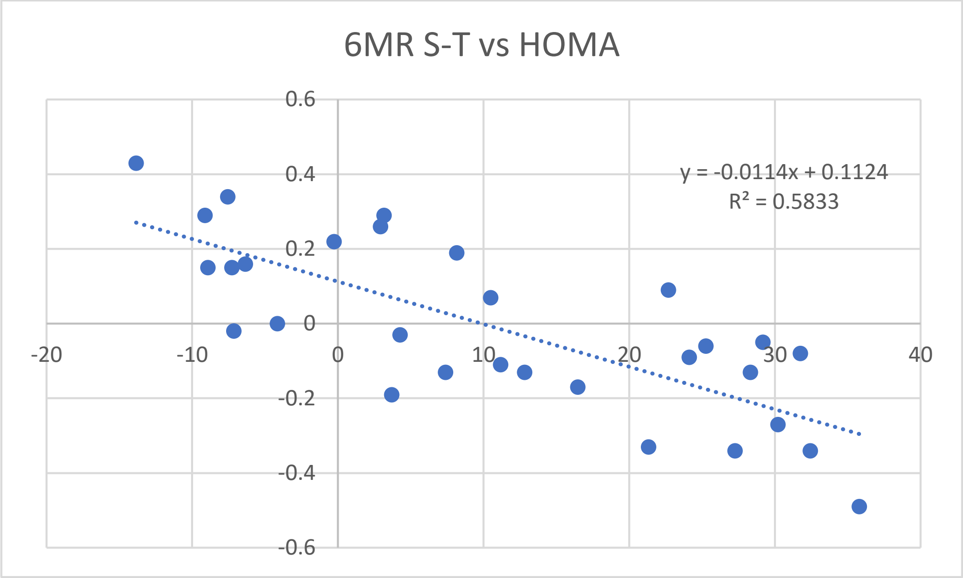


(ya) Singlet-triplet gap versus HOMA for 6-membered rings among polycyclic species.

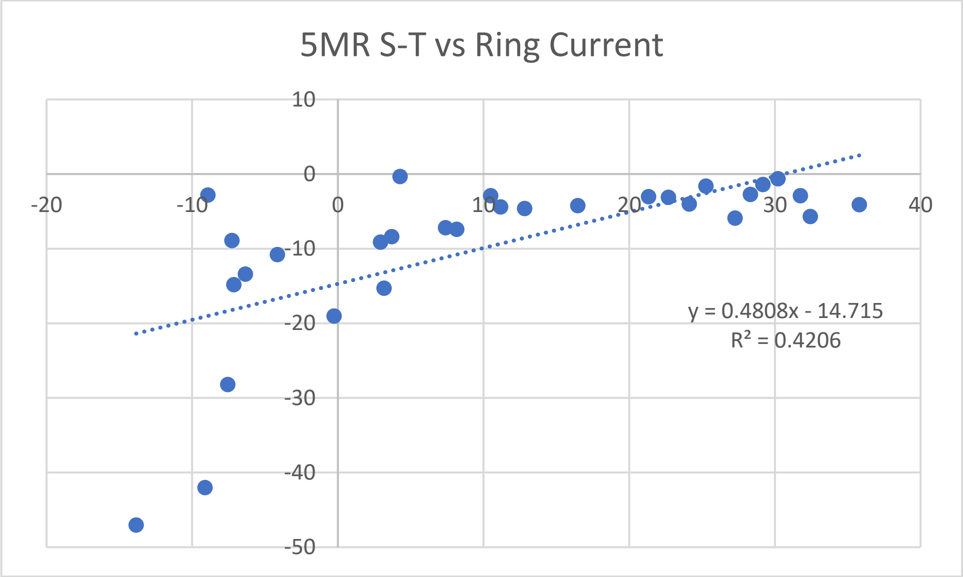


(za) Singlet-triplet gap versus ring current for 6-membered rings among polycyclic species.

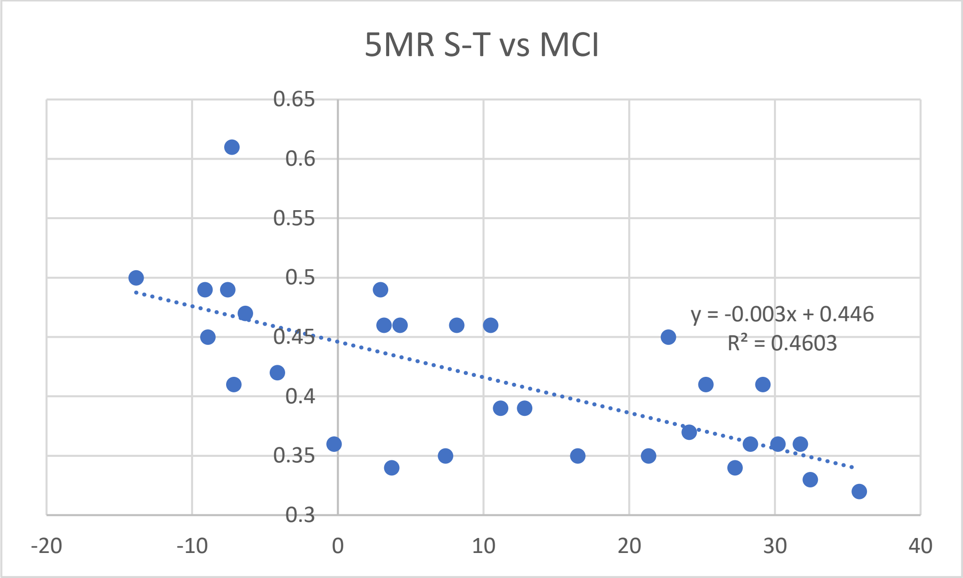


(ab) Singlet-triplet gap versus MCI for 6-membered rings among polycyclic species.

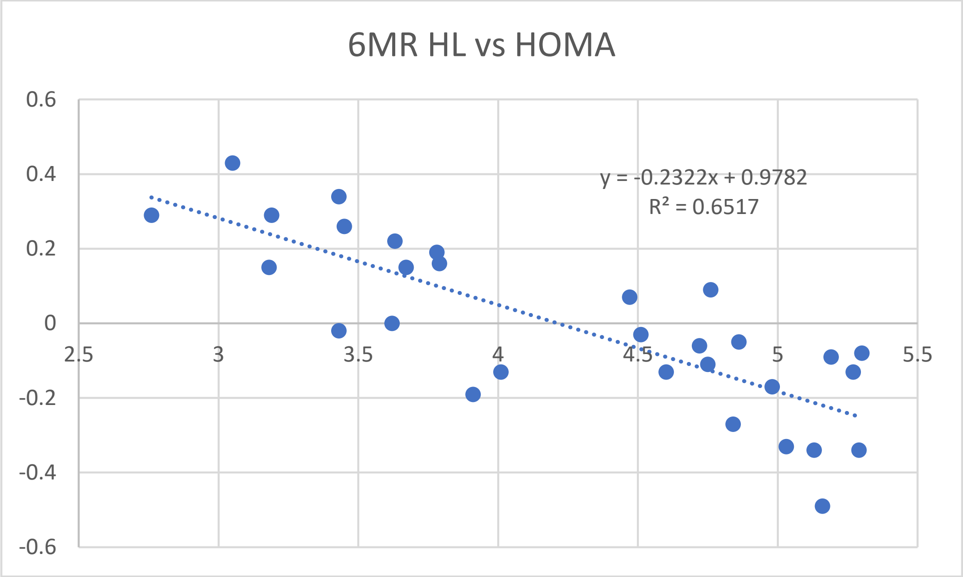


(bb) HOMO-LUMO gap versus HOMA for 6-membered rings among polycyclic species.

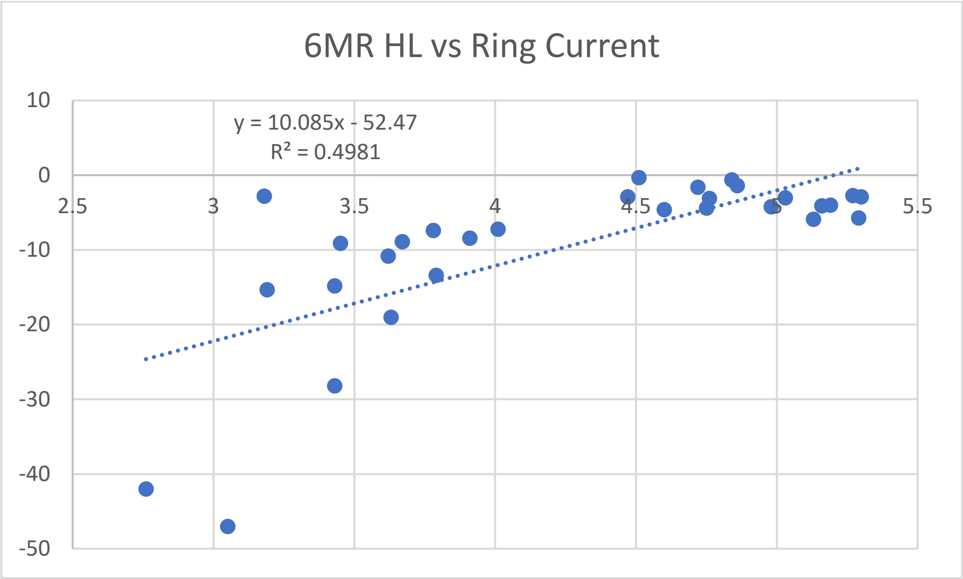


(cb) HOMO-LUMO gap versus ring current for 6-membered rings among polycyclic species.

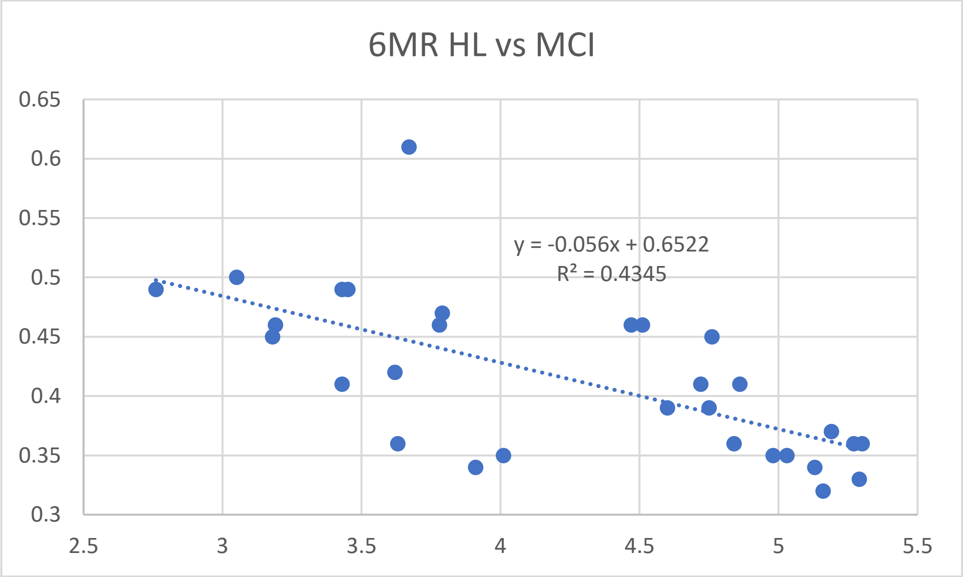


(db) HOMO-LUMO gap versus MCI for 6-membered rings among polycyclic species.

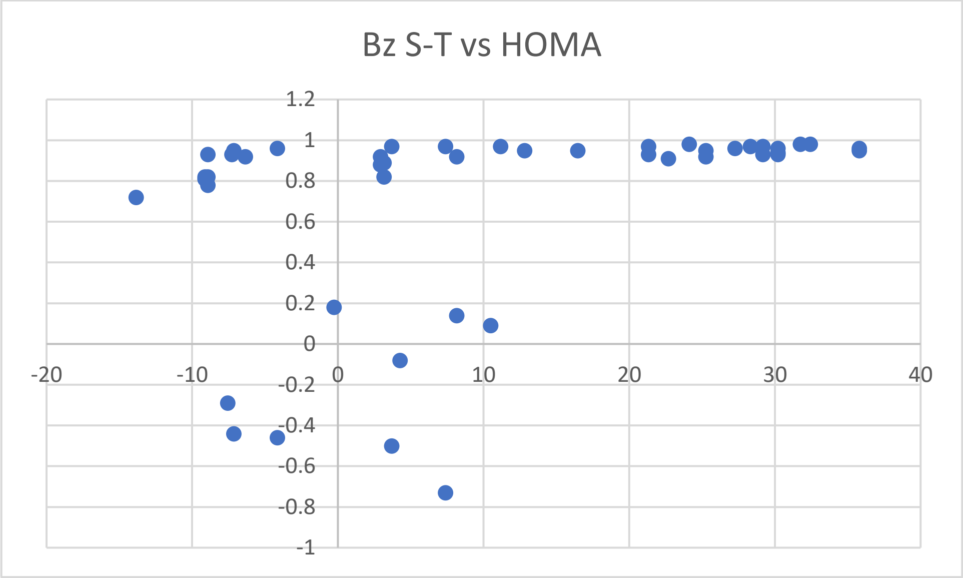


(eb) Singlet-triplet gap versus HOMA for benzene rings among polycyclic species.

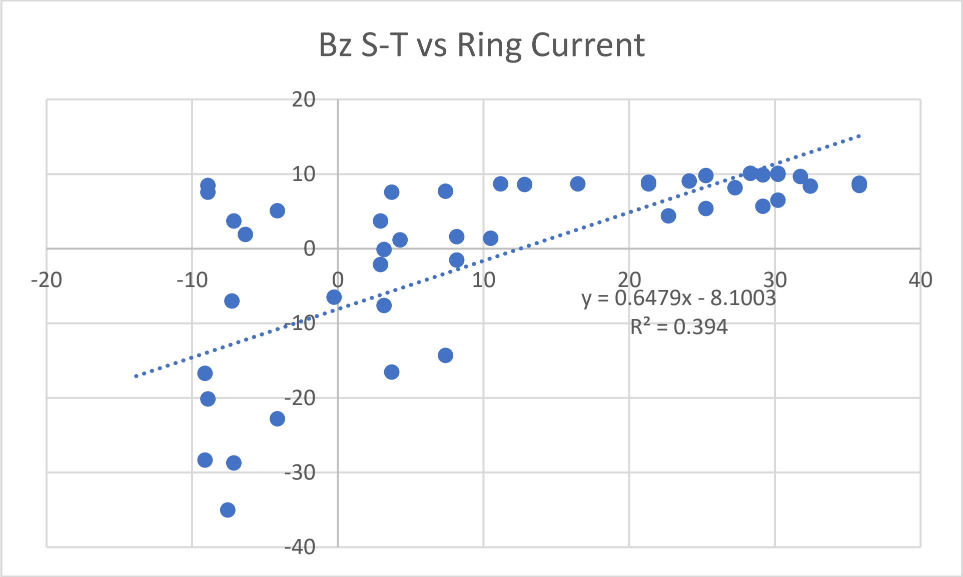


(fb) Singlet-triplet gap versus ring current for benzene rings among polycyclic species.

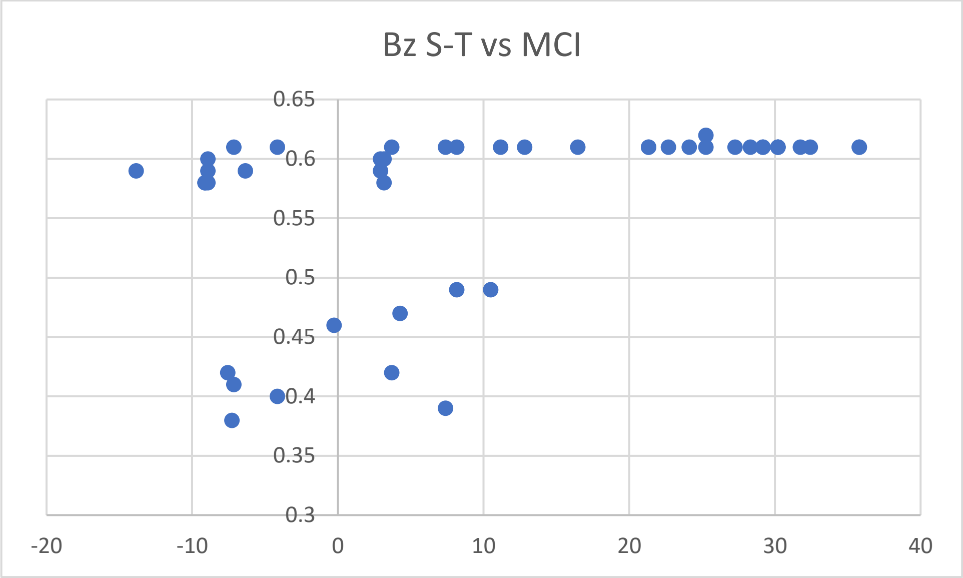


(gb) Singlet-triplet gap versus MCI for benzene rings among polycyclic species.

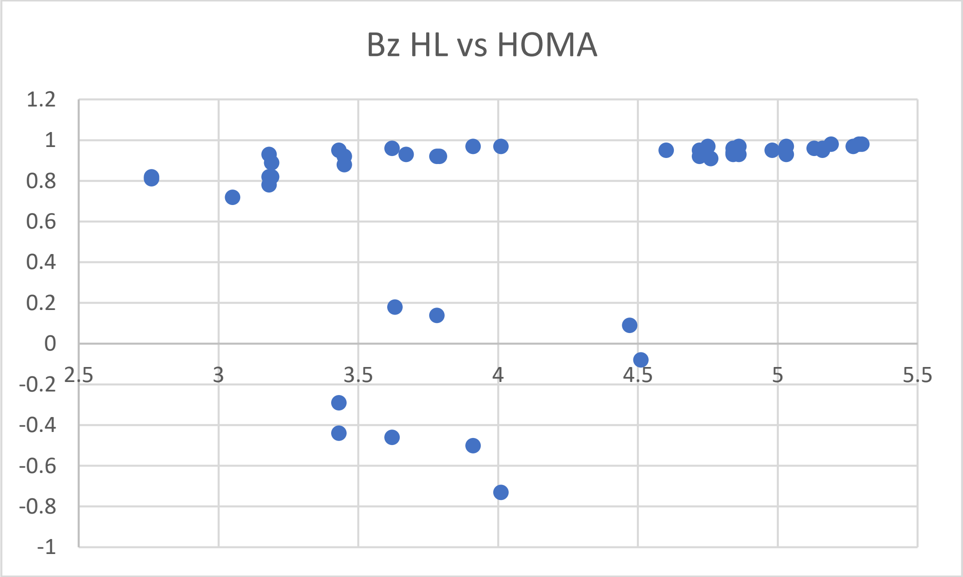


(hb) HOMO-LUMO gap versus HOMA for benzene rings among polycyclic species.

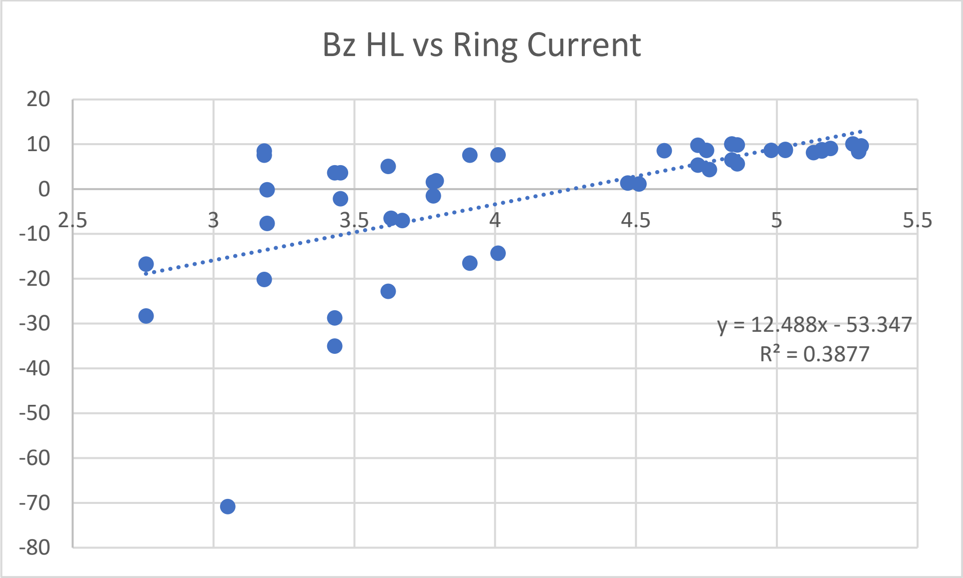


(ib) HOMO-LUMO gap versus ring current for benzene rings among polycyclic species.

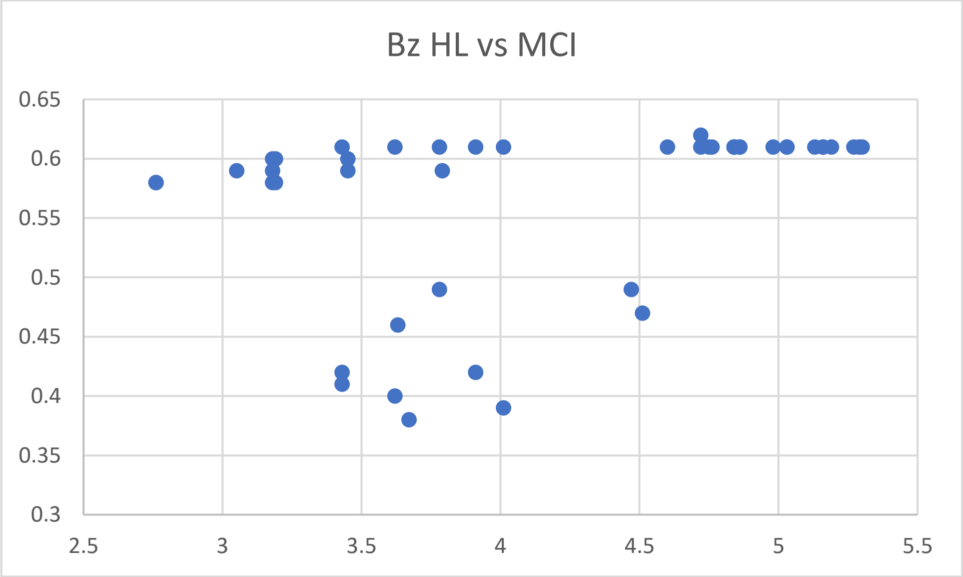


(jb) HOMO-LUMO gap versus MCI for benzene rings among polycyclic species.

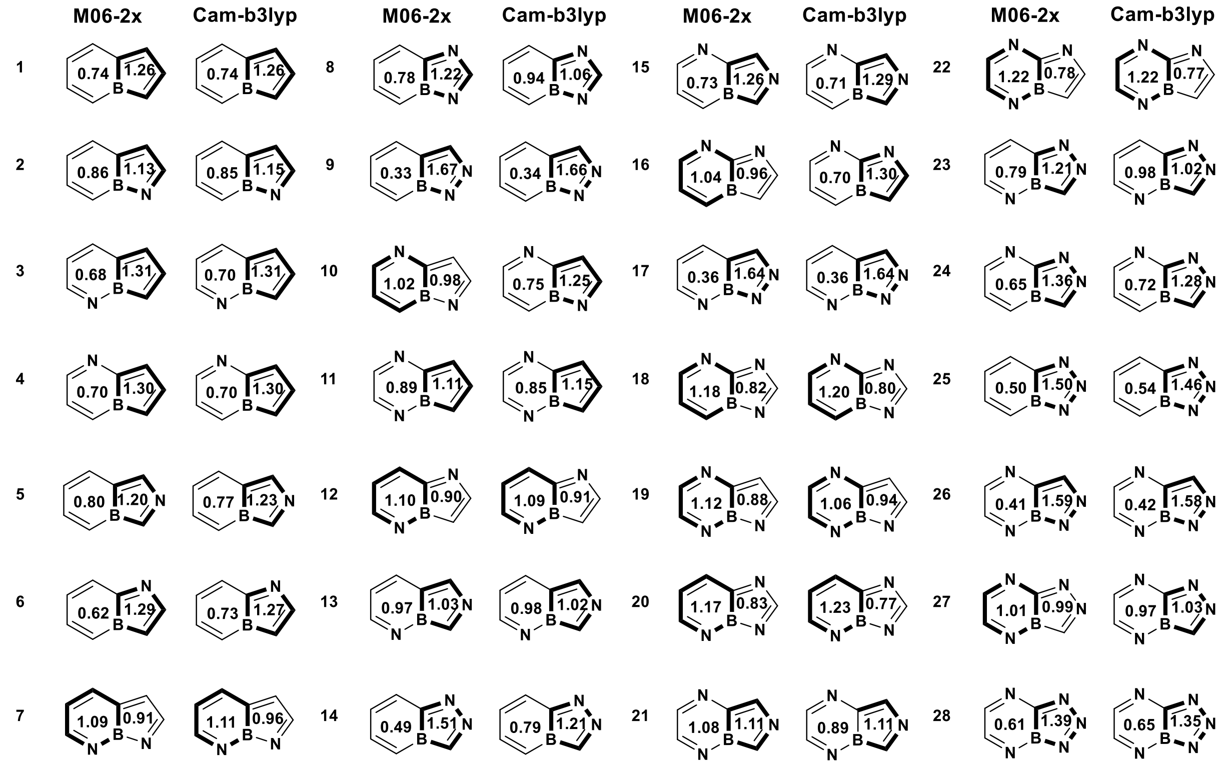


**Figure S4**. Ring spin densities of the studied bicyclic molecules

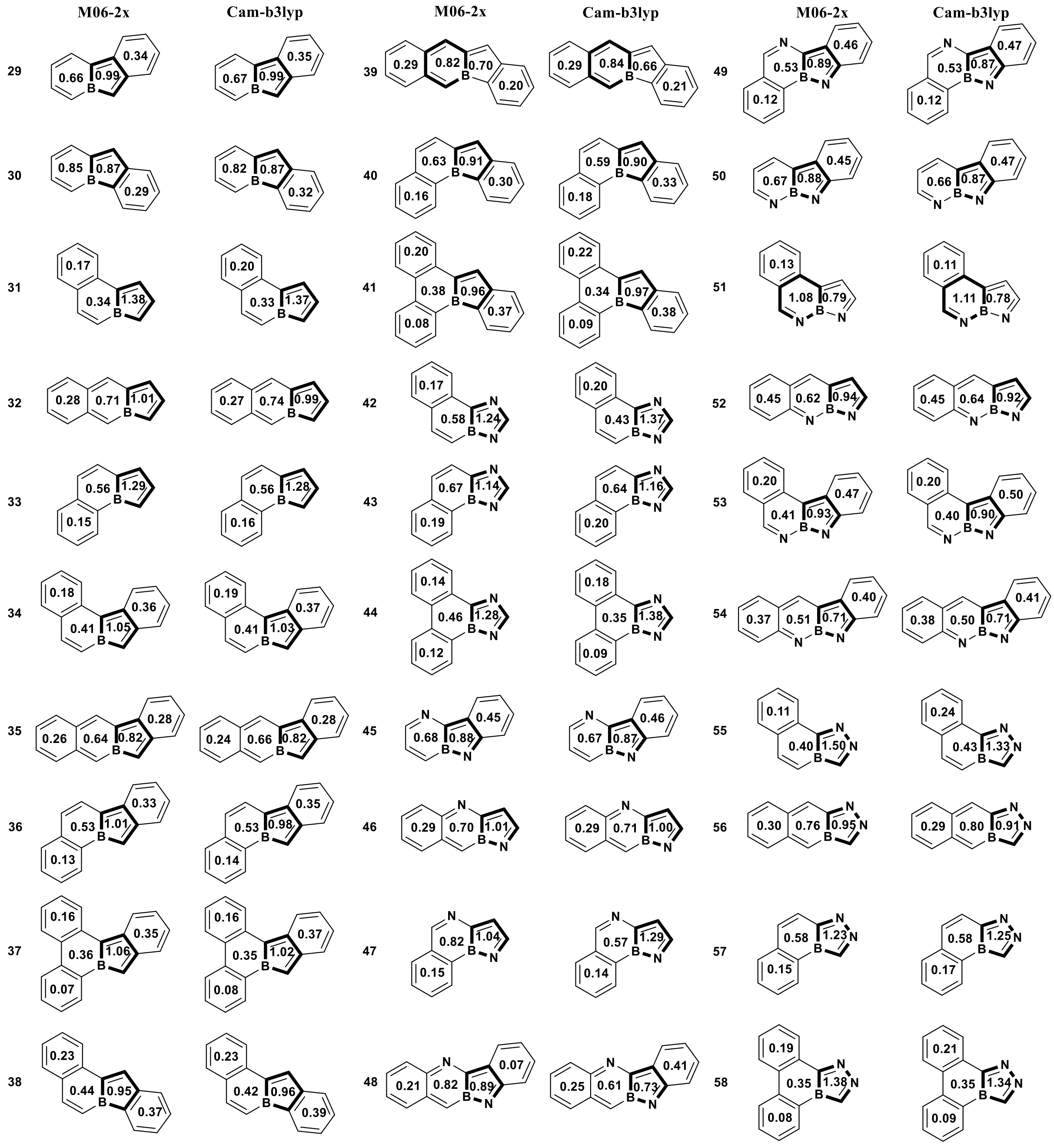


**Figure S5**. Ring spin density of the studied polycyclic molecules

# Supporting Information

**Optimized Cartesian Coordinates of the studied molecules**

**1**

| | | | |
|---|---|---|---|
| 6 | 2.149738000 | -0.733868000 | 0.000532000 |
| 6 | 1.054499000 | -1.538826000 | 0.000597000 |
| 5 | -0.291455000 | -0.829027000 | 0.000541000 |
| 6 | -1.853300000 | -1.176264000 | -0.000119000 |
| 6 | -2.486887000 | -0.000166000 | -0.000347000 |
| 6 | -1.522857000 | 1.162821000 | -0.000267000 |
| 6 | -0.248261000 | 0.735430000 | 0.000078000 |
| 6 | 1.002601000 | 1.449391000 | 0.000115000 |
| 6 | 2.141394000 | 0.728104000 | 0.000361000 |
| 1 | 3.138002000 | -1.185789000 | 0.000690000 |
| 1 | 1.215105000 | -2.610328000 | 0.000749000 |
| 1 | -2.374763000 | -2.122793000 | -0.000246000 |
| 1 | -3.559023000 | 0.152619000 | -0.000707000 |
| 1 | -1.861095000 | 2.194330000 | -0.000623000 |
| 1 | 1.033054000 | 2.533358000 | -0.000047000 |
| 1 | 3.101647000 | 1.226608000 | 0.000391000 |

**2**

| | | | |
|---|---|---|---|
| 6 | 2.086446000 | -0.783308000 | 0.000672000 |
| 6 | 0.965877000 | -1.544248000 | 0.000526000 |
| 5 | -0.345710000 | -0.767438000 | 0.000370000 |
| 7 | -1.801651000 | -1.148192000 | -0.000107000 |
| 6 | -2.403364000 | -0.031052000 | -0.000295000 |
| 6 | -1.516248000 | 1.219831000 | -0.000463000 |
| 6 | -0.245561000 | 0.803769000 | 0.000110000 |
| 6 | 1.037845000 | 1.466353000 | 0.000243000 |
| 6 | 2.134150000 | 0.687256000 | 0.000507000 |
| 1 | 3.058588000 | -1.267911000 | 0.000937000 |
| 1 | 1.071507000 | -2.621902000 | 0.000594000 |
| 1 | -3.490860000 | 0.048488000 | -0.000807000 |
| 1 | -1.907427000 | 2.230838000 | -0.000919000 |
| 1 | 1.122635000 | 2.546599000 | 0.000138000 |
| 1 | 3.117373000 | 1.139817000 | 0.000595000 |

**3**

| | | | |
|---|---|---|---|
| 6 | 2.041530000 | -0.775426000 | 0.000794000 |
| 7 | 0.988464000 | -1.523273000 | 0.000992000 |
| 5 | -0.245446000 | -0.787407000 | 0.001148000 |
| 6 | -1.797990000 | -1.178339000 | 0.000001000 |
| 6 | -2.457601000 | -0.016172000 | -0.000395000 |
| 6 | -1.524458000 | 1.172652000 | -0.000165000 |
| 6 | -0.240574000 | 0.775285000 | 0.000873000 |
| 6 | 1.018228000 | 1.465110000 | 0.000368000 |
| 6 | 2.124797000 | 0.692797000 | 0.000132000 |
| 1 | 3.011922000 | -1.279303000 | 0.000984000 |
| 1 | -2.292302000 | -2.138903000 | -0.000141000 |
| 1 | -3.532397000 | 0.113323000 | -0.001040000 |
| 1 | -1.891908000 | 2.194013000 | -0.001390000 |
| 1 | 1.086852000 | 2.547773000 | 0.000290000 |
| 1 | 3.115783000 | 1.125870000 | -0.000251000 |

**4**

| | | | |
|---|---|---|---|
| 6 | 2.115187000 | -0.700886000 | 0.000569000 |
| 6 | 1.067802000 | -1.556416000 | 0.000432000 |
| 5 | -0.297187000 | -0.867494000 | 0.000266000 |
| 6 | -1.857252000 | -1.197876000 | -0.000074000 |
| 6 | -2.473142000 | -0.011189000 | -0.000290000 |

| | | | |
|---|---|---|---|
| 6 | -1.500944000 | 1.145287000 | -0.000186000 |
| 6 | -0.236684000 | 0.690089000 | 0.000218000 |
| 7 | 0.923431000 | 1.458949000 | 0.000336000 |
| 6 | 2.006563000 | 0.771802000 | 0.000490000 |
| 1 | 3.134068000 | -1.077242000 | 0.000809000 |
| 1 | 1.268732000 | -2.621313000 | 0.000477000 |
| 1 | -2.391897000 | -2.136734000 | -0.000120000 |
| 1 | -3.543651000 | 0.151751000 | -0.000537000 |
| 1 | -1.807239000 | 2.184897000 | -0.000412000 |
| 1 | 2.942810000 | 1.323576000 | 0.000722000 |

**5**

| | | | |
|---|---|---|---|
| 6 | 2.164260000 | -0.722905000 | 0.000561000 |
| 6 | 1.070736000 | -1.540006000 | 0.000493000 |
| 5 | -0.260644000 | -0.828637000 | 0.000312000 |
| 6 | -1.866463000 | -1.097417000 | -0.000036000 |
| 7 | -2.503063000 | 0.011235000 | -0.000422000 |
| 6 | -1.527341000 | 1.094986000 | -0.000348000 |
| 6 | -0.237143000 | 0.713290000 | 0.000231000 |
| 6 | 0.998949000 | 1.445407000 | 0.000371000 |
| 6 | 2.146337000 | 0.732317000 | 0.000528000 |
| 1 | 3.154600000 | -1.169909000 | 0.000664000 |
| 1 | 1.238723000 | -2.609895000 | 0.000553000 |
| 1 | -2.447960000 | -2.014961000 | -0.000165000 |
| 1 | -1.933957000 | 2.100048000 | -0.000815000 |
| 1 | 1.018451000 | 2.529138000 | 0.000346000 |
| 1 | 3.102017000 | 1.239009000 | 0.000627000 |

**6**

| | | | |
|---|---|---|---|
| 6 | 2.137945000 | -0.745508000 | 0.000607000 |
| 6 | 1.072598000 | -1.577274000 | 0.000452000 |
| 5 | -0.294711000 | -0.891575000 | 0.000056000 |
| 6 | -1.832649000 | -1.172019000 | -0.000178000 |
| 6 | -2.384838000 | 0.046028000 | -0.000535000 |
| 7 | -1.464249000 | 1.195286000 | -0.000484000 |
| 6 | -0.282355000 | 0.705030000 | -0.000118000 |
| 6 | 0.963602000 | 1.451674000 | 0.000125000 |
| 6 | 2.096824000 | 0.734336000 | 0.000467000 |
| 1 | 3.139921000 | -1.165151000 | 0.000941000 |
| 1 | 1.257669000 | -2.644385000 | 0.000660000 |
| 1 | -2.417467000 | -2.078409000 | -0.000106000 |
| 1 | -3.438340000 | 0.293460000 | -0.000783000 |
| 1 | 0.962621000 | 2.534395000 | -0.000030000 |
| 1 | 3.055129000 | 1.237712000 | 0.000628000 |

**7**

| | | | |
|---|---|---|---|
| 6 | 1.984707000 | -0.808669000 | -0.027936000 |
| 7 | 0.910842000 | -1.515452000 | -0.000417000 |
| 5 | -0.292898000 | -0.732639000 | 0.042711000 |
| 7 | -1.736263000 | -1.151096000 | 0.015880000 |
| 6 | -2.364455000 | -0.051023000 | -0.032569000 |
| 6 | -1.507759000 | 1.225882000 | -0.036546000 |
| 6 | -0.233237000 | 0.839352000 | 0.060948000 |
| 6 | 1.055486000 | 1.485101000 | 0.032828000 |
| 6 | 2.120696000 | 0.666828000 | -0.032669000 |
| 1 | 2.938917000 | -1.342236000 | -0.032005000 |
| 1 | -3.452319000 | 0.003808000 | -0.067194000 |
| 1 | -1.920398000 | 2.223721000 | -0.130117000 |
| 1 | 1.170167000 | 2.562833000 | 0.056281000 |
| 1 | 3.128603000 | 1.058298000 | -0.067336000 |

**8**

| | | | |
|---|---|---|---|
| 6 | 2.072693000 | -0.809843000 | 0.000663000 |
| 6 | 0.972587000 | -1.592857000 | 0.000591000 |
| 5 | -0.348037000 | -0.823279000 | 0.000102000 |
| 7 | -1.797143000 | -1.112545000 | -0.000006000 |
| 6 | -2.309950000 | 0.048902000 | -0.000585000 |
| 7 | -1.445264000 | 1.259221000 | -0.000375000 |
| 6 | -0.262953000 | 0.769596000 | 0.000016000 |
| 6 | 1.013309000 | 1.459750000 | 0.000104000 |
| 6 | 2.101536000 | 0.674991000 | 0.000456000 |
| 1 | 3.055299000 | -1.272114000 | 0.000915000 |
| 1 | 1.097614000 | -2.668030000 | 0.000826000 |
| 1 | -3.378969000 | 0.245490000 | -0.000595000 |
| 1 | 1.076867000 | 2.540158000 | -0.000045000 |
| 1 | 3.086210000 | 1.125460000 | 0.000634000 |

**9**

| | | | |
|---|---|---|---|
| 6 | 2.112011000 | -0.759985000 | 0.000644000 |
| 6 | 0.993274000 | -1.533678000 | 0.000566000 |
| 5 | -0.298887000 | -0.754001000 | 0.000081000 |
| 7 | -1.809308000 | -1.096828000 | -0.000036000 |
| 7 | -2.444590000 | -0.041442000 | -0.000446000 |
| 6 | -1.510701000 | 1.161938000 | -0.000306000 |
| 6 | -0.232683000 | 0.790969000 | -0.000029000 |
| 6 | 1.040223000 | 1.470647000 | 0.000178000 |
| 6 | 2.147847000 | 0.704441000 | 0.000518000 |
| 1 | 3.086686000 | -1.239255000 | 0.000896000 |
| 1 | 1.104729000 | -2.610010000 | 0.000787000 |
| 1 | -1.986514000 | 2.133952000 | -0.000536000 |
| 1 | 1.109589000 | 2.551302000 | 0.000066000 |
| 1 | 3.125625000 | 1.167650000 | 0.000717000 |

**10**

| | | | |
|---|---|---|---|
| 6 | 2.017899000 | -0.725092000 | 0.150401000 |
| 6 | 0.935348000 | -1.523519000 | 0.030439000 |
| 5 | -0.371380000 | -0.798865000 | -0.306410000 |
| 7 | -1.810736000 | -1.153688000 | -0.275314000 |
| 6 | -2.350388000 | -0.041212000 | 0.030321000 |
| 6 | -1.422068000 | 1.157119000 | 0.098959000 |
| 6 | -0.218001000 | 0.766074000 | -0.351725000 |
| 7 | 0.976155000 | 1.454415000 | -0.310644000 |
| 6 | 2.005574000 | 0.735003000 | -0.055073000 |
| 1 | 2.994336000 | -1.146972000 | 0.366469000 |
| 1 | 1.056041000 | -2.594372000 | 0.143809000 |
| 1 | -3.420569000 | 0.060401000 | 0.208480000 |
| 1 | -1.689404000 | 2.119459000 | 0.517911000 |
| 1 | 2.966690000 | 1.240447000 | -0.022874000 |

**11**

| | | | |
|---|---|---|---|
| 6 | 1.996480000 | -0.717940000 | -0.142955000 |
| 7 | 0.980341000 | -1.491700000 | -0.031154000 |
| 5 | -0.274775000 | -0.844651000 | 0.208257000 |
| 6 | -1.805100000 | -1.201969000 | 0.043100000 |
| 6 | -2.415693000 | -0.013762000 | -0.047372000 |
| 6 | -1.447126000 | 1.122991000 | 0.029481000 |
| 6 | -0.222547000 | 0.711334000 | 0.421922000 |
| 7 | 0.944810000 | 1.427314000 | 0.323943000 |
| 6 | 1.983444000 | 0.752490000 | -0.016713000 |
| 1 | 2.990264000 | -1.164109000 | -0.226973000 |

| | | | |
|---|---|---|---|
| 1 | -2.327064000 | -2.147535000 | 0.055039000 |
| 1 | -3.476827000 | 0.155859000 | -0.176327000 |
| 1 | -1.671596000 | 2.114360000 | -0.348002000 |
| 1 | 2.934788000 | 1.270518000 | -0.088846000 |

**12**

| | | | |
|---|---|---|---|
| 6 | 2.034343000 | -0.796259000 | 0.000481000 |
| 7 | 1.010086000 | -1.569335000 | 0.001394000 |
| 5 | -0.244594000 | -0.850787000 | 0.001649000 |
| 6 | -1.776312000 | -1.169439000 | -0.000149000 |
| 6 | -2.351506000 | 0.037132000 | -0.000635000 |
| 7 | -1.451704000 | 1.210149000 | -0.001183000 |
| 6 | -0.264051000 | 0.740626000 | 0.001403000 |
| 6 | 0.991444000 | 1.467250000 | 0.000937000 |
| 6 | 2.089505000 | 0.696885000 | -0.000278000 |
| 1 | 3.021062000 | -1.267305000 | 0.001750000 |
| 1 | -2.338572000 | -2.089619000 | -0.000404000 |
| 1 | -3.408369000 | 0.267067000 | -0.001608000 |
| 1 | 1.030423000 | 2.549929000 | 0.000903000 |
| 1 | 3.081144000 | 1.130307000 | -0.001260000 |

**13**

| | | | |
|---|---|---|---|
| 6 | 2.060349000 | -0.731951000 | 0.096094000 |
| 7 | 1.001510000 | -1.477102000 | 0.015702000 |
| 5 | -0.221577000 | -0.788637000 | -0.150589000 |
| 6 | -1.804452000 | -1.109396000 | -0.025579000 |
| 7 | -2.451393000 | -0.007950000 | 0.062186000 |
| 6 | -1.491922000 | 1.068839000 | 0.091663000 |
| 6 | -0.233582000 | 0.757018000 | -0.284144000 |
| 6 | 1.006041000 | 1.457197000 | -0.160997000 |
| 6 | 2.118817000 | 0.718056000 | 0.073873000 |
| 1 | 3.027085000 | -1.237932000 | 0.138219000 |
| 1 | -2.368217000 | -2.034906000 | -0.094696000 |
| 1 | -1.843570000 | 2.009001000 | 0.502349000 |
| 1 | 1.070381000 | 2.534322000 | -0.269955000 |
| 1 | 3.089432000 | 1.184341000 | 0.169289000 |

**14**

| | | | |
|---|---|---|---|
| 6 | 2.155473000 | -0.739487000 | 0.000642000 |
| 6 | 1.085951000 | -1.576214000 | 0.000478000 |
| 5 | -0.259118000 | -0.880276000 | 0.000009000 |
| 6 | -1.837847000 | -1.087722000 | -0.000390000 |
| 7 | -2.430067000 | 0.039174000 | -0.000634000 |
| 7 | -1.440413000 | 1.166344000 | -0.000447000 |
| 6 | -0.258613000 | 0.685108000 | -0.000142000 |
| 6 | 0.978749000 | 1.444568000 | 0.000144000 |
| 6 | 2.117219000 | 0.732812000 | 0.000544000 |
| 1 | 3.155541000 | -1.163656000 | 0.000895000 |
| 1 | 1.270065000 | -2.642790000 | 0.000596000 |
| 1 | -2.465857000 | -1.970933000 | -0.000009000 |
| 1 | 0.969054000 | 2.526696000 | 0.000084000 |
| 1 | 3.073463000 | 1.239077000 | 0.000829000 |

**15**

| | | | |
|---|---|---|---|
| 6 | 2.122615000 | -0.667780000 | -0.094395000 |
| 6 | 1.071143000 | -1.525822000 | -0.042542000 |
| 5 | -0.273424000 | -0.863327000 | 0.190590000 |
| 6 | -1.865904000 | -1.109896000 | 0.075737000 |
| 7 | -2.457750000 | 0.013961000 | -0.075204000 |
| 6 | -1.449933000 | 1.049001000 | -0.089209000 |

| | | | |
|---|---|---|---|
| 6 | -0.216046000 | 0.670137000 | 0.317350000 |
| 7 | 0.928527000 | 1.435088000 | 0.284386000 |
| 6 | 2.017667000 | 0.778390000 | 0.086453000 |
| 1 | 3.128081000 | -1.040761000 | -0.262607000 |
| 1 | 1.261447000 | -2.584956000 | -0.169747000 |
| 1 | -2.474761000 | -2.005390000 | 0.159570000 |
| 1 | -1.731859000 | 2.013950000 | -0.493118000 |
| 1 | 2.941795000 | 1.348107000 | 0.068798000 |

**16**

| | | | |
|---|---|---|---|
| 6 | 2.106147000 | -0.733861000 | 0.000683000 |
| 6 | 1.077386000 | -1.600309000 | 0.000495000 |
| 5 | -0.297710000 | -0.916922000 | 0.000019000 |
| 6 | -1.829223000 | -1.178092000 | -0.000225000 |
| 6 | -2.356694000 | 0.053360000 | -0.000520000 |
| 7 | -1.425819000 | 1.190118000 | -0.000419000 |
| 6 | -0.257902000 | 0.679348000 | -0.000231000 |
| 7 | 0.910488000 | 1.454869000 | -0.000013000 |
| 6 | 1.979248000 | 0.756660000 | 0.000433000 |
| 1 | 3.132485000 | -1.088342000 | 0.001065000 |
| 1 | 1.285052000 | -2.663477000 | 0.000709000 |
| 1 | -2.430415000 | -2.073463000 | -0.000118000 |
| 1 | -3.406343000 | 0.316806000 | -0.000634000 |
| 1 | 2.921200000 | 1.300805000 | 0.000555000 |

**17**

| | | | |
|---|---|---|---|
| 6 | 2.003181000 | -0.764958000 | 0.098739000 |
| 7 | 0.925634000 | -1.472682000 | -0.003198000 |
| 5 | -0.260041000 | -0.728154000 | -0.180842000 |
| 7 | -1.752094000 | -1.104527000 | -0.092380000 |
| 7 | -2.391276000 | -0.059714000 | 0.049766000 |
| 6 | -1.469236000 | 1.132831000 | 0.099935000 |
| 6 | -0.226531000 | 0.823121000 | -0.272434000 |
| 6 | 1.046639000 | 1.479138000 | -0.164520000 |
| 6 | 2.117385000 | 0.692594000 | 0.080939000 |
| 1 | 2.952878000 | -1.301086000 | 0.152605000 |
| 1 | -1.894516000 | 2.054744000 | 0.475549000 |
| 1 | 1.153850000 | 2.551777000 | -0.274855000 |
| 1 | 3.106629000 | 1.114914000 | 0.190372000 |

**18**

| | | | |
|---|---|---|---|
| 6 | 2.058660000 | -0.662054000 | -0.146691000 |
| 6 | 1.043298000 | -1.522865000 | 0.061610000 |
| 5 | -0.312938000 | -0.860185000 | 0.329936000 |
| 7 | -1.723400000 | -1.206697000 | 0.209057000 |
| 6 | -2.231245000 | -0.081940000 | -0.108687000 |
| 7 | -1.388795000 | 1.107201000 | -0.225021000 |
| 6 | -0.260208000 | 0.740952000 | 0.260341000 |
| 7 | 0.896961000 | 1.489574000 | 0.229776000 |
| 6 | 1.957505000 | 0.812629000 | 0.008611000 |
| 1 | 3.057447000 | -1.024875000 | -0.365826000 |
| 1 | 1.236093000 | -2.588108000 | 0.021477000 |
| 1 | -3.286081000 | 0.072265000 | -0.317257000 |
| 1 | 2.898004000 | 1.357500000 | -0.004675000 |

**19**

| | | | |
|---|---|---|---|
| 6 | 1.923422000 | -0.735936000 | -0.184524000 |
| 7 | 0.886574000 | -1.469412000 | -0.034467000 |
| 5 | -0.336075000 | -0.803282000 | 0.284864000 |
| 7 | -1.755622000 | -1.171570000 | 0.160617000 |

| | | | |
|---|---|---|---|
| 6 | -2.316079000 | -0.042878000 | -0.036265000 |
| 6 | -1.409888000 | 1.159142000 | -0.010211000 |
| 6 | -0.213140000 | 0.770891000 | 0.471040000 |
| 7 | 0.979573000 | 1.430263000 | 0.393673000 |
| 6 | 1.977172000 | 0.733594000 | -0.011185000 |
| 1 | 2.895596000 | -1.217167000 | -0.311986000 |
| 1 | -3.388040000 | 0.056143000 | -0.198968000 |
| 1 | -1.649774000 | 2.121399000 | -0.445134000 |
| 1 | 2.950279000 | 1.210611000 | -0.073954000 |

**20**

| | | | |
|---|---|---|---|
| 6 | 2.004452000 | -0.761116000 | 0.040139000 |
| 7 | 0.976629000 | -1.523359000 | -0.026137000 |
| 5 | -0.263078000 | -0.799212000 | -0.094109000 |
| 7 | -1.687097000 | -1.178225000 | -0.006116000 |
| 6 | -2.266508000 | -0.052684000 | 0.051555000 |
| 7 | -1.467179000 | 1.208832000 | 0.064083000 |
| 6 | -0.273913000 | 0.793503000 | -0.123934000 |
| 6 | 0.982885000 | 1.515123000 | -0.061715000 |
| 6 | 2.064933000 | 0.733595000 | 0.072944000 |
| 1 | 2.988908000 | -1.235736000 | 0.034219000 |
| 1 | -3.342455000 | 0.085277000 | 0.108671000 |
| 1 | 1.038815000 | 2.596186000 | -0.094344000 |
| 1 | 3.055309000 | 1.161688000 | 0.159421000 |

**21**

| | | | |
|---|---|---|---|
| 6 | 2.030492000 | -0.687087000 | -0.152802000 |
| 7 | 1.007263000 | -1.466663000 | -0.077224000 |
| 5 | -0.237283000 | -0.842069000 | 0.175328000 |
| 6 | -1.809708000 | -1.122075000 | 0.009136000 |
| 7 | -2.414365000 | 0.006258000 | -0.052301000 |
| 6 | -1.433433000 | 1.041096000 | -0.023345000 |
| 6 | -0.209841000 | 0.688881000 | 0.449795000 |
| 7 | 0.940412000 | 1.414056000 | 0.368950000 |
| 6 | 2.000152000 | 0.764507000 | 0.024048000 |
| 1 | 3.021756000 | -1.130976000 | -0.258564000 |
| 1 | -2.400478000 | -2.030613000 | 0.074024000 |
| 1 | -1.678327000 | 1.979787000 | -0.505559000 |
| 1 | 2.939060000 | 1.305299000 | -0.028286000 |

**22**

| | | | |
|---|---|---|---|
| 6 | 2.006953000 | -0.741852000 | 0.161392000 |
| 7 | 0.984500000 | -1.465726000 | -0.069531000 |
| 5 | -0.289947000 | -0.920639000 | -0.286364000 |
| 6 | -1.779423000 | -1.194175000 | -0.029551000 |
| 6 | -2.292731000 | 0.040695000 | 0.111988000 |
| 7 | -1.354013000 | 1.105796000 | 0.128783000 |
| 6 | -0.237895000 | 0.727032000 | -0.379301000 |
| 7 | 0.939693000 | 1.381081000 | -0.263253000 |
| 6 | 1.979315000 | 0.734319000 | 0.108092000 |
| 1 | 3.001320000 | -1.190235000 | 0.156102000 |
| 1 | -2.362667000 | -2.100935000 | -0.041236000 |
| 1 | -3.326597000 | 0.312705000 | 0.274271000 |
| 1 | 2.933193000 | 1.252334000 | 0.132209000 |

**23**

| | | | |
|---|---|---|---|
| 6 | 2.070223000 | -0.759953000 | 0.043196000 |
| 7 | 1.021017000 | -1.499826000 | -0.065178000 |
| 5 | -0.223111000 | -0.852206000 | -0.175320000 |
| 6 | -1.775441000 | -1.104152000 | 0.050932000 |

| | | | |
|---|---|---|---|
| 7 | -2.361186000 | 0.026949000 | 0.132038000 |
| 7 | -1.376285000 | 1.089404000 | 0.238381000 |
| 6 | -0.247959000 | 0.723345000 | -0.249664000 |
| 6 | 0.998820000 | 1.439021000 | -0.109758000 |
| 6 | 2.106429000 | 0.701631000 | 0.111708000 |
| 1 | 3.044885000 | -1.249315000 | -0.000033000 |
| 1 | -2.393366000 | -1.991508000 | -0.011636000 |
| 1 | 1.037971000 | 2.518457000 | -0.187965000 |
| 1 | 3.073503000 | 1.171352000 | 0.227198000 |

**24**

| | | | |
|---|---|---|---|
| 6 | 2.117651000 | -0.703817000 | -0.134422000 |
| 6 | 1.085403000 | -1.562771000 | 0.023216000 |
| 5 | -0.266327000 | -0.894974000 | 0.214639000 |
| 6 | -1.823950000 | -1.093894000 | 0.032165000 |
| 7 | -2.357019000 | 0.047618000 | -0.165570000 |
| 7 | -1.332930000 | 1.078628000 | -0.290151000 |
| 6 | -0.225706000 | 0.674786000 | 0.216929000 |
| 7 | 0.932291000 | 1.429001000 | 0.186858000 |
| 6 | 2.007618000 | 0.761567000 | 0.003315000 |
| 1 | 3.120483000 | -1.073011000 | -0.322936000 |
| 1 | 1.277649000 | -2.627333000 | -0.030116000 |
| 1 | -2.482268000 | -1.948156000 | 0.134603000 |
| 1 | 2.940203000 | 1.318957000 | -0.006361000 |

**25**

| | | | |
|---|---|---|---|
| 6 | 2.091980000 | -0.819968000 | 0.000692000 |
| 6 | 0.966264000 | -1.580875000 | 0.000648000 |
| 5 | -0.302100000 | -0.771901000 | 0.000098000 |
| 7 | -1.801071000 | -1.035082000 | -0.000221000 |
| 7 | -2.425293000 | -0.017243000 | -0.000337000 |
| 7 | -1.376533000 | 1.332454000 | -0.000353000 |
| 6 | -0.236660000 | 0.791018000 | -0.000273000 |
| 6 | 1.069819000 | 1.442130000 | -0.000130000 |
| 6 | 2.152825000 | 0.648910000 | 0.000414000 |
| 1 | 3.059682000 | -1.312748000 | 0.000828000 |
| 1 | 1.059558000 | -2.658196000 | 0.000746000 |
| 1 | 1.145084000 | 2.521204000 | -0.000310000 |
| 1 | 3.141746000 | 1.088195000 | 0.000696000 |

**26**

| | | | |
|---|---|---|---|
| 6 | 1.961748000 | -0.713733000 | 0.176951000 |
| 7 | 0.917709000 | -1.452383000 | 0.045443000 |
| 5 | -0.290290000 | -0.790618000 | -0.248558000 |
| 7 | -1.766401000 | -1.105486000 | -0.108173000 |
| 7 | -2.343977000 | -0.021145000 | 0.065643000 |
| 6 | -1.393026000 | 1.092442000 | 0.076805000 |
| 6 | -0.204497000 | 0.758354000 | -0.462448000 |
| 7 | 0.974952000 | 1.427710000 | -0.387588000 |
| 6 | 1.994280000 | 0.744961000 | 0.000809000 |
| 1 | 2.932962000 | -1.193847000 | 0.305084000 |
| 1 | -1.677598000 | 2.006573000 | 0.579055000 |
| 1 | 2.955537000 | 1.243674000 | 0.062274000 |

**27**

| | | | |
|---|---|---|---|
| 6 | 2.052837000 | -0.729036000 | -0.114810000 |
| 7 | 1.021236000 | -1.470037000 | 0.053245000 |
| 5 | -0.240824000 | -0.897182000 | 0.217392000 |
| 6 | -1.772960000 | -1.116450000 | -0.045820000 |
| 7 | -2.306681000 | 0.042047000 | -0.170505000 |

| | | | |
|---|---|---|---|
| 7 | -1.313896000 | 1.030066000 | -0.278110000 |
| 6 | -0.219190000 | 0.704903000 | 0.322868000 |
| 7 | 0.944669000 | 1.388805000 | 0.238043000 |
| 6 | 2.009592000 | 0.738566000 | -0.062546000 |
| 1 | 3.045399000 | -1.179502000 | -0.111205000 |
| 1 | -2.417309000 | -1.981884000 | 0.040664000 |
| 1 | 2.952624000 | 1.274804000 | -0.085818000 |

**28**

| | | | |
|---|---|---|---|
| 6 | 2.012351000 | -0.698535000 | -0.121106000 |
| 7 | 0.984473000 | -1.438208000 | 0.069569000 |
| 5 | -0.257504000 | -0.836003000 | 0.273376000 |
| 7 | -1.693303000 | -1.126753000 | 0.028536000 |
| 7 | -2.245678000 | -0.049566000 | -0.194699000 |
| 7 | -1.302492000 | 1.060213000 | -0.327491000 |
| 6 | -0.231258000 | 0.762459000 | 0.316104000 |
| 7 | 0.938024000 | 1.438078000 | 0.256396000 |
| 6 | 1.988862000 | 0.776763000 | -0.060819000 |
| 1 | 3.002285000 | -1.154265000 | -0.147410000 |
| 1 | 2.940739000 | 1.296818000 | -0.088356000 |

**29**

| | | | |
|---|---|---|---|
| 6 | -3.465535000 | -0.140040000 | -0.000463000 |
| 6 | -2.688490000 | -1.266347000 | -0.000556000 |
| 5 | -1.197283000 | -1.033451000 | -0.000023000 |
| 6 | 0.182650000 | -1.889582000 | 0.000108000 |
| 6 | 1.177217000 | -0.985519000 | 0.000194000 |
| 6 | 2.613802000 | -1.191611000 | 0.000330000 |
| 6 | 3.439292000 | -0.134217000 | 0.000370000 |
| 6 | 2.914953000 | 1.227563000 | 0.000294000 |
| 6 | 1.595717000 | 1.506009000 | 0.000168000 |
| 6 | 0.644923000 | 0.426548000 | 0.000021000 |
| 6 | -0.723987000 | 0.437229000 | -0.000028000 |
| 6 | -1.656167000 | 1.518635000 | 0.000081000 |
| 6 | -2.980782000 | 1.223945000 | -0.000227000 |
| 1 | -4.547006000 | -0.247650000 | -0.000966000 |
| 1 | -3.194157000 | -2.224687000 | -0.001071000 |
| 1 | 0.371160000 | -2.955567000 | 0.000106000 |
| 1 | 2.995680000 | -2.204609000 | 0.000407000 |
| 1 | 4.512064000 | -0.268849000 | 0.000483000 |
| 1 | 3.630445000 | 2.040564000 | 0.000351000 |
| 1 | 1.244757000 | 2.529211000 | 0.000136000 |
| 1 | -1.329248000 | 2.553171000 | 0.000225000 |
| 1 | -3.715706000 | 2.017452000 | -0.000340000 |

**30**

| | | | |
|---|---|---|---|
| 6 | 3.130334000 | -1.153335000 | 0.000728000 |
| 6 | 1.865379000 | -1.650871000 | 0.000648000 |
| 5 | 0.744891000 | -0.620971000 | 0.000581000 |
| 6 | -0.838680000 | -0.564923000 | 0.000114000 |
| 6 | -1.853426000 | -1.491077000 | -0.000079000 |
| 6 | -3.195472000 | -1.064377000 | -0.000326000 |
| 6 | -3.500608000 | 0.279125000 | -0.000446000 |
| 6 | -2.476502000 | 1.241257000 | -0.000285000 |
| 6 | -1.170068000 | 0.812341000 | 0.000020000 |
| 6 | 0.061556000 | 1.639760000 | 0.000145000 |
| 6 | 1.178394000 | 0.876882000 | 0.000282000 |
| 6 | 2.568246000 | 1.248875000 | 0.000393000 |
| 6 | 3.487798000 | 0.261012000 | 0.000623000 |
| 1 | 3.971638000 | -1.840825000 | 0.000775000 |

| | | | |
|---|---|---|---|
| 1 | 1.746741000 | -2.728052000 | 0.000656000 |
| 1 | -1.633381000 | -2.551728000 | 0.000022000 |
| 1 | -3.992117000 | -1.796372000 | -0.000447000 |
| 1 | -4.534452000 | 0.597696000 | -0.000686000 |
| 1 | -2.718887000 | 2.296765000 | -0.000397000 |
| 1 | 0.022731000 | 2.725371000 | 0.000026000 |
| 1 | 2.873841000 | 2.289314000 | 0.000327000 |
| 1 | 4.542543000 | 0.503133000 | 0.000726000 |

**31**

| | | | |
|---|---|---|---|
| 6 | -3.312853000 | 0.140745000 | -0.000275000 |
| 6 | -2.486107000 | -0.971475000 | -0.000228000 |
| 6 | -1.098149000 | -0.836123000 | -0.000103000 |
| 6 | -0.269090000 | -2.048102000 | -0.000039000 |
| 6 | 1.083068000 | -2.102441000 | 0.000090000 |
| 5 | 1.812976000 | -0.764664000 | 0.000133000 |
| 6 | 3.305641000 | -0.226642000 | 0.000392000 |
| 6 | 3.203090000 | 1.106729000 | 0.000337000 |
| 6 | 1.767623000 | 1.573773000 | 0.000202000 |
| 6 | 0.924165000 | 0.530803000 | 0.000129000 |
| 6 | -0.529406000 | 0.456946000 | -0.000022000 |
| 6 | -1.373480000 | 1.564321000 | -0.000078000 |
| 6 | -2.751571000 | 1.411608000 | -0.000202000 |
| 1 | -4.387311000 | 0.019210000 | -0.000363000 |
| 1 | -2.918031000 | -1.964970000 | -0.000274000 |
| 1 | -0.842329000 | -2.972661000 | -0.000105000 |
| 1 | 1.548880000 | -3.080784000 | 0.000128000 |
| 1 | 4.253900000 | -0.744233000 | 0.000540000 |
| 1 | 4.025951000 | 1.811654000 | 0.000438000 |
| 1 | 1.510682000 | 2.627767000 | 0.000172000 |
| 1 | -0.943179000 | 2.557736000 | -0.000028000 |
| 1 | -3.390069000 | 2.285003000 | -0.000244000 |

**32**

| | | | |
|---|---|---|---|
| 6 | -0.252797000 | -1.412119000 | 0.000521000 |
| 6 | -1.485394000 | -0.702408000 | 0.000515000 |
| 6 | -2.762777000 | -1.152115000 | 0.000364000 |
| 6 | -3.743212000 | -0.020261000 | 0.000177000 |
| 6 | -3.121974000 | 1.165359000 | 0.000333000 |
| 5 | -1.539246000 | 0.851869000 | -0.000310000 |
| 6 | -0.222056000 | 1.552307000 | -0.000318000 |
| 6 | 0.925564000 | 0.769846000 | -0.000051000 |
| 6 | 2.227009000 | 1.392816000 | -0.000213000 |
| 6 | 3.372623000 | 0.676729000 | -0.000050000 |
| 6 | 3.359752000 | -0.760134000 | 0.000281000 |
| 6 | 2.179770000 | -1.410075000 | 0.000445000 |
| 6 | 0.920865000 | -0.705988000 | 0.000271000 |
| 1 | -0.221373000 | -2.497864000 | 0.000865000 |
| 1 | -3.077537000 | -2.192033000 | 0.000334000 |
| 1 | -4.811598000 | -0.192230000 | 0.000262000 |
| 1 | -3.662653000 | 2.101963000 | 0.000530000 |
| 1 | -0.065500000 | 2.625740000 | -0.000550000 |
| 1 | 2.260869000 | 2.474744000 | -0.000460000 |
| 1 | 4.325156000 | 1.191351000 | -0.000160000 |
| 1 | 4.294117000 | -1.303426000 | 0.000405000 |
| 1 | 2.145293000 | -2.492872000 | 0.000707000 |

**33**

| | | | |
|---|---|---|---|
| 6 | -1.089753000 | 2.001843000 | -0.000411000 |
| 6 | -1.767163000 | 0.727922000 | -0.000379000 |

| | | | |
|---|---|---|---|
| 6 | -3.065056000 | 0.386139000 | -0.000249000 |
| 6 | -3.219206000 | -1.116321000 | 0.000112000 |
| 6 | -2.043068000 | -1.755736000 | 0.000334000 |
| 5 | -0.948100000 | -0.609208000 | -0.000703000 |
| 6 | 0.578862000 | -0.491961000 | -0.000176000 |
| 6 | 1.456477000 | -1.578167000 | 0.000109000 |
| 6 | 2.831513000 | -1.398339000 | 0.000379000 |
| 6 | 3.344170000 | -0.107198000 | 0.000396000 |
| 6 | 2.494640000 | 0.990545000 | 0.000136000 |
| 6 | 1.110230000 | 0.825086000 | -0.000138000 |
| 6 | 0.254676000 | 2.023535000 | -0.000326000 |
| 1 | -1.648168000 | 2.931257000 | -0.000482000 |
| 1 | -3.918293000 | 1.055786000 | -0.000086000 |
| 1 | -4.196704000 | -1.584169000 | 0.000482000 |
| 1 | -1.961169000 | -2.833003000 | 0.000857000 |
| 1 | 1.046047000 | -2.581039000 | 0.000082000 |
| 1 | 3.498384000 | -2.249643000 | 0.000576000 |
| 1 | 4.415481000 | 0.047148000 | 0.000617000 |
| 1 | 2.911301000 | 1.990335000 | 0.000163000 |
| 1 | 0.775695000 | 2.973786000 | -0.000292000 |

**34**

| | | | |
|---|---|---|---|
| 6 | 1.247299000 | -0.346764000 | -0.155385000 |
| 6 | 1.533274000 | -1.698102000 | -0.363716000 |
| 6 | 2.829658000 | -2.181643000 | -0.272739000 |
| 6 | 3.881240000 | -1.322611000 | 0.018892000 |
| 6 | 3.623835000 | 0.027799000 | 0.190213000 |
| 6 | 2.328773000 | 0.535268000 | 0.093871000 |
| 6 | 2.132204000 | 1.979552000 | 0.217939000 |
| 6 | 0.961159000 | 2.644370000 | 0.073675000 |
| 5 | -0.268878000 | 1.797336000 | -0.212467000 |
| 6 | -1.839801000 | 2.028865000 | -0.300615000 |
| 6 | -2.378238000 | 0.804397000 | -0.169369000 |
| 6 | -3.777523000 | 0.420448000 | -0.101708000 |
| 6 | -4.109729000 | -0.858338000 | 0.121759000 |
| 6 | -3.086075000 | -1.880872000 | 0.328892000 |
| 6 | -1.769891000 | -1.616737000 | 0.250126000 |
| 6 | -1.319888000 | -0.276569000 | -0.064667000 |
| 6 | -0.073784000 | 0.246043000 | -0.227154000 |
| 1 | 0.735662000 | -2.374649000 | -0.634995000 |
| 1 | 3.020688000 | -3.233846000 | -0.437721000 |
| 1 | 4.891736000 | -1.699566000 | 0.094520000 |
| 1 | 4.439461000 | 0.711187000 | 0.394069000 |
| 1 | 3.043957000 | 2.535533000 | 0.424671000 |
| 1 | 0.975474000 | 3.723122000 | 0.176064000 |
| 1 | -2.436651000 | 2.925595000 | -0.395072000 |
| 1 | -4.532694000 | 1.186299000 | -0.224620000 |
| 1 | -5.148047000 | -1.156214000 | 0.177974000 |
| 1 | -3.416544000 | -2.884273000 | 0.565459000 |
| 1 | -1.047478000 | -2.396832000 | 0.440002000 |

**35**

| | | | |
|---|---|---|---|
| 6 | 0.601110000 | -1.135352000 | -0.000045000 |
| 6 | -0.413843000 | -0.255081000 | -0.000867000 |
| 6 | -1.880916000 | -0.455758000 | -0.000168000 |
| 6 | -2.649064000 | -1.596525000 | -0.000896000 |
| 6 | -4.045943000 | -1.480249000 | -0.000697000 |
| 6 | -4.666903000 | -0.239216000 | 0.000182000 |
| 6 | -3.901296000 | 0.923441000 | 0.000893000 |
| 6 | -2.513210000 | 0.822789000 | 0.000674000 |

| | | | |
|---|---|---|---|
| 6 | -1.534422000 | 1.883908000 | 0.000708000 |
| 5 | -0.160299000 | 1.265737000 | -0.000604000 |
| 6 | 1.247942000 | 1.733034000 | -0.000610000 |
| 6 | 2.281486000 | 0.748545000 | -0.000147000 |
| 6 | 3.638186000 | 1.135288000 | -0.000515000 |
| 6 | 4.657144000 | 0.209996000 | -0.000209000 |
| 6 | 4.347864000 | -1.151870000 | 0.000469000 |
| 6 | 3.028396000 | -1.565876000 | 0.000796000 |
| 6 | 1.973524000 | -0.650480000 | 0.000320000 |
| 1 | 0.465423000 | -2.214238000 | 0.000434000 |
| 1 | -2.187340000 | -2.575520000 | -0.001552000 |
| 1 | -4.652826000 | -2.376369000 | -0.001145000 |
| 1 | -5.746373000 | -0.178428000 | 0.000272000 |
| 1 | -4.376829000 | 1.896262000 | 0.001506000 |
| 1 | -1.868108000 | 2.916803000 | 0.001640000 |
| 1 | 1.581415000 | 2.767099000 | -0.001035000 |
| 1 | 3.865927000 | 2.194312000 | -0.000977000 |
| 1 | 5.689033000 | 0.533333000 | -0.000475000 |
| 1 | 5.142240000 | -1.886375000 | 0.000827000 |
| 1 | 2.797383000 | -2.624112000 | 0.001421000 |

**36**

| | | | |
|---|---|---|---|
| 6 | 0.147869000 | 2.034540000 | -0.000621000 |
| 6 | -0.559329000 | 0.785191000 | -0.000375000 |
| 6 | -1.880900000 | 0.476670000 | -0.000247000 |
| 6 | -3.052421000 | 1.321027000 | -0.000314000 |
| 6 | -4.274967000 | 0.762479000 | -0.000109000 |
| 6 | -4.469947000 | -0.688461000 | 0.000200000 |
| 6 | -3.434652000 | -1.540025000 | 0.000298000 |
| 6 | -2.074675000 | -1.026196000 | 0.000084000 |
| 6 | -0.906233000 | -1.695814000 | 0.000180000 |
| 5 | 0.219093000 | -0.569506000 | -0.000249000 |
| 6 | 1.746476000 | -0.496279000 | -0.000194000 |
| 6 | 2.592687000 | -1.608031000 | 0.000023000 |
| 6 | 3.972098000 | -1.469089000 | 0.000013000 |
| 6 | 4.522131000 | -0.192860000 | -0.000216000 |
| 6 | 3.705579000 | 0.929206000 | -0.000430000 |
| 6 | 2.316131000 | 0.805743000 | -0.000420000 |
| 6 | 1.496226000 | 2.024600000 | -0.000640000 |
| 1 | -0.380153000 | 2.981869000 | -0.000776000 |
| 1 | -2.932506000 | 2.396511000 | -0.000531000 |
| 1 | -5.156519000 | 1.390387000 | -0.000159000 |
| 1 | -5.484978000 | -1.063039000 | 0.000361000 |
| 1 | -3.588507000 | -2.611490000 | 0.000539000 |
| 1 | -0.854348000 | -2.775915000 | 0.000447000 |
| 1 | 2.152940000 | -2.598483000 | 0.000194000 |
| 1 | 4.613806000 | -2.339507000 | 0.000182000 |
| 1 | 5.597538000 | -0.069847000 | -0.000229000 |
| 1 | 4.151683000 | 1.916256000 | -0.000606000 |
| 1 | 2.039978000 | 2.961763000 | -0.000805000 |

**37**

| | | | |
|---|---|---|---|
| 6 | 0.164187000 | -1.268805000 | -0.034737000 |
| 6 | -0.359852000 | -2.576408000 | -0.196331000 |
| 6 | 0.453286000 | -3.673388000 | -0.284276000 |
| 6 | 1.839359000 | -3.492378000 | -0.220822000 |
| 6 | 2.382683000 | -2.232763000 | -0.094939000 |
| 6 | 1.589274000 | -1.078053000 | -0.005140000 |
| 6 | 2.216080000 | 0.253708000 | 0.079205000 |
| 6 | 3.609407000 | 0.386505000 | 0.229254000 |

| | | | |
|---|---|---|---|
| 6 | 4.211016000 | 1.625395000 | 0.258103000 |
| 6 | 3.440945000 | 2.782996000 | 0.137117000 |
| 6 | 2.072766000 | 2.674048000 | 0.011160000 |
| 6 | 1.431869000 | 1.428215000 | -0.012345000 |
| 5 | -0.096388000 | 1.256929000 | -0.064429000 |
| 6 | -1.283430000 | 2.090293000 | -0.382959000 |
| 6 | -2.508417000 | 1.291146000 | -0.221222000 |
| 6 | -3.814729000 | 1.692203000 | -0.268189000 |
| 6 | -4.836186000 | 0.763952000 | 0.062453000 |
| 6 | -4.510836000 | -0.509393000 | 0.431319000 |
| 6 | -3.149918000 | -0.931113000 | 0.458078000 |
| 6 | -2.168911000 | -0.053878000 | 0.100262000 |
| 6 | -0.673733000 | -0.136487000 | 0.056610000 |
| 1 | -1.428020000 | -2.693167000 | -0.289040000 |
| 1 | 0.035420000 | -4.661560000 | -0.417228000 |
| 1 | 2.497894000 | -4.347938000 | -0.294389000 |
| 1 | 3.457592000 | -2.149163000 | -0.093898000 |
| 1 | 4.241609000 | -0.481282000 | 0.340333000 |
| 1 | 5.283744000 | 1.696771000 | 0.378332000 |
| 1 | 3.915837000 | 3.755173000 | 0.155269000 |
| 1 | 1.467422000 | 3.569860000 | -0.058790000 |
| 1 | -1.371736000 | 3.114432000 | -0.733316000 |
| 1 | -4.070350000 | 2.709008000 | -0.538080000 |
| 1 | -5.871835000 | 1.074002000 | 0.030997000 |
| 1 | -5.285841000 | -1.214203000 | 0.699560000 |
| 1 | -2.928109000 | -1.936557000 | 0.785478000 |

**38**

| | | | |
|---|---|---|---|
| 6 | 1.680397000 | -0.439672000 | 0.000248000 |
| 6 | 2.425748000 | -1.617357000 | 0.000634000 |
| 6 | 3.810422000 | -1.585655000 | 0.000500000 |
| 6 | 4.480153000 | -0.366989000 | 0.000071000 |
| 6 | 3.754776000 | 0.812112000 | -0.000216000 |
| 6 | 2.358921000 | 0.799748000 | -0.000134000 |
| 6 | 1.640640000 | 2.078172000 | -0.000274000 |
| 6 | 0.298111000 | 2.244392000 | 0.000006000 |
| 5 | -0.539763000 | 0.971147000 | 0.000114000 |
| 6 | -2.063854000 | 0.566356000 | 0.000281000 |
| 6 | -3.259499000 | 1.246326000 | 0.000729000 |
| 6 | -4.471619000 | 0.533677000 | 0.000439000 |
| 6 | -4.467437000 | -0.845299000 | -0.000238000 |
| 6 | -3.256674000 | -1.556403000 | -0.000731000 |
| 6 | -2.077165000 | -0.848862000 | -0.000489000 |
| 6 | -0.690183000 | -1.380175000 | -0.000734000 |
| 6 | 0.225957000 | -0.389665000 | 0.000206000 |
| 1 | 1.911453000 | -2.569977000 | 0.001045000 |
| 1 | 4.370634000 | -2.511164000 | 0.000779000 |
| 1 | 5.561169000 | -0.339560000 | -0.000006000 |
| 1 | 4.271815000 | 1.764025000 | -0.000472000 |
| 1 | 2.290687000 | 2.950214000 | -0.000577000 |
| 1 | -0.085592000 | 3.257942000 | -0.000037000 |
| 1 | -3.278565000 | 2.329532000 | 0.001194000 |
| 1 | -5.411716000 | 1.068883000 | 0.000772000 |
| 1 | -5.404501000 | -1.386214000 | -0.000383000 |
| 1 | -3.259953000 | -2.639352000 | -0.001272000 |
| 1 | -0.495763000 | -2.448381000 | -0.001555000 |

**39**

| | | | |
|---|---|---|---|
| 6 | 1.182135000 | -1.562951000 | 0.000515000 |
| 6 | -0.144173000 | -1.048465000 | 0.000505000 |

| | | | |
|---|---|---|---|
| 6 | -1.335214000 | -1.704066000 | 0.000493000 |
| 6 | -2.475826000 | -0.768150000 | 0.000324000 |
| 6 | -3.817612000 | -1.079301000 | 0.000211000 |
| 6 | -4.749760000 | -0.030606000 | -0.000017000 |
| 6 | -4.318865000 | 1.278997000 | -0.000142000 |
| 6 | -2.943836000 | 1.584235000 | -0.000062000 |
| 6 | -2.014955000 | 0.572362000 | 0.000178000 |
| 5 | -0.425144000 | 0.482010000 | 0.000454000 |
| 6 | 0.779894000 | 1.373064000 | 0.000058000 |
| 6 | 2.026912000 | 0.772884000 | 0.000012000 |
| 6 | 3.223475000 | 1.584068000 | -0.000234000 |
| 6 | 4.461042000 | 1.046937000 | -0.000230000 |
| 6 | 4.656526000 | -0.378411000 | 0.000033000 |
| 6 | 3.587407000 | -1.196932000 | 0.000264000 |
| 6 | 2.236155000 | -0.688774000 | 0.000286000 |
| 1 | 1.377406000 | -2.631263000 | 0.000677000 |
| 1 | -1.475923000 | -2.781771000 | 0.000475000 |
| 1 | -4.152004000 | -2.109460000 | 0.000302000 |
| 1 | -5.808476000 | -0.251789000 | -0.000100000 |
| 1 | -5.045239000 | 2.081084000 | -0.000311000 |
| 1 | -2.631030000 | 2.621119000 | -0.000164000 |
| 1 | 0.772036000 | 2.458118000 | -0.000175000 |
| 1 | 3.094441000 | 2.658881000 | -0.000427000 |
| 1 | 5.328376000 | 1.694628000 | -0.000436000 |
| 1 | 5.660737000 | -0.778751000 | 0.000047000 |
| 1 | 3.715314000 | -2.272595000 | 0.000463000 |

**40**

| | | | |
|---|---|---|---|
| 6 | 0.917880000 | -2.540770000 | 0.000432000 |
| 6 | -0.232680000 | -1.670484000 | 0.000327000 |
| 6 | -1.554802000 | -1.942461000 | 0.000291000 |
| 6 | -2.368388000 | -0.699950000 | 0.000178000 |
| 6 | -3.739525000 | -0.588358000 | 0.000078000 |
| 6 | -4.312165000 | 0.693425000 | -0.000115000 |
| 6 | -3.513686000 | 1.817022000 | -0.000181000 |
| 6 | -2.113161000 | 1.691717000 | -0.000109000 |
| 6 | -1.531762000 | 0.444439000 | 0.000068000 |
| 5 | -0.056637000 | -0.122121000 | 0.000282000 |
| 6 | 1.377520000 | 0.419168000 | 0.000056000 |
| 6 | 1.720349000 | 1.774119000 | -0.000189000 |
| 6 | 3.042198000 | 2.188367000 | -0.000327000 |
| 6 | 4.052806000 | 1.233315000 | -0.000218000 |
| 6 | 3.743420000 | -0.117582000 | 0.000024000 |
| 6 | 2.415931000 | -0.549996000 | 0.000139000 |
| 6 | 2.146743000 | -1.994000000 | 0.000351000 |
| 1 | 0.801874000 | -3.618808000 | 0.000577000 |
| 1 | -2.012597000 | -2.926948000 | 0.000325000 |
| 1 | -4.370906000 | -1.468139000 | 0.000121000 |
| 1 | -5.389062000 | 0.798055000 | -0.000205000 |
| 1 | -3.966128000 | 2.799559000 | -0.000282000 |
| 1 | -1.505538000 | 2.588109000 | -0.000140000 |
| 1 | 0.930054000 | 2.514654000 | -0.000262000 |
| 1 | 3.288112000 | 3.241495000 | -0.000504000 |
| 1 | 5.089341000 | 1.544665000 | -0.000309000 |
| 1 | 4.540846000 | -0.850604000 | 0.000102000 |
| 1 | 3.019264000 | -2.636887000 | 0.000388000 |

**41**

| | | | |
|---|---|---|---|
| 6 | 1.214943000 | -1.445185000 | 0.000019000 |
| 6 | 1.661800000 | -2.769129000 | -0.000324000 |

| | | | |
|---|---|---|---|
| 6 | 3.006167000 | -3.078335000 | -0.000177000 |
| 6 | 3.935994000 | -2.045842000 | 0.000292000 |
| 6 | 3.510195000 | -0.730361000 | 0.000475000 |
| 6 | 2.153078000 | -0.386386000 | 0.000282000 |
| 6 | 1.736244000 | 1.052837000 | 0.000258000 |
| 6 | 2.699471000 | 2.067716000 | 0.000348000 |
| 6 | 2.345607000 | 3.406713000 | 0.000322000 |
| 6 | 1.011139000 | 3.792651000 | 0.000212000 |
| 6 | 0.042695000 | 2.808213000 | 0.000077000 |
| 6 | 0.372930000 | 1.447686000 | 0.000060000 |
| 5 | -0.691095000 | 0.351088000 | -0.000165000 |
| 6 | -2.267169000 | 0.261087000 | -0.000122000 |
| 6 | -3.317059000 | 1.152441000 | -0.000533000 |
| 6 | -4.641056000 | 0.682023000 | -0.000183000 |
| 6 | -4.897374000 | -0.673101000 | 0.000634000 |
| 6 | -3.842707000 | -1.598443000 | 0.001066000 |
| 6 | -2.550247000 | -1.126889000 | 0.000613000 |
| 6 | -1.293350000 | -1.917258000 | 0.000765000 |
| 6 | -0.204358000 | -1.121676000 | -0.000015000 |
| 1 | 0.928387000 | -3.565145000 | -0.000776000 |
| 1 | 3.330779000 | -4.110070000 | -0.000434000 |
| 1 | 4.995185000 | -2.264681000 | 0.000437000 |
| 1 | 4.268549000 | 0.036504000 | 0.000777000 |
| 1 | 3.752264000 | 1.834000000 | 0.000428000 |
| 1 | 3.125226000 | 4.157505000 | 0.000400000 |
| 1 | 0.740871000 | 4.839635000 | 0.000228000 |
| 1 | -1.003654000 | 3.086128000 | 0.000028000 |
| 1 | -3.141453000 | 2.220949000 | -0.001173000 |
| 1 | -5.462520000 | 1.385746000 | -0.000546000 |
| 1 | -5.919771000 | -1.027304000 | 0.000936000 |
| 1 | -4.048977000 | -2.661478000 | 0.001704000 |
| 1 | -1.313533000 | -3.002440000 | 0.001687000 |

**42**

| | | | |
|---|---|---|---|
| 6 | 0.539645000 | -0.478098000 | 0.000260000 |
| 6 | 1.378321000 | -1.581929000 | 0.000378000 |
| 6 | 2.757766000 | -1.409534000 | 0.000347000 |
| 6 | 3.292102000 | -0.130381000 | 0.000222000 |
| 6 | 2.453970000 | 0.980355000 | 0.000111000 |
| 6 | 1.072504000 | 0.828321000 | 0.000111000 |
| 6 | 0.221742000 | 2.040832000 | -0.000062000 |
| 6 | -1.128656000 | 2.097723000 | -0.000098000 |
| 5 | -1.830948000 | 0.746469000 | 0.000123000 |
| 7 | -3.208522000 | 0.223197000 | 0.000406000 |
| 6 | -3.024646000 | -1.035973000 | 0.000249000 |
| 7 | -1.659481000 | -1.600393000 | 0.000709000 |
| 6 | -0.914714000 | -0.558905000 | 0.000291000 |
| 1 | 0.935756000 | -2.569408000 | 0.000496000 |
| 1 | 3.412077000 | -2.270536000 | 0.000426000 |
| 1 | 4.364820000 | 0.007913000 | 0.000218000 |
| 1 | 2.880305000 | 1.975792000 | 0.000012000 |
| 1 | 0.793200000 | 2.965972000 | -0.000105000 |
| 1 | -1.603587000 | 3.070558000 | -0.000250000 |
| 1 | -3.828653000 | -1.767574000 | 0.001156000 |

**43**

| | | | |
|---|---|---|---|
| 6 | -1.116587000 | 2.012972000 | -0.000624000 |
| 6 | -1.779796000 | 0.730094000 | -0.000358000 |
| 7 | -3.033377000 | 0.452687000 | -0.000338000 |
| 6 | -3.047238000 | -1.019985000 | -0.000356000 |

| | | | |
|---|---|---|---|
| 7 | -1.960387000 | -1.688510000 | -0.000159000 |
| 5 | -0.946083000 | -0.626065000 | 0.000468000 |
| 6 | 0.580817000 | -0.528536000 | 0.000301000 |
| 6 | 1.461140000 | -1.605742000 | 0.000536000 |
| 6 | 2.837896000 | -1.405816000 | 0.000468000 |
| 6 | 3.335604000 | -0.113245000 | 0.000139000 |
| 6 | 2.471214000 | 0.979103000 | -0.000122000 |
| 6 | 1.094454000 | 0.794224000 | -0.000066000 |
| 6 | 0.228106000 | 1.998308000 | -0.000440000 |
| 1 | -1.674675000 | 2.940403000 | -0.000963000 |
| 1 | -4.034600000 | -1.475316000 | -0.000401000 |
| 1 | 1.056257000 | -2.610120000 | 0.000829000 |
| 1 | 3.513995000 | -2.249681000 | 0.000679000 |
| 1 | 4.404685000 | 0.053817000 | 0.000071000 |
| 1 | 2.877894000 | 1.982808000 | -0.000404000 |
| 1 | 0.763481000 | 2.941501000 | -0.000659000 |

**44**

| | | | |
|---|---|---|---|
| 6 | -1.515895000 | -0.035716000 | -0.000460000 |
| 6 | -2.896352000 | 0.149651000 | -0.000467000 |
| 6 | -3.436128000 | 1.421704000 | -0.000173000 |
| 6 | -2.576980000 | 2.511605000 | 0.000187000 |
| 6 | -1.200956000 | 2.333141000 | 0.000202000 |
| 6 | -0.622786000 | 1.062655000 | -0.000173000 |
| 6 | 0.879743000 | 0.905121000 | -0.000199000 |
| 6 | 1.699917000 | 2.032915000 | -0.000453000 |
| 6 | 3.085095000 | 1.924620000 | -0.000424000 |
| 6 | 3.708564000 | 0.686000000 | -0.000160000 |
| 6 | 2.917906000 | -0.449626000 | 0.000014000 |
| 6 | 1.523929000 | -0.361829000 | -0.000012000 |
| 5 | 0.626422000 | -1.588927000 | -0.000126000 |
| 7 | 0.801783000 | -3.047907000 | -0.000510000 |
| 6 | -0.406910000 | -3.457513000 | -0.000982000 |
| 7 | -1.527919000 | -2.506348000 | -0.000672000 |
| 6 | -0.937992000 | -1.366115000 | -0.000521000 |
| 1 | -3.524655000 | -0.731493000 | -0.000678000 |
| 1 | -4.507563000 | 1.566536000 | -0.000174000 |
| 1 | -2.977541000 | 3.516621000 | 0.000488000 |
| 1 | -0.586347000 | 3.219184000 | 0.000570000 |
| 1 | 1.281690000 | 3.026983000 | -0.000743000 |
| 1 | 3.680787000 | 2.828235000 | -0.000632000 |
| 1 | 4.787241000 | 0.612979000 | -0.000133000 |
| 1 | 3.368942000 | -1.434215000 | 0.000155000 |
| 1 | -0.687894000 | -4.507860000 | -0.000925000 |

**45**

| | | | |
|---|---|---|---|
| 6 | -3.394158000 | -0.202073000 | -0.167079000 |
| 6 | -2.630710000 | -1.298371000 | 0.129975000 |
| 5 | -1.118449000 | -1.085667000 | 0.281966000 |
| 7 | 0.019208000 | -1.748966000 | -0.110179000 |
| 6 | 1.124475000 | -0.877306000 | -0.044906000 |
| 6 | 2.458578000 | -1.213235000 | -0.086793000 |
| 6 | 3.408158000 | -0.176811000 | -0.043260000 |
| 6 | 3.036610000 | 1.152669000 | 0.023324000 |
| 6 | 1.676815000 | 1.500404000 | 0.064359000 |
| 6 | 0.753471000 | 0.483544000 | 0.041092000 |
| 6 | -0.698211000 | 0.458835000 | 0.216621000 |
| 7 | -1.559627000 | 1.415313000 | 0.005713000 |
| 6 | -2.842276000 | 1.107736000 | -0.108954000 |
| 1 | -4.466441000 | -0.270156000 | -0.299902000 |

| | | | |
|---|---|---|---|
| 1 | -3.144548000 | -2.238977000 | 0.299935000 |
| 1 | 2.762170000 | -2.248278000 | -0.159723000 |
| 1 | 4.459819000 | -0.429531000 | -0.081976000 |
| 1 | 3.791867000 | 1.925814000 | 0.025724000 |
| 1 | 1.364144000 | 2.535629000 | 0.107264000 |
| 1 | -3.527397000 | 1.949827000 | -0.097219000 |

**46**

| | | | |
|---|---|---|---|
| 6 | -0.838005000 | -0.752256000 | 0.037794000 |
| 6 | -2.131009000 | -1.373753000 | -0.167787000 |
| 6 | -3.267709000 | -0.656587000 | -0.206921000 |
| 6 | -3.252605000 | 0.778436000 | -0.035404000 |
| 6 | -2.095291000 | 1.430983000 | 0.158107000 |
| 6 | -0.825590000 | 0.729404000 | 0.184204000 |
| 7 | 0.247681000 | 1.440875000 | 0.354943000 |
| 6 | 1.429201000 | 0.773664000 | 0.377201000 |
| 6 | 2.582797000 | 1.101567000 | -0.247296000 |
| 6 | 3.540539000 | -0.060724000 | -0.130901000 |
| 7 | 3.043476000 | -1.139462000 | 0.326133000 |
| 5 | 1.603902000 | -0.791018000 | 0.406719000 |
| 6 | 0.296985000 | -1.510987000 | 0.140192000 |
| 1 | -2.151124000 | -2.449443000 | -0.286643000 |
| 1 | -4.216378000 | -1.153738000 | -0.360010000 |
| 1 | -4.188748000 | 1.319328000 | -0.061653000 |
| 1 | -2.050635000 | 2.503326000 | 0.288345000 |
| 1 | 2.765112000 | 1.993738000 | -0.834966000 |
| 1 | 4.592717000 | 0.040490000 | -0.394231000 |
| 1 | 0.184986000 | -2.589144000 | 0.086490000 |

**47**

| | | | |
|---|---|---|---|
| 6 | -1.065073000 | -0.774928000 | -0.000366000 |
| 6 | -2.439609000 | -0.984958000 | 0.000055000 |
| 6 | -3.313083000 | 0.095888000 | 0.000313000 |
| 6 | -2.825520000 | 1.396348000 | 0.000194000 |
| 6 | -1.454602000 | 1.617137000 | -0.000220000 |
| 6 | -0.561971000 | 0.547251000 | -0.000479000 |
| 5 | 0.966025000 | 0.617364000 | -0.001124000 |
| 7 | 1.975280000 | 1.710763000 | -0.000891000 |
| 6 | 3.082351000 | 1.074808000 | 0.000423000 |
| 6 | 3.014315000 | -0.447401000 | 0.000293000 |
| 6 | 1.715518000 | -0.763252000 | -0.000669000 |
| 7 | 1.099186000 | -2.016113000 | -0.000800000 |
| 6 | -0.180006000 | -1.969544000 | -0.000993000 |
| 1 | -2.830227000 | -1.995326000 | 0.000134000 |
| 1 | -4.380852000 | -0.079054000 | 0.000623000 |
| 1 | -3.513246000 | 2.231073000 | 0.000423000 |
| 1 | -1.062679000 | 2.626461000 | -0.000300000 |
| 1 | 4.042087000 | 1.592904000 | 0.000584000 |
| 1 | 3.878145000 | -1.098926000 | 0.001153000 |
| 1 | -0.704238000 | -2.924396000 | -0.000151000 |

**48**

| | | | |
|---|---|---|---|
| 6 | -2.205497000 | -0.770155000 | 0.000379000 |
| 6 | -3.575962000 | -1.131488000 | 0.000457000 |
| 6 | -4.573341000 | -0.188846000 | 0.000084000 |
| 6 | -4.225725000 | 1.165763000 | -0.000410000 |
| 6 | -2.899307000 | 1.562554000 | -0.000552000 |
| 6 | -1.864835000 | 0.631853000 | -0.000179000 |
| 7 | -0.562364000 | 1.158151000 | -0.000576000 |
| 6 | 0.367405000 | 0.273762000 | 0.000198000 |

| | | | |
|---|---|---|---|
| 6 | 1.839530000 | 0.478412000 | 0.000143000 |
| 6 | 2.632912000 | 1.589828000 | 0.000538000 |
| 6 | 4.033378000 | 1.416348000 | 0.000192000 |
| 6 | 4.589625000 | 0.151572000 | -0.000481000 |
| 6 | 3.778675000 | -0.990751000 | -0.000851000 |
| 6 | 2.401627000 | -0.831613000 | -0.000447000 |
| 7 | 1.479838000 | -1.878264000 | -0.000400000 |
| 5 | 0.211617000 | -1.277192000 | 0.000836000 |
| 6 | -1.210836000 | -1.771431000 | 0.000975000 |
| 1 | -3.819145000 | -2.186969000 | 0.000847000 |
| 1 | -5.612380000 | -0.485868000 | 0.000156000 |
| 1 | -5.004288000 | 1.917508000 | -0.000698000 |
| 1 | -2.628830000 | 2.609126000 | -0.000987000 |
| 1 | 2.196525000 | 2.580306000 | 0.000939000 |
| 1 | 4.678164000 | 2.284906000 | 0.000385000 |
| 1 | 5.665721000 | 0.042035000 | -0.000742000 |
| 1 | 4.201732000 | -1.986068000 | -0.001366000 |
| 1 | -1.549536000 | -2.803580000 | 0.001559000 |

**49**

| | | | |
|---|---|---|---|
| 6 | -2.230649000 | -0.741067000 | 0.000224000 |
| 6 | -3.609204000 | -0.920600000 | -0.000480000 |
| 6 | -4.459639000 | 0.179578000 | -0.000870000 |
| 6 | -3.942351000 | 1.467164000 | -0.000530000 |
| 6 | -2.565046000 | 1.656676000 | 0.000178000 |
| 6 | -1.696799000 | 0.568971000 | 0.000552000 |
| 5 | -0.162361000 | 0.613096000 | 0.001450000 |
| 7 | 0.860485000 | 1.666373000 | 0.001418000 |
| 6 | 1.967072000 | 0.997094000 | 0.000761000 |
| 6 | 3.306543000 | 1.559298000 | 0.000101000 |
| 6 | 4.365340000 | 0.730668000 | -0.000716000 |
| 6 | 4.251057000 | -0.728615000 | -0.000978000 |
| 6 | 3.053606000 | -1.336826000 | -0.000374000 |
| 6 | 1.864550000 | -0.522397000 | 0.000632000 |
| 6 | 0.547220000 | -0.804200000 | 0.001513000 |
| 7 | -0.095018000 | -2.033853000 | 0.001323000 |
| 6 | -1.376208000 | -1.956088000 | 0.000708000 |
| 1 | -4.021842000 | -1.922280000 | -0.000731000 |
| 1 | -5.531002000 | 0.028614000 | -0.001430000 |
| 1 | -4.610431000 | 2.317784000 | -0.000840000 |
| 1 | -2.150342000 | 2.656854000 | 0.000444000 |
| 1 | 3.413353000 | 2.635029000 | 0.000248000 |
| 1 | 5.364644000 | 1.148672000 | -0.001221000 |
| 1 | 5.164240000 | -1.307889000 | -0.001658000 |
| 1 | 2.954206000 | -2.413996000 | -0.000553000 |
| 1 | -1.922225000 | -2.898460000 | 0.000629000 |

**50**

| | | | |
|---|---|---|---|
| 5 | 1.124104000 | -0.951733000 | -0.000366000 |
| 7 | -0.036177000 | -1.731927000 | 0.000517000 |
| 6 | -1.122156000 | -0.832811000 | 0.000127000 |
| 6 | -2.444639000 | -1.173804000 | 0.000248000 |
| 6 | -3.425149000 | -0.136910000 | -0.000369000 |
| 6 | -3.065778000 | 1.179687000 | -0.000947000 |
| 6 | -1.684111000 | 1.537577000 | -0.000939000 |
| 6 | -0.749984000 | 0.547711000 | -0.000467000 |
| 6 | 0.715596000 | 0.549136000 | -0.000573000 |
| 6 | 1.690647000 | 1.534107000 | -0.000210000 |
| 6 | 2.990920000 | 1.061197000 | 0.000170000 |
| 6 | 3.355435000 | -0.347806000 | -0.000033000 |

| | | | |
|---|---|---|---|
| 7 | 2.530948000 | -1.336797000 | -0.000272000 |
| 1 | -2.736702000 | -2.214883000 | 0.000775000 |
| 1 | -4.472782000 | -0.406927000 | -0.000280000 |
| 1 | -3.819505000 | 1.954140000 | -0.001309000 |
| 1 | -1.396633000 | 2.582082000 | -0.001338000 |
| 1 | 1.492642000 | 2.600235000 | -0.000091000 |
| 1 | 3.811288000 | 1.771328000 | 0.000542000 |
| 1 | 4.427235000 | -0.549202000 | 0.000216000 |

**51**

| | | | |
|---|---|---|---|
| 5 | -1.739845000 | -0.698761000 | -0.000732000 |
| 7 | -3.187496000 | -0.328858000 | -0.000075000 |
| 6 | -3.166248000 | 0.941601000 | -0.000075000 |
| 6 | -1.788041000 | 1.608376000 | 0.000309000 |
| 6 | -0.883142000 | 0.627608000 | -0.000762000 |
| 6 | 0.565312000 | 0.533646000 | -0.000510000 |
| 6 | 1.456975000 | 1.600591000 | -0.000238000 |
| 6 | 2.825807000 | 1.370306000 | 0.000190000 |
| 6 | 3.321443000 | 0.072250000 | 0.000291000 |
| 6 | 2.440897000 | -1.000026000 | -0.000083000 |
| 6 | 1.067587000 | -0.783058000 | -0.000549000 |
| 6 | 0.163191000 | -1.960507000 | -0.001385000 |
| 7 | -1.117777000 | -1.996994000 | -0.000764000 |
| 1 | -4.077499000 | 1.540175000 | 0.000976000 |
| 1 | -1.649370000 | 2.683091000 | 0.001444000 |
| 1 | 1.075747000 | 2.613603000 | -0.000339000 |
| 1 | 3.510929000 | 2.207500000 | 0.000462000 |
| 1 | 4.388611000 | -0.101129000 | 0.000632000 |
| 1 | 2.818537000 | -2.015600000 | -0.000126000 |
| 1 | 0.702585000 | -2.914013000 | -0.000764000 |

**52**

| | | | |
|---|---|---|---|
| 5 | -1.504231000 | -0.754820000 | -0.211126000 |
| 7 | -2.934153000 | -1.200747000 | -0.070577000 |
| 6 | -3.560841000 | -0.126601000 | 0.172426000 |
| 6 | -2.713464000 | 1.137346000 | 0.186544000 |
| 6 | -1.488679000 | 0.828426000 | -0.272854000 |
| 6 | -0.241686000 | 1.509237000 | -0.225345000 |
| 6 | 0.892931000 | 0.769067000 | -0.035744000 |
| 6 | 2.182022000 | 1.411039000 | 0.040621000 |
| 6 | 3.320516000 | 0.694690000 | 0.097585000 |
| 6 | 3.258608000 | -0.741681000 | 0.065480000 |
| 6 | 2.084178000 | -1.405012000 | -0.005912000 |
| 6 | 0.815811000 | -0.713641000 | -0.020026000 |
| 7 | -0.285048000 | -1.424797000 | -0.061285000 |
| 1 | -4.634147000 | -0.091308000 | 0.353638000 |
| 1 | -3.045775000 | 2.077613000 | 0.614249000 |
| 1 | -0.164381000 | 2.584537000 | -0.358023000 |
| 1 | 2.204684000 | 2.494043000 | 0.050325000 |
| 1 | 4.282087000 | 1.184598000 | 0.155901000 |
| 1 | 4.185177000 | -1.300928000 | 0.097609000 |
| 1 | 2.038389000 | -2.484663000 | -0.027386000 |

**53**

| | | | |
|---|---|---|---|
| 5 | -0.250968000 | -1.669515000 | -0.034749000 |
| 7 | -1.694728000 | -2.002204000 | -0.044520000 |
| 6 | -2.262400000 | -0.853848000 | -0.024673000 |
| 6 | -3.701425000 | -0.619310000 | -0.014776000 |
| 6 | -4.162780000 | 0.638227000 | 0.025258000 |
| 6 | -3.278953000 | 1.805831000 | 0.062028000 |

| | | | |
|---|---|---|---|
| 6 | -1.941237000 | 1.691495000 | 0.047482000 |
| 6 | -1.342890000 | 0.375754000 | -0.009555000 |
| 6 | -0.078429000 | -0.089827000 | -0.045107000 |
| 6 | 1.256736000 | 0.471333000 | -0.028642000 |
| 6 | 1.593695000 | 1.822788000 | -0.070237000 |
| 6 | 2.923138000 | 2.222540000 | -0.044404000 |
| 6 | 3.940889000 | 1.281226000 | 0.021063000 |
| 6 | 3.621539000 | -0.069656000 | 0.054379000 |
| 6 | 2.296375000 | -0.483451000 | 0.028053000 |
| 6 | 2.017726000 | -1.941977000 | 0.043708000 |
| 7 | 0.891298000 | -2.547935000 | 0.016223000 |
| 1 | -4.355783000 | -1.479615000 | -0.036074000 |
| 1 | -5.229871000 | 0.820730000 | 0.035579000 |
| 1 | -3.739072000 | 2.784028000 | 0.103924000 |
| 1 | -1.318926000 | 2.574376000 | 0.081285000 |
| 1 | 0.815130000 | 2.569318000 | -0.130030000 |
| 1 | 3.165295000 | 3.276391000 | -0.076676000 |
| 1 | 4.974623000 | 1.597113000 | 0.042268000 |
| 1 | 4.407858000 | -0.813771000 | 0.099298000 |
| 1 | 2.928363000 | -2.550344000 | 0.075354000 |

**54**

| | | | |
|---|---|---|---|
| 5 | -0.145075000 | -1.057623000 | -0.000186000 |
| 7 | -1.424981000 | -1.751447000 | 0.000253000 |
| 6 | -2.370159000 | -0.787744000 | 0.000096000 |
| 6 | -3.752491000 | -1.039164000 | 0.000319000 |
| 6 | -4.629987000 | 0.034080000 | 0.000004000 |
| 6 | -4.138869000 | 1.336355000 | -0.000494000 |
| 6 | -2.759879000 | 1.616918000 | -0.000682000 |
| 6 | -1.875070000 | 0.571602000 | -0.000314000 |
| 6 | -0.408226000 | 0.459545000 | -0.000403000 |
| 6 | 0.628037000 | 1.307875000 | -0.000136000 |
| 6 | 1.967649000 | 0.725618000 | -0.000073000 |
| 6 | 3.087616000 | 1.544216000 | 0.000031000 |
| 6 | 4.374585000 | 1.015589000 | 0.000097000 |
| 6 | 4.569166000 | -0.363835000 | 0.000065000 |
| 6 | 3.476802000 | -1.204658000 | -0.000026000 |
| 6 | 2.151572000 | -0.707342000 | -0.000105000 |
| 7 | 1.145218000 | -1.610333000 | -0.000168000 |
| 1 | -4.097330000 | -2.064294000 | 0.000713000 |
| 1 | -5.697698000 | -0.133732000 | 0.000145000 |
| 1 | -4.840554000 | 2.160768000 | -0.000729000 |
| 1 | -2.418230000 | 2.643591000 | -0.001084000 |
| 1 | 0.553049000 | 2.392717000 | 0.000083000 |
| 1 | 2.950824000 | 2.618990000 | 0.000048000 |
| 1 | 5.226609000 | 1.682302000 | 0.000171000 |
| 1 | 5.571089000 | -0.770437000 | 0.000123000 |
| 1 | 3.587932000 | -2.280558000 | -0.000047000 |

**55**

| | | | |
|---|---|---|---|
| 6 | 0.293387000 | -2.075196000 | 0.000428000 |
| 6 | -1.061222000 | -2.157707000 | 0.000390000 |
| 5 | -1.786510000 | -0.830355000 | 0.000083000 |
| 6 | -3.214433000 | -0.141049000 | 0.000254000 |
| 7 | -3.087182000 | 1.127510000 | -0.000207000 |
| 7 | -1.649209000 | 1.526764000 | -0.000164000 |
| 6 | -0.923963000 | 0.478643000 | 0.000022000 |
| 6 | 0.536994000 | 0.435935000 | 0.000075000 |
| 6 | 1.347044000 | 1.561145000 | -0.000141000 |
| 6 | 2.729770000 | 1.424007000 | -0.000050000 |

| | | | |
|---|---|---|---|
| 6 | 3.305078000 | 0.161008000 | 0.000225000 |
| 6 | 2.498711000 | -0.969232000 | 0.000400000 |
| 6 | 1.110845000 | -0.851877000 | 0.000307000 |
| 1 | 0.879784000 | -2.991204000 | 0.000592000 |
| 1 | -1.510791000 | -3.142471000 | 0.000591000 |
| 1 | -4.224932000 | -0.532143000 | 0.000162000 |
| 1 | 0.881381000 | 2.537644000 | -0.000355000 |
| 1 | 3.358611000 | 2.304023000 | -0.000196000 |
| 1 | 4.381247000 | 0.055071000 | 0.000293000 |
| 1 | 2.948891000 | -1.954214000 | 0.000592000 |

**56**

| | | | |
|---|---|---|---|
| 6 | -0.996650000 | -0.782813000 | -0.085046000 |
| 6 | -2.258100000 | -1.396485000 | -0.042158000 |
| 6 | -3.421296000 | -0.654736000 | 0.054321000 |
| 6 | -3.345014000 | 0.731738000 | 0.104698000 |
| 6 | -2.109978000 | 1.367863000 | 0.057663000 |
| 6 | -0.929101000 | 0.644044000 | -0.045576000 |
| 6 | 0.351853000 | 1.349637000 | -0.100953000 |
| 6 | 1.474435000 | 0.645549000 | -0.277325000 |
| 7 | 2.813260000 | 1.134476000 | -0.255320000 |
| 7 | 3.597851000 | 0.199805000 | 0.053790000 |
| 6 | 2.933354000 | -1.045552000 | 0.183777000 |
| 5 | 1.501654000 | -0.897568000 | -0.199604000 |
| 6 | 0.192610000 | -1.582456000 | -0.174382000 |
| 1 | -2.305957000 | -2.477795000 | -0.077338000 |
| 1 | -4.381510000 | -1.149895000 | 0.092882000 |
| 1 | -4.248852000 | 1.321429000 | 0.177932000 |
| 1 | -2.060839000 | 2.448821000 | 0.093747000 |
| 1 | 0.352877000 | 2.422258000 | 0.065338000 |
| 1 | 3.525182000 | -1.823883000 | 0.648131000 |
| 1 | 0.027820000 | -2.650935000 | -0.281877000 |

**57**

| | | | |
|---|---|---|---|
| 6 | -1.131030000 | -0.803062000 | -0.000178000 |
| 6 | -2.509460000 | -0.990018000 | -0.000219000 |
| 6 | -3.375925000 | 0.097233000 | -0.000149000 |
| 6 | -2.885492000 | 1.395651000 | -0.000040000 |
| 6 | -1.513578000 | 1.599079000 | 0.000000000 |
| 6 | -0.621727000 | 0.524220000 | -0.000081000 |
| 5 | 0.894785000 | 0.635812000 | 0.000007000 |
| 6 | 2.102403000 | 1.661290000 | 0.000685000 |
| 7 | 3.207249000 | 1.024089000 | 0.000493000 |
| 7 | 2.988804000 | -0.453122000 | 0.000238000 |
| 6 | 1.733212000 | -0.685365000 | 0.000166000 |
| 6 | 1.082296000 | -1.980581000 | -0.000047000 |
| 6 | -0.259314000 | -1.998096000 | -0.000206000 |
| 1 | -2.912227000 | -1.995102000 | -0.000283000 |
| 1 | -4.444446000 | -0.074112000 | -0.000169000 |
| 1 | -3.566069000 | 2.235637000 | -0.000001000 |
| 1 | -1.119673000 | 2.608099000 | 0.000073000 |
| 1 | 2.159787000 | 2.743339000 | 0.001051000 |
| 1 | 1.664949000 | -2.892497000 | -0.000081000 |
| 1 | -0.779444000 | -2.948895000 | -0.000359000 |

**58**

| | | | |
|---|---|---|---|
| 6 | 0.870085000 | -0.958834000 | -0.000025000 |
| 6 | 1.659267000 | -2.109946000 | -0.000177000 |
| 6 | 3.045341000 | -2.042432000 | -0.000085000 |
| 6 | 3.707574000 | -0.822160000 | 0.000158000 |

| | | | |
|---|---|---|---|
| 6 | 2.950992000 | 0.333374000 | 0.000299000 |
| 6 | 1.550928000 | 0.291559000 | 0.000201000 |
| 5 | 0.695170000 | 1.539596000 | 0.000129000 |
| 6 | 0.758534000 | 3.119989000 | 0.000130000 |
| 7 | -0.427010000 | 3.592320000 | 0.000051000 |
| 7 | -1.447549000 | 2.508011000 | 0.000024000 |
| 6 | -0.853027000 | 1.378873000 | 0.000033000 |
| 6 | -1.478151000 | 0.061724000 | -0.000097000 |
| 6 | -2.863592000 | -0.076919000 | -0.000131000 |
| 6 | -3.446195000 | -1.330062000 | -0.000128000 |
| 6 | -2.629964000 | -2.452844000 | -0.000068000 |
| 6 | -1.250472000 | -2.320996000 | -0.000021000 |
| 6 | -0.630660000 | -1.068594000 | -0.000048000 |
| 1 | 1.210764000 | -3.090578000 | -0.000392000 |
| 1 | 3.614316000 | -2.963138000 | -0.000221000 |
| 1 | 4.787765000 | -0.781463000 | 0.000221000 |
| 1 | 3.439610000 | 1.300072000 | 0.000464000 |
| 1 | 1.571963000 | 3.835745000 | 0.000437000 |
| 1 | -3.464089000 | 0.823080000 | -0.000139000 |
| 1 | -4.522467000 | -1.434501000 | -0.000152000 |
| 1 | -3.067133000 | -3.442267000 | -0.000030000 |
| 1 | -0.664603000 | -3.226511000 | 0.000069000 |

******************************************************************************************
